# An enhanced parrot optimizer with multiple strategies for wireless sensor network node deployment

**DOI:** 10.1038/s41598-025-33513-6

**Published:** 2026-01-02

**Authors:** Li Lan, Zhang Qi

**Affiliations:** 1 Dazhou Vocational and Technical College, Dazhou, 635000 China; 2 Chengdu Technological University, Chengdu, 611730 China

**Keywords:** Parrot optimizer, Chaotic sequence, Nonlinear decreasing factor, T-distribution mutation strategy, Alert updating strategy, Differential evolution, CEC2017, Wireless sensor network, Engineering, Mathematics and computing

## Abstract

This paper presents an Enhanced parrot Optimizer (EPO), a novel metaheuristic algorithm that synergistically integrates multiple advanced strategies to address the critical limitations of the original parrot Optimizer (PO)—namely, poor initial population diversity, susceptibility to premature convergence, slow convergence speed, and the absence of an effective restart mechanism. EPO introduces five key innovations across its behavioral phases: (1) Cubic chaotic mapping is employed to generate a high-quality, well-distributed initial population, enhancing global exploration from the outset; (2) a risk-aware alert-contraction mechanism—inspired by predator-avoidance strategies in swarm intelligence—is embedded in the staying behavior to proactively detect and escape local optima; (3) a nonlinear decay factor dynamically balances exploration and exploitation during the communicating behavior phase; (4) a dual-layer intelligent judgment system governs the “fear of strangers” behavior, combining elite-guided convergence (outer layer) with diversity preservation (inner layer); and (5) a hybrid local restart mechanism is activated upon three consecutive generations of stagnation, applying t-distribution perturbation to low-fitness individuals for large-scale jumps and Differential Evolution (DE) to high-fitness individuals for refined local search.The performance of EPO is rigorously evaluated on the CEC2017 benchmark suite at 30, 50, and 100 dimensions, with comprehensive comparisons against eleven state-of-the-art metaheuristic algorithms—including PO, MPO, SMO, BTO, HOA, HHO, DBO, BKA, PSO, DE, and the Ivy algorithm. EPO achieves the best mean ranking across all dimensionalities (2.31 at 30D, 2.03 at 50D, and 1.83 at 100D), consistently outperforming all competitors in terms of solution accuracy, convergence speed, and stability. Wilcoxon signed-rank tests at a significance level of α = 0.05 confirm that the EPO algorithm achieves statistically significantly better performance than its competitors in 212 cases (81.2%) at 30D, 225 cases (86.2%) at 50D, 225 cases (86.2%) at 50D, 239 cases (91.6%) at 100D. Furthermore, EPO is successfully applied to the real-world Wireless Sensor Network (WSN) node deployment problem, consistently outperforming the original PO across different network scales: with 20 nodes, coverage improves from 0.7765 to 0.8284 (+ 6.7%); with 30 nodes, from 0.9285 to 0.9871 (+ 6.3%); and with 40 nodes, from 0.9826 to 0.9992 (+ 1.7%).Collectively, these findings validate that EPO not only advances the theoretical foundation of parrot-inspired optimization but also provides a powerful and reliable tool for solving complex real-world optimization problems.

## Introduction

### Background and motivation

Global optimization problems are prevalent across the fields of Medicine^1^, Engineering^2^, and Industry^3^. These problems are typically highly complex, exhibiting characteristics such as nonlinearity, non-convexity, multimodality, non-differentiable objective functions, and high dimensionality with large-scale decision spaces^4^. Traditional exact optimization methods are often ill-suited to address such real-world challenges. Extensive research has demonstrated that metaheuristic algorithms (MHs) can effectively escape local optima and approximate global optima within reasonable computational time, making them a powerful and practical approach for tackling these problems^5,6^. Metaheuristic algorithms draw inspiration from natural phenomena or social behaviors. Based on their underlying operational mechanisms, they are commonly categorized into four main classes^7–9^: (1) Evolutionary Algorithms (EAs), (2) Swarm Intelligence Algorithms (SIs), (3) Physics-based Algorithms, and (4) Human-based Algorithms. According to the “No Free Lunch” (NFL) theorem^10^, no single algorithm universally outperforms all others across every problem domain, which has motivated the continuous development of novel metaheuristics.

Evolutionary Algorithms simulate the principle of “survival of the fittest,” evolving a population of candidate solutions toward optimality through selection, crossover, and mutation. Representative examples include the Genetic Algorithm (GA)^11^ and Differential Evolution (DE)^12^. Recent advances in this category have introduced algorithms such as the Ant Lion Optimizer (ALO)^13^ and Plant Competition Optimization (PCO)^14^.

Swarm Intelligence Algorithms mimic collective behaviors and information exchange among individuals in decentralized, self-organized systems to iteratively refine near-optimal solutions. Classic instances include Ant Colony Optimization (ACO)^15^ and Particle Swarm Optimization (PSO)^16^. More recently proposed SI algorithms encompass the Starling Murmuration Optimizer (SMO)⁷, Golden Jackal Optimization (GJO)^17^, Dwarf Mongoose Optimization (DMO)^18^, Artificial Protozoa Optimizer (APO)^19^, Dung Beetle optimizer(DBO)^20^,Black-winged Kite Algorithm(BKA)^21^, Sperm Swarm Optimization (SSO)^22^, Draco lizard optimizer(DLO)^23^, **Gyro fireworks algorithm(GFA)**^24^.

Human-based Algorithms model human social interactions, cooperative strategies, or cultural evolution. Notable examples include Tabu Search (TS)^15^, Imperialist Competitive Algorithm (ICA)^25^. Recent contributions in this class include the Cooperation search algorithm(CAS)^26^, Five Phases Algorithm (FPA)^27^, Educational Competition Optimizer^28^, Hiking Optimization Algorithm^29^.

Physics-based Algorithms are inspired by fundamental laws of physics and chemistry^30^. Classical representatives include Simulated Annealing (SA)^31^, Gravitational Search Algorithm (GSA)^32^. Newly developed variants in this category comprise the Artificial Electric Field Algorithm(AEFA)^33^, Elastic Deformation Optimization Algorithm(EDOA)^34^, Chernobyl Disaster Optimizer(CDO)^35^, Bermuda Triangle Optimizer (BTO)^36^.

A summary of representative metaheuristic algorithms published in recent years is provided in Table 1.

**Table 1:** Some of the basic optimization algorithms.

Basic algorithm	Category name	Strengths	Weaknesses	Year
Genetic Algorithm (GA)	Evolutionary Algorithms	easy to implement, Strong search capability, Naturally parallelizable	Relatively slow convergence, Prone to local optima, Highly sensitive to parameter settings	1992
**Differential Evolution(DE)**	Evolutionary Algorithms	Easy to implement, Fast convergence rate, Few control parameters, Strong search capability	Sensitive to parameter tuning, Prone to premature convergence, Susceptible to search stagnation	1997
Ant Lion Optimizer(ALO)	Evolutionary Algorithms	Elite preservation strategy, Few parameters, strong robustness	Prone to local optima, Limited global exploration capability, Convergence speed highly, dependent on initial population quality, Reduced computational efficiency in high-dimensional problems	2015
Plant Competition Optimization (PCO)	Evolutionary Algorithms	Strong search capability	numerous control parameters, high sensitivity to parameter tuning, and relatively high computational cost.	2022
**Particle Swarm Optimization (PSO)**	Swarm Intelligence	Simple implementation, fast convergence, fewer control parameters, and strong global search capability	prone to local optima, sensitive to parameter settings, and exhibits insufficient accuracy and stability‌	1995
Sperm Swarm Optimization (SSO)	Swarm Intelligence	Strong global and distributed search capabilities	parameter sensitivity and relatively slow convergence	2018
**Starling Murmuration Optimizer (SMO)**	Swarm Intelligence	Powerful search ability, rapid convergence, and innovative search strategies	employs static strategy switching without dynamic adaptation, incurs high computational complexity, and pron to local optima.	2022
Golden Jackal Optimization (GJO)	Swarm Intelligence	Easy to implement, few control parameters and strong early-stage global exploration	tends toward excessive exploitation, prone to local optima	2022
**Dwarf Mongoose Optimization (DMO)**	Swarm Intelligence	Strong global exploration, good stability, minimal control parameters, and simple structure	exhibits slow convergence, vulnerability to local optima, and sensitivity to parameter configuration.	2022
**Dung Beetle optimizer(DBO)**	Swarm Intelligence	Compact parameterization, easy to implement and interpret; hierarchical search mechanism	shows limited adaptability across population sizes and prone to loss of population diversity	2023
**Black-winged Kite Algorithm(BKA)**	Swarm Intelligence	Simple structure, adaptive perturbation adjustment, dual behavior modes	insufficient convergence stability, inadequate global optimum guidance, and high sensitivity to parameter settings	2024
**Draco lizard optimizer(DLO)**	Swarm Intelligence	Stage-wise search strategy, adaptive perturbation mechanism, simple structure	over-reliance on global optima, excessive randomness during exploration, and rigid stage division	2025
Artificial Protozoa Optimizer (APO)	Swarm Intelligence	Multi-behavior mode synergy, adaptive parameter tuning, efficient utilization of neighborhood information	high computational complexity, insufficient convergence stability, and difficulty in fine-tuning parameters	2025
Imperialist Competitive Algorithm (ICA)	Human-based	high computational complexity, insufficient convergence stability, and difficulty in fine-tuning parameters	insufficient diversity in later stages, high parameter sensitivity, convergence speed limited by empire merging efficiency, and potential stagnation in later phases	2007
Cooperation search algorithm(CAS)	Human-based	Strong optimization capability, fast convergence	high computational overhead and prone to premature	2021
Five Phases Algorithm (FPA)	Human-based	Differentiated search, dual-mode updates balance global exploration and local exploitation, simple and easy to implement	slow convergence, prone to local optima, and sensitive to parameter settings	2023
**Hiking Optimization Algorithm**	Human-based	Simple structure, few parameters, dynamic step-size adjustment balances exploration and exploitation, strong local search orientation	simplistic random design leads to single search direction, slow convergence, and tendency to get trapped in local optima	2024
Simulated Annealing (SA)	Physics-based	Effective at escaping local optima in early stages, adaptive cooling and perturbation decay balance exploration and exploitation, parallel population search improves efficiency and robustness, simple implementation with few parameters	slow convergence, sensitive to parameter tuning, weak adaptability to high-dimensional multimodal problems, and risk of stagnation in local optima	1993
Gravitational Search Algorithm (GSA)	Physics-based	Multiple sampling methods, simple structure, suitable for complex models	high parameter sensitivity, high computational time, and reliance on prior parameter distributions	2009
Water wave optimization	Physics-based	Simple framework, good balance between global and local search	slow convergence and lower computational accuracy	2015
Chernobyl Disaster Optimizer (CDO)	Physics-based	Three-leader parallel contraction, balanced exploration and exploitation, simple and easy to implement	prone to local optima in high-complexity problems, sensitive to parameters, and lack of diversity maintenance.	2023
Bermuda Triangle Optimizer (BTO)	Physics-based	prone to local optima in high-complexity problems, sensitive to parameters, and lack of diversity maintenance.	late-stage diversity loss leads to local optima, difficult parameter tuning, and poor stability in high-complexity optimization problems	2025

Meanwhile, researchers have refined various algorithms, continually generating a range of algorithmic variants.For instance, Zhang et al. proposed an improved COA by integrating chaotic sequences, a nonlinear inertia weight, and an adaptive t-distribution mutation strategy. Its application to typical engineering problems demonstrated significant improvements in convergence speed and optimization accuracy, along with excellent robustness. Similarly, Hu et al. developed an improved Dung Beetle Optimization (DBO) algorithm by fusing cubic chaos mapping, a cooperative search algorithm, and both t-distribution and differential evolution mutation strategies. This hybrid approach resulted in enhanced robustness and superior optimization capabilities. More recently, Yu et al. enhanced the Sand Cat Swarm Optimization algorithm using chaos mapping, nonlinear control, a sparrow alert mechanism, and Gaussian-Cauchy mutation. This improved its global exploration and local exploitation abilities, and its application to WSN ranging and localization yielded faster convergence and higher positioning accuracy^37^. Qian’s team enhanced the SCSO algorithm through escape mechanisms, elite collaboration, and differential perturbation, validating its effectiveness in 3D sensor deployment and drone path planning as a robust optimizer^38^. Ding and colleagues introduced an enhanced WOA (Whale Optimization Algorithm) by incorporating chaos-based initialization, a nonlinear convergence factor, and chaotic inertia weight to strengthen its global search capabilities^39^. Liu introduced an enhanced Sparrow Search Algorithm (SSA) incorporating Circle chaotic mapping and T-distribution mutation, improving both global optimization performance and convergence accuracy^40^. Cao et al. enhanced the moth algorithm by integrating an adaptive crossover operator, Lévy flight strategy, and adaptive t-distribution mutation into the flight straight strategy, while employing a greedy selection mechanism to boost global search efficiency and convergence speed^41^. Mohammad’s team enhanced the Cuckoo Search Algorithm (CSA) by hybridizing it with the Bat Algorithm (BA), addressing the standard CSA’s slow convergence and tendency to stagnate in local optima^42^. Khalilpourazari and colleagues developed a fusion algorithm that demonstrated superior local development, along with improved precision and robustness by combining the sine-cosine algorithm and crow search algorithm^43^. Mohammad and co-researchers augmented MFO’s performance by combining it with Hill Climbing (MFOHC) for superior exploitation, and adopted proportional selection to ensure solution diversity and effective exploration^44^. Garg’s research team introduced an enhanced Biogeography-Based Optimization (BBO) algorithm designed to reduce parameter control complexity. This improved version integrates the exploration capabilities of the Salp Swarm Algorithm (SSA) with the exploitation mechanisms of BBO, thereby achieving robust optimization performance^45^.

The parrot Optimizer (PO) is a novel metaheuristic algorithm inspired by the adaptive behaviors of the Pyrrhura Molinae parrots, created by Lian, GB in 2024^46^. Studies have found that PO is a promising and competitive algorithm, outperforming some existing ones. Unlike the conventional two-phase exploration-exploitation framework, the parrot Optimizer (PO) is designed to effectively escape local optima by randomly assigning one of four distinct behaviors to each agent in the population^47^. Therefore, due to its simple structure and fewer control parameters, PO has been widely used.Nevertheless, in accordance with the “No Free Lunch” (NFL) theory^10^ PO shares the limitation of all metaheuristic algorithms in that it is not a panacea for all complex real-world optimization problems. A critical analysis of PO’s operational mechanics reveals several inherent shortcomings. First, its reliance on uniform random initialization can result in a non-uniform distribution of the initial population, which compromises its early global exploration capacity, impedes convergence velocity, and increases its susceptibility to premature convergence^48^. Second, the “global best guidance + Lévy flight” strategy within the staying behavior phase lacks a risk-perception mechanism. Consequently, when guiding inferior individuals, it is prone to entrapment in local optima, leading to sluggish convergence and excessive population clustering. Third, the communicating behavior is governed by a stochastic condition, rendering the algorithm’s trajectory unpredictable and difficult to reproduce. This can curtail a comprehensive search of the solution space and precipitate premature convergence, particularly in complex or high-dimensional problems^47^. Fourth, the “fear of strangers” behavior employs a uniform cosine perturbation for all individuals, failing to differentiate between high- and low-quality solutions and lacking a robust strategy to move away from the worst-performing individuals. This indiscriminate approach leads to computationally expensive, large-step perturbations by inferior agents that can paradoxically hasten premature convergence. Finally, the absence of an effective restart mechanism makes PO vulnerable to stagnation in a “plateau phase,” where a lack of improvement in the best-found solution over successive iterations leads to wasted computational effort.

In this paper, We proposes an enhanced version of the parrot Optimizer (PO), named the Enhanced parrot Optimizer (EPO). By synergistically integrating chaotic sequences, nonlinear decay factors, t-distribution mutation, risk-aware early-warning mechanisms, and differential evolution, among other strategies, the performance of the parrot Optimizer is improved. The introduction of chaotic sequences aims to increase population diversity and reduce the risk of premature convergence; the employment of a Sparrow Search Algorithm (SSA)–based early-warning mechanism helps prevent the algorithm from becoming trapped in local optima; the nonlinear decay factor is introduced to coordinate the dynamic balance between global exploration and local exploitation; the incorporation of a hybrid “t-distribution + differential evolution” local restart module further enhances population diversity and strengthens the algorithm’s ability to escape local optima.The main contributions of this paper are as follows:

- We put forward an Enhanced PO algorithm (EPO) which incorporates a synergistic amalgamation of multiple strategies, such as chaotic sequences, a nonlinear decay factor, an alert update mechanism, a t-distribution mutation strategy, and Differential Evolution. This algorithm attains substantial performance enhancements via strategic innovations throughout its four crucial phases.
- The optimization performance of the proposed EPO algorithm is rigorously assessed through the utilization of the CEC2017 benchmark test functions. Moreover, the proposed EPO algorithm is comprehensively contrasted with eleven state-of-the-art metaheuristic algorithms across multiple dimensionalities (Dim = 30, 50, 100). These algorithms encompass: Multi-strategy Improved parrot Optimization (MPO)^49^, parrot Optimizer (PO)^46^, Starling Murmuration Optimizer (SMO)^7^, Bermuda Triangle Optimizer (BTO)^36^, Hiking Optimization Algorithm (HOA)^29^, Harris hawks optimization(HHO)^50^, Dung Beetle Optimizer (DBO)^20^, Black-winged kite algorithm(BKA)^21^, and Ivy algorithm^51^.
- The practical applicability of EPO is manifested through its successful implementation in the Wireless Sensor Network (WSN) node deployment problem, which validates its efficacy in tackling real-world engineering optimization challenges.

**The subsequent sections of this paper are structured as follows**: Section 1 presents an introduction to the research background and motivation, encompasses a concise literature review, and culminates in a summary of the paper’s principal contributions. Section 2 offers a comprehensive introduction to the mathematical model of the standard parrot Optimizer (PO). Section 3 systematically expounds on the proposed improvement strategies, including the core modifications such as chaotic initialization, dynamic behavior optimization, and the hybrid perturbation mechanism. Section 4 addresses the methodology, results, and analysis of the global optimization experiments. Section 5 applies the EPO algorithm to the practical engineering problem of Wireless Sensor Network (WSN) node deployment. Finally, Sect. 6 concludes the paper with a summary and identifies potential directions for future research.

### Related works

Wireless sensor networks (WSNs) find extensive applications in diverse domains, including medicine and engineering. However, challenges persist in deployment strategies and node positioning, which have an impact on crucial performance indicators, such as regional coverage^52^. The deployment strategy is one of the fundamental determinants of WSNs’ performance, directly influencing key indicators like coverage. To achieve this objective, scholars utilize meta-heuristic algorithms to tackle the challenges associated with deployment strategies. They optimize the coverage of wireless sensor networks by minimizing node redundancy and improving coverage^53^. Hanh et al. integrated the Laplacian crossover and arithmetic crossover operators to put forward a wireless sensor network (WSN) coverage optimization approach in the form of a genetic algorithm, which effectively enhanced the network coverage^54^. Li et al. put forward an enhanced multi-objective ant lion optimization algorithm founded on fast non-dominated sorting. This algorithm effectively broadened the coverage scope of wireless sensor networks and diminished the average distance of node movement, targeting the two objectives of wireless sensor coverage and average node movement distance^55^. Cao et al. put forward a wireless sensor network (WSN) coverage optimization approach grounded in chaotic-improved social spider optimization, with the objective of minimizing energy consumption and enhancing WSN coverage^56^. Yang et al. put forward an improved cuckoo search algorithm (ICS-MS) featuring multiple strategies, which enhances coverage and simultaneously reduces deployment costs^57^. Shaikh et al. proposed an Enhanced Chaotic Grey Wolf Optimization (ECGWO) algorithm to enhance wireless sensor network (WSN) coverage and connectivity, while tackling challenges such as high deployment costs, limited coverage, and connectivity gaps. The results indicate significant improvements in coverage and connectivity, making it a dependable solution for deployment challenges across various scenarios^58^. Wang and colleagues proposed an enhanced Salamander Swarm Algorithm (SSA) to efficiently solve coverage optimization challenges^59^. Amer et al. proposed the CFL-PSO method to enhance connectivity and coverage in wireless sensor networks^60^. Zeng et al. proposed an enhanced WHO method incorporating adversarial learning and the modified Caccioli variation strategy, effectively addressing coverage and connectivity challenges in heterogeneous wireless sensor networks^61^. Cao et al. proposed an enhanced SOA algorithm based on PSO, which effectively improves network coverage while reducing redundancy and blind spots^62^. Nematzadeh et al. proposed an enhanced GWO algorithm that maximizes resource utilization by reducing the number of nodes while maintaining optimal coverage and connectivity^63^. Dinesh et al. proposed a neural fuzzy approach based on sparrow search optimization for wireless sensor networks, which enhances connectivity and coverage^64^. Wang et al. proposed a novel Bighorn sheep optimization algorithm (BSOA) inspired by the social behavior of antelopesand applied it to WSN deployment and other complex optimization problems.Inspired by the social behavior of antelopes^65^. Wang et al. proposed a novel algorithm called Cape Lynx Optimizer (CLO) inspired by the behavioral patterns of Cape Lynx,, which has been applied to WSN deployment and other complex optimization problems^66^.

The aforementioned approaches have made significant contributions to WSN coverage optimization. However, existing algorithms for optimizing WSN coverage face certain limitations. These methods struggle with premature convergence and local optima, which restrict their performance across different scenarios. While various optimization techniques effectively balance global exploration, they may struggle to maximize coverage and connectivity in diverse practical situations. Therefore, improving existing novel algorithms remains an effective approach to address these challenges^58^.

## Mathematical model for parrot optimizer

parrot Optimizer(PO) is a novel metaheuristic algorithm inspiring from adaptive behaviors exhibited of the Pyrrhura Molinae parrots created by Lian, GB in 2024^46^.The steps for parrot Optimizer(PO) are introduced in the following subsection.

### Population initialization

The initialization phase of the parrot Optimizer (PO) algorithm is governed by several key parameters: the population size (N), the maximum iteration count (Max_iter), and the feasible search domain, which is constrained by lower ($lb$) and upper ($ub$) bounds. The initial placement of each individual within this domain is determined by the Eq. (1)^67^:

1 $$X_{i}^{0}=lb+rand(0,1) \cdot \left( {ub - lb} \right)i=1,2, \cdots ,N,j=1,2, \cdots ,m$$

In Eq. (1), $rand(0,1)$denotes a random value within the interval [0,1], and$X_{i}^{0}$ represents the initial position of the$ith$ parrot.

### Foraging behavior

As a component of its foraging behavior, after estimating the approximate location of a food source, a parrot primarily flies toward it by either observing the food source’s position or considering the host’s position. This movement is mathematically modeled by the Eq. (2)^68^:

2 $$X_{i}^{{t+1}}=\left( {X_{i}^{t} - {X_{best}}} \right) \times levy+rand(0,1) \times {(1 - \frac{t}{T})^{\frac{{2t}}{T}}} \times X_{{mean}}^{t}$$

In Eq. (2),$X_{i}^{t}$ denotes the current position of the parrot, while $X_{i}^{{t+1}}$ represents its updated position in the next iteration.$levy\left( {\dim } \right)$is drawn from a Lévy distribution, which is used to model the flight characteristics of parrots. $X_{{mean}}^{t}$ signifies the mean position of the current population. ${X_{best}}$represents the best solution found so far (the global best position), which also corresponds to the location of the host. The symbol *t* denotes the current iteration number. The term $rand(0,1) \times {(1 - {t \mathord{\left/ {\vphantom {t T}} \right. \kern-0pt} T})^{{{2t} \mathord{\left/ {\vphantom {{2t} T}} \right. \kern-0pt} T}}} \times X_{{mean}}^{t}$ models the process of monitoring the overall population to refine the direction toward the food source. Conversely, the term $\left( {X_{i}^{t} - {X_{best}}} \right) \times levy\left( {\dim } \right)$ models the movement based on the parrot’s position relative to the host (${X_{best}}$).

The mean position of the current population, $X_{{mean}}^{t}$, is determined using the procedure in Eq. (3):

3 $$X_{{mean}}^{t}=\frac{1}{N}\sum\limits_{{k=1}}^{N} {X_{k}^{t}}$$

Using the formula from(2), where the value of γ is 1.5, the Lévy distribution can be derived by usign the Eq. (4):

4 $${\text{Levy}}(dim)=\frac{{\mu \cdot \sigma }}{{|\nu {|^{\frac{1}{\gamma }}}}}$$

In Eq. (4), $$\mu \sim \mathcal{N}(0,dim)$$, $$\nu \sim \mathcal{N}(0,dim)$$, $$\sigma ={\left( {{{\Gamma (1+\gamma ) \cdot \sin \left( {\frac{{\pi \gamma }}{2}} \right)} \mathord{\left/ {\vphantom {{\Gamma (1+\gamma ) \cdot \sin \left( {\frac{{\pi \gamma }}{2}} \right)} {\Gamma \left( {\frac{{1+\gamma }}{2}} \right) \cdot \gamma \cdot {2^{\frac{{1+\gamma }}{2}}}}}} \right. \kern-0pt} {\Gamma \left( {\frac{{1+\gamma }}{2}} \right) \cdot \gamma \cdot {2^{\frac{{1+\gamma }}{2}}}}}} \right)^{\gamma +1}}$$

### Staying behavior

The staying behavior of social parrots primarily involves suddenly flying toward any part of the host’s body and then remaining stationary for a predetermined period. This process is modeled by usign the Eq. (5)^47^:

5 $$X_{i}^{{t+1}}=X_{i}^{t}+{X_{best}} \times levy+rand(0,1) \times ones\left( {1,\dim } \right)$$

In Eq. (5), $ones\left( {1,\dim } \right)$denotes a vector of ones with dimension dim. The term $rand(0,1) \times ones\left( {1,\dim } \right)$models the random perching on a part of the host’s body, while the term ${X_{best}} \times levy\left( {\dim } \right)$ models the flight towards the host.

### Communicating behavior

The communicating behavior of Pyrrhura Molinae parrots can be divided into two types: flying to the flock and without flying to the flock. Considering that both cases have the same probability of happening, these two types of communication behaviors can be represented in Eq. (6)^68^:

6 $$X_{i}^{{t+1}}=\left\{ \begin{gathered} 0.2 \times rand(0,1) \times (1 - \frac{i}{T}) \times \left( {X_{i}^{t} - X_{{mean}}^{t}} \right),H \leqslant 0.5 \hfill \\ 0.2 \times rand(0,1) \times \exp ( - \frac{i}{{rand(0,1)T}}),H>0.5 \hfill \\ \end{gathered} \right.$$

In this expression, *H* represents a random probability drawn uniformly from the interval [0, 1]. The first conditional branch emulates the behavior of individuals assimilating into a group, whereas the second branch models the scenario of an immediate departure following an interaction.

### Fear of strangers’ behavior

Birds typically exhibit an innate distrust of strangers, and parrots are no exception. The Eq. (7) models their behavior when seeking a safe environment, avoiding strangers, and seeking refuge with their host^69^:

7 $$X_{i}^{{t+1}}=X_{i}^{t}+rand(0,1) \times cos(0.5\pi \times \frac{t}{T}) \times \left( {{X_{best}} - X_{i}^{t}} \right) - \cos (rand(0,1) \times \pi ) \times {(\frac{t}{T})^{\frac{2}{T}}} \times \left( {X_{i}^{t} - {X_{best}}} \right)$$

In this equation, the term $\cos (rand(0,1) \times \pi ) \times {({t \mathord{\left/ {\vphantom {t T}} \right. \kern-0pt} T})^{{2 \mathord{\left/ {\vphantom {2 T}} \right. \kern-0pt} T}}} \times \left( {X_{i}^{t} - {X_{best}}} \right)$ models the process of reorienting and flying toward the host.The term $\cos (rand(0,1) \times \pi ) \times {({t \mathord{\left/ {\vphantom {t T}} \right. \kern-0pt} T})^{{2 \mathord{\left/ {\vphantom {2 T}} \right. \kern-0pt} T}}} \times \left( {X_{i}^{t} - {X_{best}}} \right)$ models the process of separating from a stranger.

## Mathematical model for enhanced parrot optimizer

### Population initialization

The original parrot Optimizer (PO) algorithm initializes parrot positions using a uniform random method, which often leads to a non-uniform distribution of the initial population within the search space, making it prone to forming “voids” or local aggregations. This non-uniformity weakens the algorithm’s global exploration capability in the early stages and causes its subsequent behavioral phases to become prematurely trapped in local attractors, resulting in slowed convergence and a high risk of premature convergence. To address these issues, a Cubic map chaotic sequence mechanism is introduced for position initialization. This approach ensures that the initial solutions are more uniformly distributed, generates higher-quality starting points, increases population diversity, and consequently reduces the risk of premature convergence.

8 $${z_i}={x_{n+1}}=\alpha {x_n}(1 - x_{n}^{2})$$

9 $$X_{i}^{0}=lb+{z_i} \cdot \left( {ub - lb} \right)$$

### Staying behavior

The update strategy in the original algorithm is based on a “global best-guided + Lévy flight” approach. This, however, causes inferior individuals far from the optimum to continue undergoing large-scale random perturbations, which slows down convergence. Simultaneously, the lack of a “danger perception” mechanism makes the population prone to excessive aggregation around local optima, leading to entrapment in local solutions.To address this, we draw inspiration from the sparrow’s vigilance mechanism—the concept of “immediately contracting toward a safe area upon detecting danger.” Accordingly, a vigilance mechanism is introduced, which is triggered when an individual’s fitness value is worse than the global best fitness. The corresponding mathematical expression is in eqution (10):

10 $${\mathbf{X}}_{j}^{{t+1}}=\left\{ {\begin{array}{*{20}{l}} {{\mathbf{X}}_{{{\text{best}}}}^{t}+\beta \odot |{\mathbf{X}}_{j}^{t} - {\mathbf{X}}_{{{\text{best}}}}^{t}|,}&{f_{j}^{t}>f_{{{\text{best}}}}^{t}} \\ {{\mathbf{X}}_{j}^{t}+{\mathbf{X}}_{{{\text{LBest}}}}^{{(g(j))}} \odot L{\text{evy}}+\beta (1 - t/T),}&{f_{j}^{t} \leqslant f_{{{\text{best}}}}^{t}} \end{array}} \right.$$

In this equation,${\mathbf{X}}_{{{\text{best}}}}^{t}$: The current global best position.$\beta$: A step-size control parameter drawn from a standard normal distribution (mean = 0, variance = 1).$f_{j}^{t}$: The fitness value of the current parrot individual.$f_{{{\text{best}}}}^{t}$: The current global best and worst fitness values.

### Communicating behavior

In the original algorithm, the update for the “Communicating behavior” is contingent upon a randomly generated value (*H*). This stochastic trigger causes the algorithm’s results to vary between runs, making its behavior difficult to predict and reproduce. It can also lead to premature convergence, an issue that is particularly pronounced in large or complex search spaces where the algorithm may fail to explore the solution space sufficiently.To better balance global exploration and local exploitation—enabling broader search in the early iterations and more refined search in the later stages—this paper introduces a method for generating a non-linear decreasing factor. The mathematical expression is as follows:

11 $$p=1 - \sqrt {\frac{{{e^{t/T}} - 1}}{{e - 1}}} ,H=(1 - t/T)p$$

12 $${\mathbf{X}}_{j}^{{t+1}}=\left\{ {\begin{array}{*{20}{l}} {{\mathbf{X}}_{j}^{t}+\alpha (1 - t/T)({\mathbf{X}}_{j}^{t} - {{\overline {{\mathbf{X}}} }_j}),}&{H>0.5} \\ {{\mathbf{X}}_{j}^{t}+\alpha (1 - t/T)\exp \left( { - \frac{j}{{rT}}} \right),}&{{\text{otherwise}}} \end{array}} \right.$$

13 $$\alpha \sim U(0,0.2),r\sim U(0,1)$$

### Fear of strangers’ behavior

In the “scared of strangers” phase of the original PO algorithm, a random cosine perturbation is uniformly applied to all individuals. This approach neither differentiates individuals based on their fitness nor includes a strategy to move away from the worst-performing individual. This causes inferior individuals to execute large-scale jumps far from the optimal region, which wastes function evaluations and can easily trigger premature convergence.To address this, this paper proposes a two-layer decision mechanism.Outer Layer: A vigilance-based pullback is first triggered if an individual’s fitness is inferior to the global best, rapidly guiding these suboptimal solutions back toward the main population.Inner Layer: For the remaining superior individuals, a 50% probability is used to select one of two actions: either moving away from the current worst individual or applying a fine-grained cosine perturbation, thereby maintaining population diversity.

14 $${\mathbf{X}}_{j}^{{t+1}}=\left\{ {\begin{array}{*{20}{l}} {{\mathbf{X}}_{{{\text{best}}}}^{t}+r \odot \left| {{X_j} - {\mathbf{X}}_{{{\text{best}}}}^{t}} \right|,}&{{\text{if }}f_{j}^{t}>f_{{{\text{best}}}}^{t},} \\ {{\mathbf{X}}_{j}^{t}+\left\{ {\begin{array}{*{20}{l}} {(2{\mkern 1mu} r - 1) \cdot \frac{{\left| {{\mathbf{X}}_{j}^{t} - {\mathbf{X}}_{{{\text{worst}}}}^{t}} \right|}}{{f_{j}^{t} - f_{{{\text{worst}}}}^{t}+\varepsilon }},}&{{\text{with probability }}0.5,} \\ {r\cos \left( {\frac{{\pi t}}{{2T}}} \right)({\mathbf{X}}_{{{\text{best}}}}^{t} - {\mathbf{X}}_{j}^{t}) - \cos (\theta ){{\left( {\frac{t}{T}} \right)}^{2/T}}({\mathbf{X}}_{j}^{t} - {\mathbf{X}}_{{{\text{best}}}}^{t}),}&{{\text{otherwise}},} \end{array}} \right.}&{{\text{otherwise}}.} \end{array}} \right.$$

In this equation, $r,\theta \sim U(0,1)$, $f_{j}^{t}$ denotes the fitness value of the current parrot individual, $f_{{{\text{best}}}}^{t}$ represents the current worst fitness value, and $\varepsilon$ is a constant that prevents the denominator from becoming zero. The model accounts for two scenarios. The first scenario ($f_{j}^{t}>f_{{{\text{best}}}}^{t}$) describes a parrot positioned at the edge of the population, which is more susceptible to predator attacks. The second scenario occurs when a specific condition ($f_{j}^{t}=f_{{{\text{best}}}}^{t}$) is met, indicating that a parrot within the group perceives a threat and must move closer to its peers for protection against predation.

### Stagnation handling and population diversity enhancement strategy

During the late stages of the PO algorithm’s iterative process, population diversity naturally decreases, causing individuals to become homogenous. This makes the algorithm highly susceptible to entrapment in local optima and leads to premature convergence, a phenomenon where the global best solution fails to improve over multiple consecutive iterations, causing the search to stagnate. To effectively address this critical issue, this paper proposes a “t-distribution perturbation + differential evolution” local restart module. This module is triggered when the algorithm detects no improvement for three consecutive generations.It operates as follows:

For individuals with fitness above the population average, the heavy-tailed property of the t-distribution is leveraged to implement a large-scale jump, with the goal of escaping the local basin of attraction.

For individuals with fitness below the average, a differential evolution perturbation is executed, utilizing difference vectors to conduct a directional, fine-grained search.

This strategy aims to increase the diversity of the sub-population with lower fitness values and enhance the algorithm’s ability to escape local optima.

#### Stagnation detection mechanism

This paper introduces a simple stagnation detector. By tracking the global best fitness value in each iteration, the detector determines whether the algorithm has stagnated. The specific condition is as follows:

15 $$Stagnation=\left\{ {\begin{array}{*{20}{c}} {True,if\;\;iter>k~,and~f_{{best}}^{{\left( {iter - k} \right)}}=f_{{best}}^{{\left( {iter - 1} \right)}}} \\ {False,otherwise} \end{array}} \right.$$

In this equation,$f_{{best}}^{{(iter)}}$is the global best fitness value at iteration $iter$. In the implementation for this paper, the stagnation window *k* is set to 3. That is, the stagnation handling mechanism is triggered when the global best solution fails to improve for three consecutive iterations.

#### Hybrid mutation strategy design

Once stagnation is detected, the algorithm executes a special hybrid mutation operation on the current population. The core principle of this strategy is “divide and conquer”: applying different mutation operators to individuals in different states to achieve efficient perturbation and exploration. Specifically, it combines two powerful mutation strategies: t-distribution perturbation and Differential Evolution (DE).

## t-distribution perturbation for inferior individuals

Individuals in the population with poor fitness values (i.e., greater than the average fitness value) are likely located in “barren” regions far from the global optimum. Applying a large-scale positional perturbation to these individuals helps relocate them to more promising new areas of the search space. To achieve this, this paper employs the Student’s t-distribution for perturbation.The t-distribution is known for its heavy-tailed property. Compared to the Gaussian distribution, it has a higher probability of generating random numbers far from its mean. This characteristic makes it highly suitable as a powerful source of perturbation for escaping local optima. Its update formula is as follows:

16 $$X_{i}^{{t+1}}={X_{{\text{best}}}}+{X_{{\text{best}}}}T(i)$$

In this equation,$X_{i}^{t}$is the position of a suboptimal individual (i.e.,${f_i}>{f_{{\text{mean}}}}$,one whose fitness is above the population average), ${X_{{\text{best}}}}$ is the current global best position. The perturbation is driven by $T(i)$, a random vector drawn from a Student’s t-distribution whose degrees of freedom are dynamically set by the current iteration count *i*.This design creates an elegant self-adaptive mechanism. During the initial exploratory phase, the low iteration count results in a t-distribution with pronounced heavy tails, generating large-scale perturbations capable of launching the individual out of local optima. Conversely, as the algorithm progresses and the iteration count increases, the t-distribution progressively approximates a Gaussian distribution. This naturally tempers the perturbation strength, facilitating a transition from broad exploration to fine-grained local refinement. This adaptive behavior is crucial for balancing the search process effectively.

## Differential evolution for superior individuals

Conversely, for individuals in the population with better fitness values (i.e., ${f_i}<{f_{{\text{mean}}}}$, where fitness is less than the average), they are typically located in promising regions. Applying drastic random perturbations to these individuals could destroy valuable information that has already been discovered. Therefore, this paper employs the Differential Evolution (DE) mutation strategy to guide them in a more directional exploration.Differential Evolution generates new candidate solutions by utilizing difference vectors among individuals in the population, which is an effective approach that leverages the collective intelligence of the population for efficient search. Its mutation formula is as follows:

17 $$X_{i}^{{t+1}}=X_{{r,1}}^{t}+F\left( {X_{{r,2}}^{t} - X_{{r,3}}^{t}} \right)$$

In this equation,$X_{{r,1}}^{t}$,$X_{{r,2}}^{t}$, and $X_{{r,3}}^{t}$ are three vectors randomly sampled from the current population, under the constraint that they are mutually distinct and different from the target individual $X_{i}^{t}$. The parameter *F* is the scaling factor, which modulates the intensity of the differential perturbation and is fixed at 0.15 in our study. This strategy serves the dual purpose of effectively sustaining population diversity and guiding a targeted search for new candidate solutions within the current high-potential zones of the search space.

### Pseudocode and flowchart

The pseudo-code for the Enhanced PO (EPO) algorithm, which synergistically integrates multiple strategies including chaotic sequences, a non-linear decay factor, t-distribution mutation, a vigilance update mechanism, and differential evolution, is presented below.

**Fig. 1:**
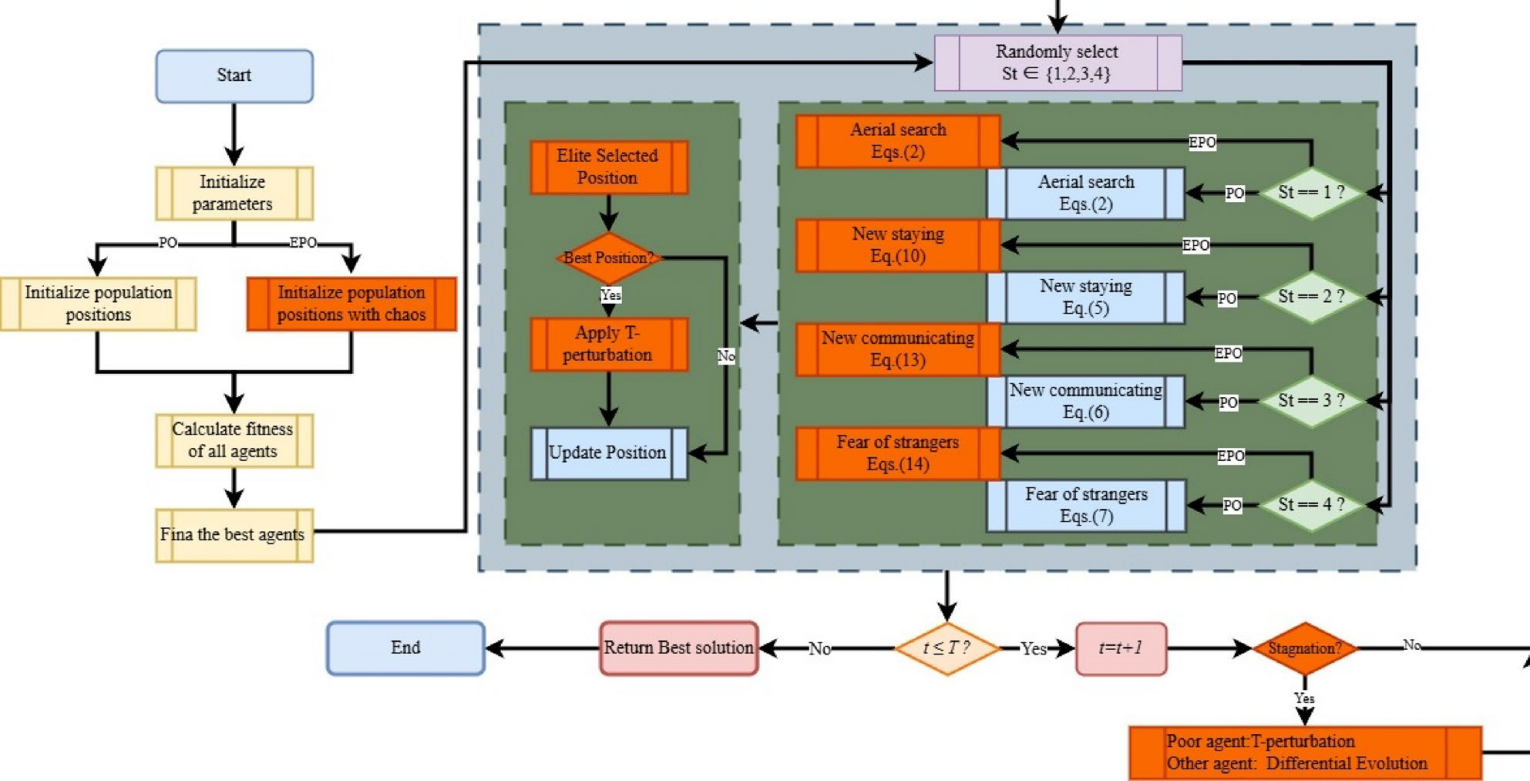
Flowchart for the Enhanced PO (EPO) algorithm.

The flowchart for the Enhanced PO (EPO) algorithm is shown in Fig. 1.

### Computational complexity

To comprehensively assess the computational efficiency of the proposed Enhanced parrot Optimizer (EPO), we conducted a systematic analysis of its computational complexity. The analysis is based on three core parameters: the population size N, the problem dimension D, and the maximum number of iterations T.First, during the initialization phase, EPO employs a Cubic chaotic map to generate the initial population. This process, which involves generating initial values for D dimensions across N individuals, has a complexity of $O(N \times D)$. This is consistent with the complexity of standard random initialization methods.Within a single iteration of the main loop, the computational overhead originates from several core components. These include the cost of calculating the fitness for N individuals $O(N)$, assuming fitness evaluation is dimension-dependent) and the sorting operation required to find the global best solution and support other potential ranking-dependent mechanisms, which has a complexity of $O(N\;\log \;N)$. Furthermore, the core operation of updating the entire population’s positions has a complexity of $O(N \times D)$. The SSA vigilance mechanism and the improved non-linear factor we introduced guide the search direction through conditional checks and a small number of arithmetic operations. These operations have a computational cost of $O(1)$and therefore do not alter the fundamental order of complexity of the position update step.Critically, the newly added stagnation handling mechanism is designed to escape local optima. When triggered, it requires iterating through the population and applying either the t-distribution or DE strategy based on fitness conditions. This process introduces an additional computational overhead of $O(N)$, accompanied by the cost of fitness re-evaluation, which is also $O(N \times D)$.Aggregating the complexity of all components, it is evident that the additional overhead from the stagnation handling mechanism is on the same order of magnitude as the position update step. According to the rules of Big O notation, these supplementary computational costs are theoretically absorbed by the existing dominant terms. Consequently, the total computational complexity of the EPO algorithm over T iterations is $O(N\;\log \;N+N \times D)$, which is identical to the theoretical complexity of the original PO algorithm^46^.The final conclusion is that the proposed EPO algorithm significantly enhances global search capability and convergence precision without increasing its theoretical computational complexity. Although its practical runtime per iteration may be slightly higher than that of the native algorithm due to the execution of more sophisticated search strategies, this overhead is justified and efficient in light of the substantial performance gains.

### Ablation study(CEC2017, D = 30)

**Fig. 2:**
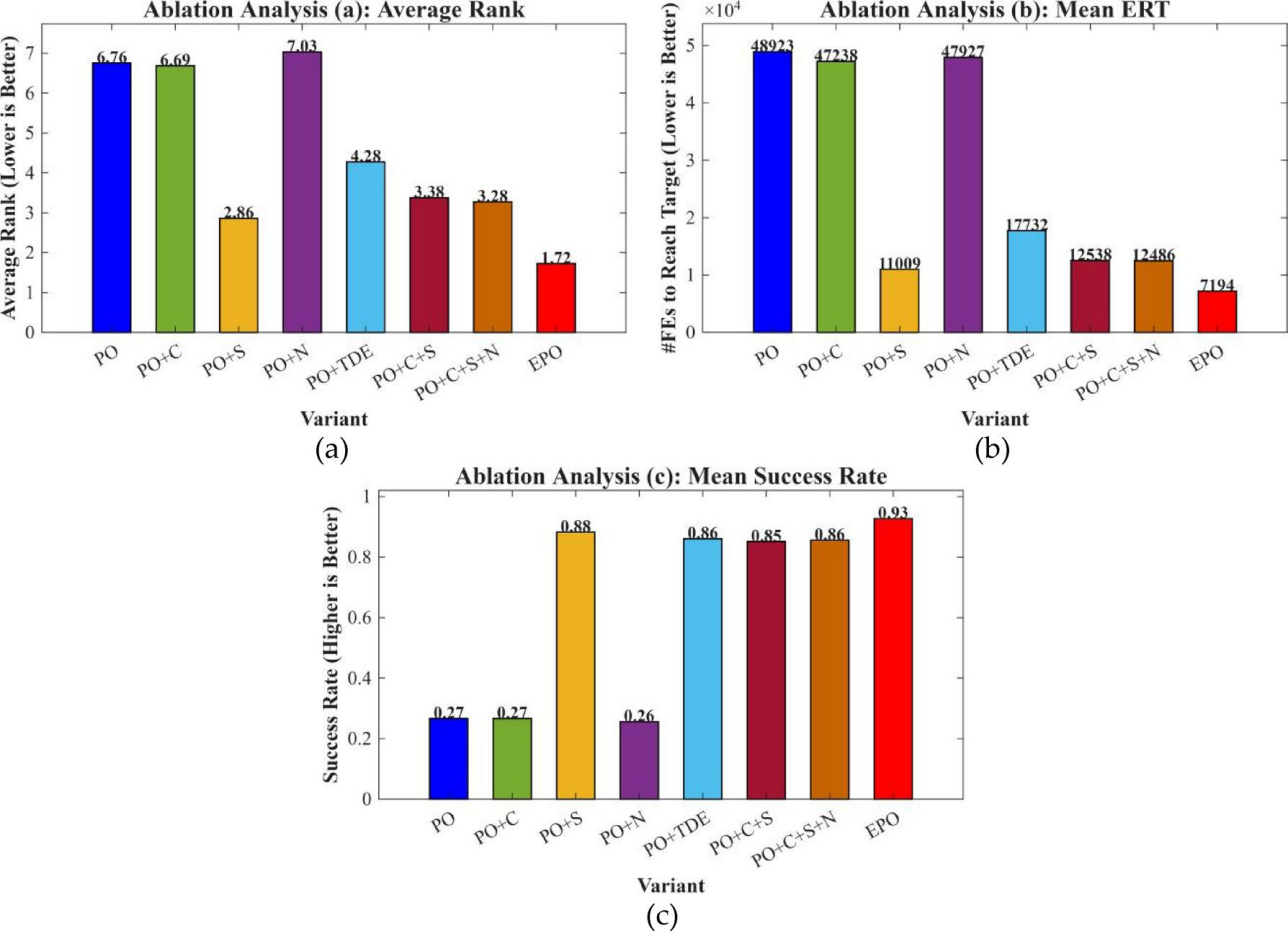
Main effects analysis. (**a**) Median effects, (**b**) Mean expected running time in iterations, (**c**) Mean success rate.

**Fig. 3:**
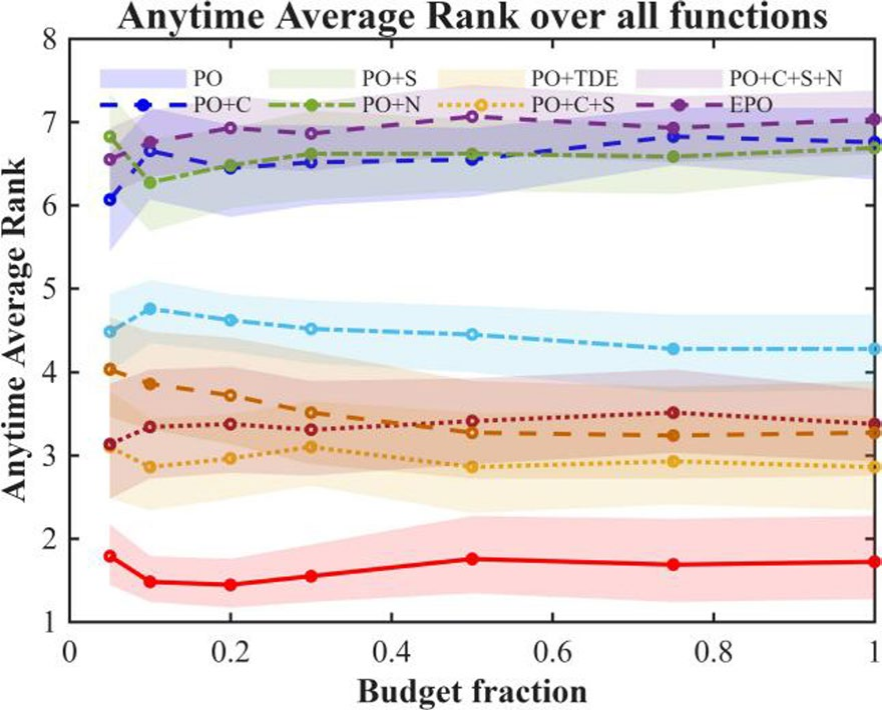
Anytime average rank.

**Fig. 4:**
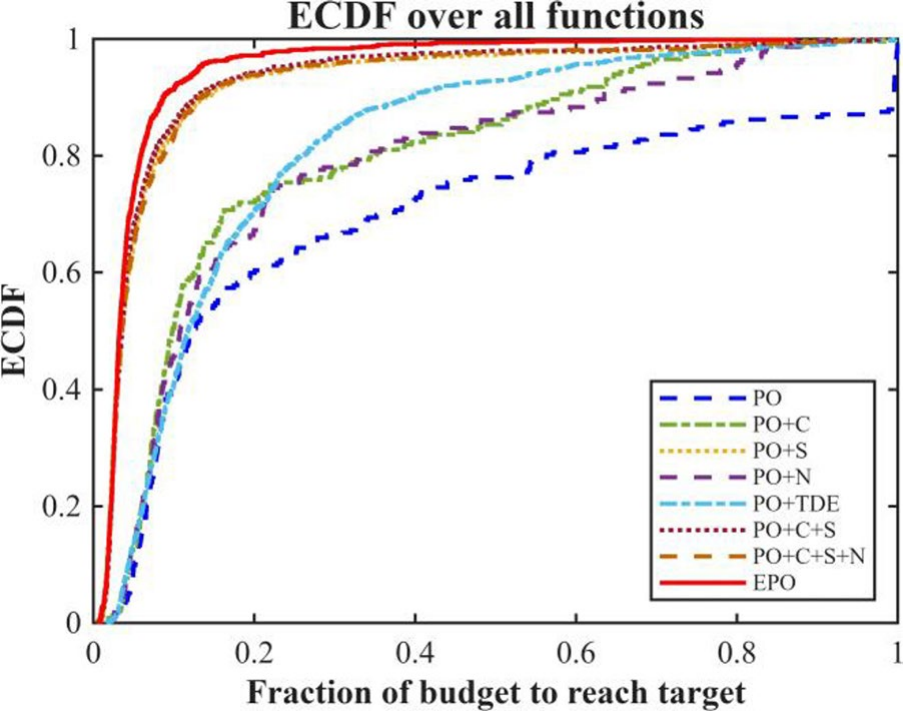
Time-to-target (ECDF).

We performed an ablation study employing a “path-additive” methodology to assess eight algorithmic variants: Base, +C, +S, +N, +TDE, + C + S, + C + S + N, and Full (+ C + S + N + TDE). The experiments were conducted under uniform settings: population = 30, dimensions D = 30, and a maximum budget of 20,00 × D function evaluations (MaxFEs), with 2 stages per generation. A selection of CEC 2017 benchmark functions was used for testing, with 30 independent runs per function under fixed random seeds. Performance was evaluated based on Average Rank, Number of Wins/Ties/Losses, Mean Success Rate (Ps), Mean Expected Running Time in iterations (ERTi_mean) and evaluations (ERTe_mean), Time-To-Target (ECDF), Anytime Average Rank (AAR), and Main Effects Analysis. The outcomes of this ablation experiment are illustrated in Figs. 2, 3 and 4; Table 2.

**Table 2:** Ablation study results.

Variant	AvgRank	Wins	Ties	Losses	Ps_mean	ERTe_mean
PO	6.7586	0	29	0	0.2667	48922.5172
PO + C	6.6897	15	0	14	0.2667	47237.7931
PO + S	2.8621	28	0	1	0.8828	11009.1724
PO + N	7.0345	13	0	16	0.2563	47926.8276
PO + TDE	4.2759	29	0	0	0.8609	17732.2414
PO + C + S	3.3793	27	0	2	0.8517	12537.5862
PO + C + S + N	3.2759	27	0	2	0.8563	12485.8621
EPO	1.7241	28	0	1	0.9276	7194.4138

The ablation study results in Figs. 2, 3 and 4; Table 2 show that the baseline PO has moderate performance. Ablation studies demonstrate that each proposed component exerts a significant and distinct influence on algorithmic performance. The Sparrow’s Vigilance Mechanism (S) serves as the primary driver of performance enhancement. The baseline algorithm PO exhibits poor performance (AvgRank = 6.76, Ps = 0.267, ERTe = 48,923), whereas incorporating S (PO + S) markedly reduces AvgRank to 2.86, increases the success probability to 0.883, and substantially decreases ERTe to 11,009. This improvement stems from S emulating the rapid evasion behavior of sparrows in response to perceived predation threats, thereby effectively enhancing the population’s local escape capability and mitigating premature convergence—consequently boosting both solution reliability and convergence efficiency.Chaotic Initialization (C), which leverages chaotic maps to enhance initial population diversity, yields limited standalone benefits: while PO + C increases the number of wins from 0 to 15, it fails to improve Ps (remaining at 0.267) and only marginally lowers AvgRank to 6.69. This suggests that merely improving initial solution quality cannot compensate for inherent deficiencies in the search mechanism. However, when C is integrated into the PO + S framework (PO + C + S), AvgRank further improves to 3.38 and ERTe decreases to 12,538, indicating that C provides valuable support in terms of high-quality starting points when embedded within an already efficient search architecture.The Non-linear Decreasing Factor (N), designed to dynamically adjust step sizes for balancing global exploration and local exploitation, exhibits adverse effects when applied alone: PO + N worsens AvgRank to 7.03, reflecting a tendency toward premature exploitation that traps the search in local optima. Nevertheless, when N is added to the PO + C + S configuration (PO + C + S + N), AvgRank slightly improves to 3.28, Ps rises to 0.856, and ERTe marginally drops to 12,486, confirming its role as an effective fine-tuning component in synergy with other mechanisms.The TDE strategy—applying t-distribution perturbations to inferior individuals to enhance exploration and employing differential evolution for refined exploitation of superior individuals—demonstrates exceptional robustness: PO + TDE achieves victory across all 29 benchmark problems (Wins = 29) with Ps = 0.861, albeit at a relatively high computational cost (ERTe = 17,732), highlighting its reliability at the expense of efficiency.Finally, the Enhanced PO (EPO), integrating all components, achieves the best overall performance (AvgRank = 1.72, Ps = 0.928, ERTe = 7,194), thereby validating the effectiveness of its synergistic multi-mechanism design in striking an optimal balance among solution success rate, stability, and computational efficiency.

### Population diversity and exploration–exploitation dynamics analysis (CEC2017, D = 30)

**Fig. 5:**
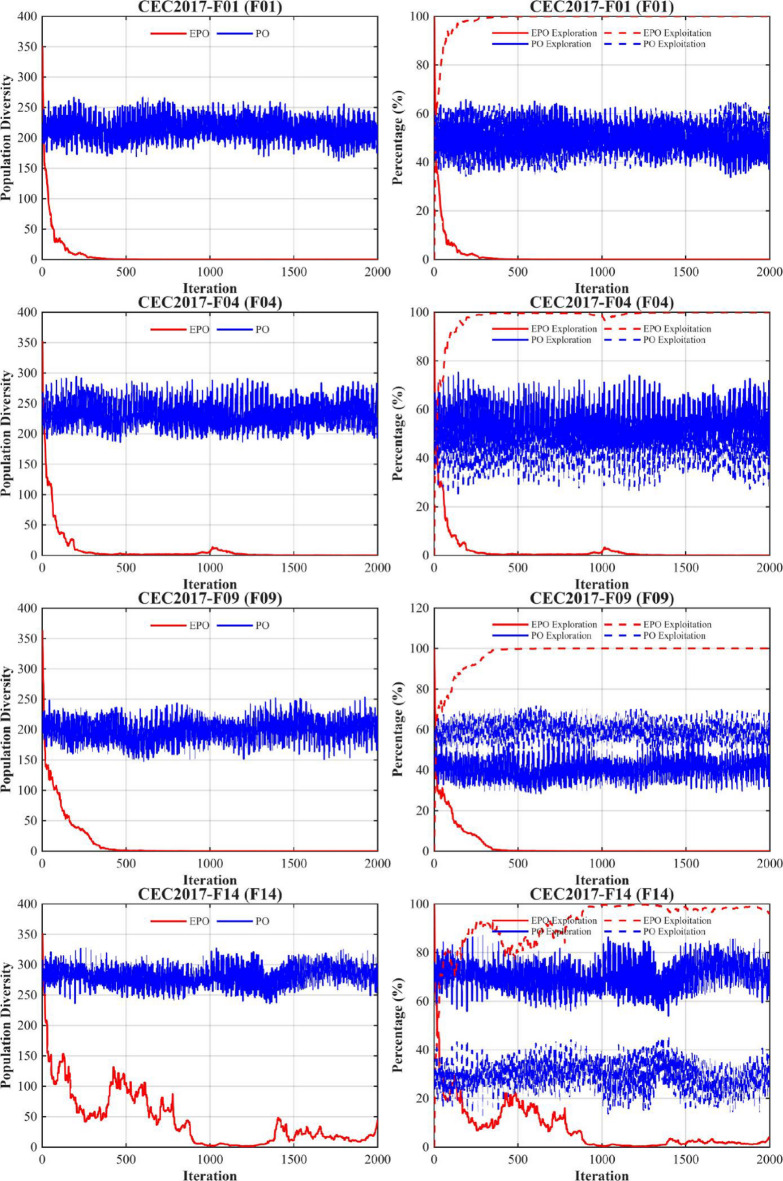

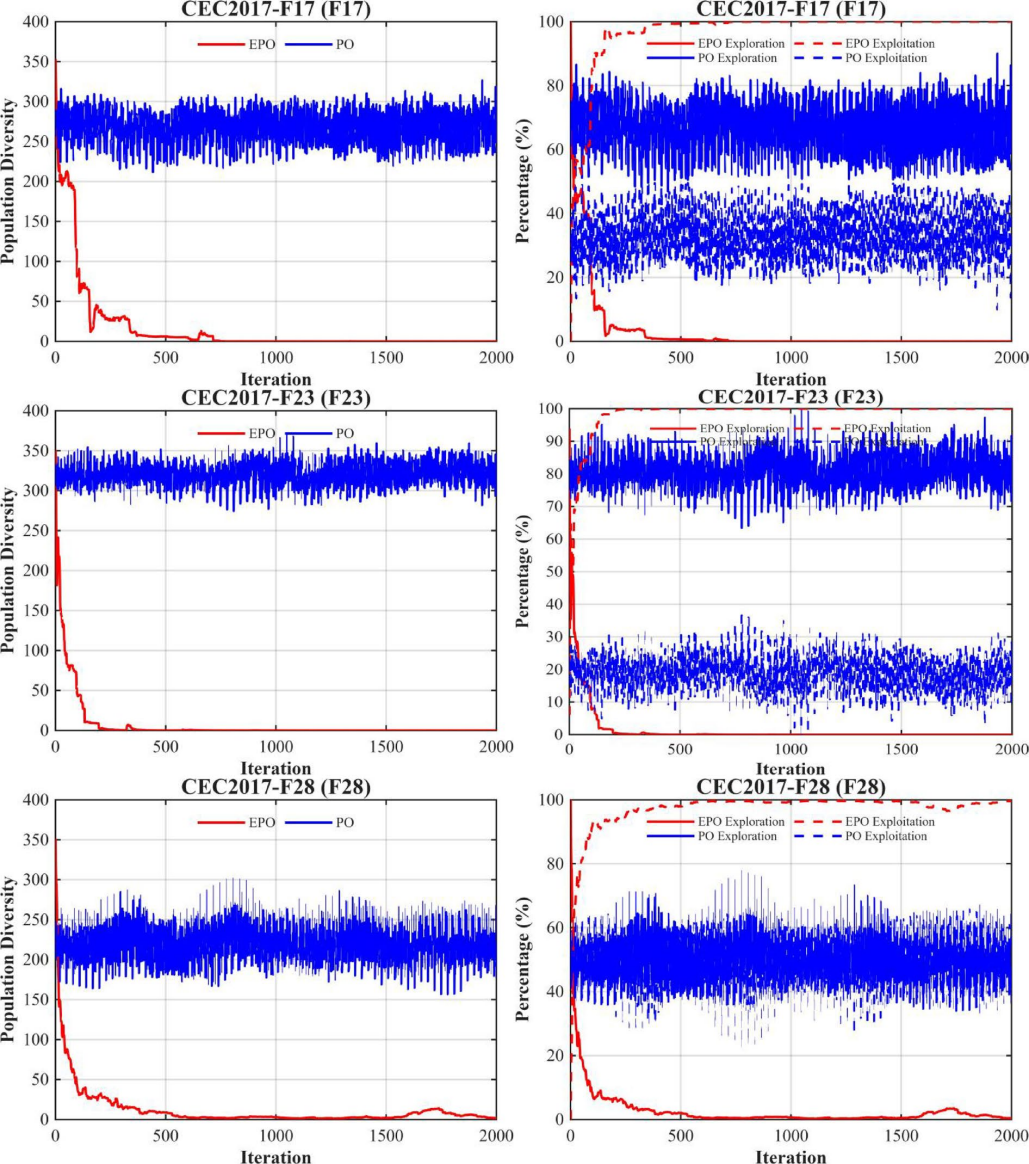
Population diversity and exploration–exploitation.

To gain deeper insight into the search mechanism advantages of the proposed EPO algorithm across optimization problems of varying complexity, this section presents a systematic comparative analysis of population diversity evolution and exploration–exploitation dynamics on representative functions from the CEC2017 benchmark suite. These include unimodal (F1), multimodal (F4, F9), hybrid (F14, F17), and highly complex composition functions (F23, F28). All experiments are conducted in 30 dimensions (D = 30), with a maximum of 2000 iterations and a population size of 30. The results of the population diversity and exploration–exploitation dynamics analysis are illustrated in Fig. 5.

As shown in Fig. 5, the EPO algorithm demonstrates significantly superior adaptive search behavior compared to the original PO across all benchmark functions, with particularly pronounced advantages on high-dimensional complex composition functions (e.g., F23 and F28). Its core strength lies in a well-structured three-phase search process: strong exploration in the early stage, smooth transition in the middle stage, and efficient convergence in the later stage. In contrast, PO consistently exhibits premature exploration decay, stagnant population diversity, and rigid search strategies.

(1) Population Diversity Evolution: EPO shows a rapidly decreasing diversity curve that converges toward zero across all test functions. Taking F28 as an example, EPO’s diversity drops below 10 within the first 500 iterations and approaches zero around iteration 600, indicating strong convergence guidance. Conversely, PO maintains a persistently high diversity level (150–300), reflecting widespread individual distribution but ineffective concentration toward the optimal region—manifesting a typical “ineffective diversity” phenomenon where the population remains dispersed yet fails to escape local optima. Notably, on F28, EPO exhibits a slight diversity rebound after iteration 1700, likely due to the TDE strategy applying t-distribution perturbations to inferior individuals, thereby enabling localized re-exploration and preventing complete entrapment in suboptimal regions.

(2) Exploration–Exploitation Dynamics: EPO consistently follows a clear “exploration-first, exploitation-later” pattern. Its exploration ratio declines sharply after approximately 300 iterations and stabilizes thereafter, facilitating precise exploitation and high-quality convergence. In contrast, PO’s exploration ratio remains fluctuating within 50%–80% throughout the run without a discernible downward trend, revealing a rigid search policy incapable of adapting to the current search state. This indicates that PO is trapped in a “blind search” mode—unable to effectively exploit promising regions or achieve stable convergence.

In summary, EPO achieves efficient convergence and robust performance across diverse optimization problems through rational diversity management and dynamic strategy adaptation. By contrast, the original PO algorithm, lacking an effective exploration–exploitation balancing mechanism, remains confined to local regions despite its relatively high initial diversity, ultimately failing to attain high-quality solutions. These results further validate the effectiveness of the proposed components—including the Sparrow’s Vigilance Mechanism, Chaotic Initialization, Non-linear Decreasing Factor, and TDE Strategy—in enhancing both global search capability and convergence efficiency.

## Experimental studies and results

According to the references^7,20,21,23,36^, the CEC2017 benchmark functions have been extensively employed to test the Optimizer. To prove the effectiveness of the Enhanced parrot Optimizer (EPO) put forward in this paper, we thoroughly tested it with the CEC2017 benchmark functions in 30, 50, and 100 dimensional spaces. The EPO is compared with the following optimization algorithms: Multistrategy Improved parrot Optimization (MPO)^49^, parrot Optimizer (PO)^46^, Starling Murmuration Optimizer (SMO)^7^, Dwarf Mongoose Optimization (DMOA)^18^, Dung Beetle Optimizer (DBO)^20^, Chernobyl Disaster Optimizer(CDO)^35^, Imperialist Competitive Algorithm (ICA)^25^, Gravitational Search Algorithm (GSA)^32^, and Hiking Optimization Algorithm (HOA)^29^.,Particle swarm optimization(PSO)^16^,Differential Evolution(DE)^12^.The hyperparameters of these algorithms are given in Table 3.

**Table 3:** Hyperparameters for correlation algorithms.

Name	Parameters
EPO	*β* = 1.5,α ∈ [0, 0.2], k = 5,δ ∈ [0, π], F0 = 0.25
MPO	*β* = 1.5,y = 0.2,z = 10, g = 0.5,δ ∈ [0, π]
PO	*β* = 1.5, α ∈ [0, 0.2],δ ∈ [0, π]
SMO	k = 5, λ = 20, µ = 0.5, θ, φ∈ (0, 1.8]
BTO	CUG = 6,67 × 10-11Nm2Kg-2,areamin = log(500000), areamax = log(1,510,000)
HOA	θ∈[0, 50◦], SF∈[1, 2]
HHO	β = 1.5,E_0_ ∈ [−1, 1]
DBO	k = 0.1,λ=0.1,b = 0.2,S = 0.5
BKA	*p* = 0.9, r ∈ [0, 1], c ∈ [0, 1]
IVY	beta1 = [1,1.5),GV = [0,1]

### Performance analysis on 30-dimensional functions

#### Statistics analysis

To assess performance, a comprehensive analysis was carried out by utilizing the 30-dimensional (D = 30) functions of the CEC2017 benchmark suite. The proposed algorithm was benchmarked against nine state-of-the-art metaheuristics, namely MPO, PO, SMO, BTO, HOA, HHO, DBO, BKA, and IVY. In all experiments, the population size (Np) was set at 30, the maximum number of iterations (Tmax) was 1,000^24^, and each algorithm was executed for 30 independent runs. Performance was quantified through five statistical indicators: minimum value ((Min), mean value ((Avg), median value (Median), worst value (Worst), and standard deviation (Std). These results are presented in Table 4.

**Table 4:** The statistical results of benchmark functions using the proposed technique and other algorithms (Dim = 30).

Function	Item	EPO	MPO	PO	SMO	BTO	HOA	HHO	DBO	BKA	IVY
F1	min	1.3941E + 02	6.0493E + 04	3.5957E + 07	2.2356E + 02	4.6328E + 10	2.7130E + 10	8.4159E + 06	1.8126E + 10	4.6640E + 06	**1.0422E + 02**
F1	std	**6.9156E + 03**	1.9694E + 08	3.1130E + 08	2.0644E + 05	2.7055E + 10	5.5349E + 09	3.6143E + 06	3.3067E + 09	1.5587E + 10	5.3927E + 08
F1	avg	**7.2400E + 03**	4.0054E + 07	3.1611E + 08	4.4981E + 04	7.3217E + 10	3.6036E + 10	1.5111E + 07	2.6548E + 10	7.1156E + 09	1.5453E + 08
F1	avg_time	3.69E-01	4.37E-01	4.85E-01	4.8603E + 00	7.57E-01	**1.65E-01**	3.95E-01	2.64E-01	2.90E-01	7.11E-01
F3	min	**3.0123E + 02**	1.2430E + 04	2.4894E + 04	3.3240E + 02	7.7994E + 04	4.6896E + 04	1.3151E + 04	3.5809E + 04	3.1473E + 03	2.6894E + 04
F3	std	**2.9472E + 02**	5.9744E + 03	6.7200E + 03	2.1956E + 03	4.4866E + 03	1.0133E + 04	5.4068E + 03	1.0248E + 04	1.6669E + 04	9.5291E + 03
F3	avg	**4.4052E + 02**	2.6558E + 04	3.9966E + 04	1.7751E + 03	8.8464E + 04	6.9781E + 04	2.2678E + 04	6.4216E + 04	1.4530E + 04	4.4056E + 04
F3	avg_time	3.65E-01	4.38E-01	4.74E-01	4.7924E + 00	7.42E-01	**1.66E-01**	3.99E-01	2.82E-01	2.88E-01	7.14E-01
F4	min	**4.0400E + 02**	4.7739E + 02	4.8626E + 02	4.1703E + 02	8.6752E + 03	4.2166E + 03	4.7963E + 02	2.0766E + 03	4.8980E + 02	4.6930E + 02
F4	std	2.3977E + 01	2.9464E + 01	5.5612E + 01	2.8711E + 01	7.7725E + 03	1.6228E + 03	2.3227E + 01	1.8899E + 03	3.8470E + 03	**2.0178E + 01**
F4	avg	**4.8514E + 02**	5.2206E + 02	5.8673E + 02	4.9426E + 02	1.5005E + 04	6.9432E + 03	5.3299E + 02	4.3934E + 03	1.9026E + 03	4.9635E + 02
F4	avg_time	3.56E-01	4.32E-01	4.81E-01	4.8554E + 00	7.50E-01	**1.66E-01**	3.95E-01	2.65E-01	2.92E-01	7.05E-01
F5	min	**5.8159E + 02**	5.8909E + 02	6.5386E + 02	6.0845E + 02	9.0730E + 02	7.3856E + 02	6.6795E + 02	7.4127E + 02	6.7123E + 02	6.2238E + 02
F5	std	4.0658E + 01	4.4050E + 01	3.7086E + 01	4.4632E + 01	5.3029E + 01	3.2124E + 01	**2.6979E + 01**	2.8688E + 01	2.7168E + 01	4.8475E + 01
F5	avg	**6.6555E + 02**	6.9574E + 02	7.3634E + 02	6.6719E + 02	9.5582E + 02	8.1887E + 02	7.3548E + 02	8.0856E + 02	7.2766E + 02	7.1113E + 02
F5	avg_time	6.09E-01	5.91E-01	6.28E-01	4.9069E + 00	8.16E-01	**2.44E-01**	5.92E-01	3.42E-01	4.54E-01	7.89E-01
F6	min	6.1540E + 02	6.3081E + 02	6.3713E + 02	6.1955E + 02	6.8544E + 02	6.4335E + 02	6.5011E + 02	6.5097E + 02	6.4630E + 02	**6.0243E + 02**
F6	std	1.1715E + 01	1.1849E + 01	9.3063E + 00	7.5716E + 00	1.4597E + 01	7.6891E + 00	**4.9026E + 00**	6.6512E + 00	8.0205E + 00	1.4432E + 01
F6	avg	6.3766E + 02	6.5014E + 02	6.6268E + 02	6.3413E + 02	6.9903E + 02	6.6558E + 02	6.6317E + 02	6.6350E + 02	6.6240E + 02	**6.2162E + 02**
F6	avg_time	7.00E-01	9.74E-01	1.0134E + 00	5.1582E + 00	1.0158E + 00	**4.38E-01**	1.0654E + 00	5.45E-01	8.28E-01	1.0139E + 00
F7	min	**8.4180E + 02**	9.3077E + 02	1.0196E + 03	9.0657E + 02	1.3675E + 03	1.0853E + 03	1.1116E + 03	1.1216E + 03	1.0027E + 03	1.0076E + 03
F7	std	8.0379E + 01	7.6658E + 01	7.7113E + 01	9.8062E + 01	4.6308E + 02	6.4743E + 01	6.6992E + 01	**3.6811E + 01**	6.7902E + 01	8.8413E + 01
F7	avg	**9.9487E + 02**	1.0522E + 03	1.1418E + 03	1.0723E + 03	1.5769E + 03	1.2532E + 03	1.2534E + 03	1.1809E + 03	1.2188E + 03	1.2244E + 03
F7	avg_time	5.92E-01	6.04E-01	6.46E-01	4.9895E + 00	8.40E-01	**2.50E-01**	6.08E-01	3.43E-01	4.62E-01	8.06E-01
F8	min	**8.5174E + 02**	9.1434E + 02	9.2724E + 02	8.5970E + 02	1.1286E + 03	1.0270E + 03	9.1371E + 02	9.8871E + 02	9.0721E + 02	8.7761E + 02
F8	std	3.0730E + 01	3.2024E + 01	3.0336E + 01	3.1922E + 01	3.3077E + 01	**2.3516E + 01**	2.7549E + 01	2.5973E + 01	2.9851E + 01	3.5161E + 01
F8	avg	9.3401E + 02	9.6831E + 02	9.8771E + 02	**9.2785E + 02**	1.1671E + 03	1.0689E + 03	9.6731E + 02	1.0420E + 03	9.6954E + 02	9.5363E + 02
F8	avg_time	5.95E-01	5.96E-01	6.39E-01	4.9780E + 00	8.30E-01	**2.53E-01**	6.03E-01	3.47E-01	4.60E-01	8.16E-01
F9	min	2.1269E + 03	3.4965E + 03	3.3903E + 03	**1.9525E + 03**	7.8326E + 03	4.7640E + 03	5.7313E + 03	5.2987E + 03	3.4284E + 03	3.3818E + 03
F9	std	1.0029E + 03	9.2795E + 02	1.0313E + 03	1.0586E + 03	6.8468E + 03	1.0464E + 03	8.0637E + 02	1.1283E + 03	6.7818E + 02	**4.3097E + 02**
F9	avg	**3.5851E + 03**	5.0957E + 03	6.3595E + 03	3.9474E + 03	1.3823E + 04	6.9455E + 03	7.7034E + 03	7.1038E + 03	4.8668E + 03	5.2821E + 03
F9	avg_time	4.62E-01	5.95E-01	6.47E-01	4.9736E + 00	8.33E-01	**2.50E-01**	6.11E-01	3.51E-01	4.58E-01	7.90E-01
F10	min	3.8625E + 03	3.7380E + 03	4.3944E + 03	**3.6092E + 03**	7.6026E + 03	5.3157E + 03	4.4021E + 03	6.5325E + 03	3.9932E + 03	3.8912E + 03
F10	std	6.5343E + 02	7.1732E + 02	8.0727E + 02	6.0803E + 02	**4.2820E + 02**	6.0237E + 02	5.9211E + 02	5.9597E + 02	9.6186E + 02	6.9315E + 02
F10	avg	5.0691E + 03	5.3374E + 03	5.9772E + 03	**4.8607E + 03**	8.7624E + 03	7.2883E + 03	5.4462E + 03	7.8702E + 03	5.2914E + 03	5.2874E + 03
F10	avg_time	5.90E-01	7.04E-01	7.45E-01	5.0058E + 00	8.74E-01	**2.93E-01**	7.27E-01	4.22E-01	5.50E-01	8.53E-01
F11	min	1.1582E + 03	1.2606E + 03	1.2747E + 03	1.1768E + 03	6.9354E + 03	2.3068E + 03	1.1736E + 03	2.2031E + 03	1.1993E + 03	**1.1375E + 03**
F11	std	7.3988E + 01	9.6925E + 01	1.4692E + 02	5.6806E + 01	1.7239E + 05	2.0855E + 03	**3.9920E + 01**	1.0444E + 03	2.3263E + 03	5.5566E + 01
F11	avg	1.2475E + 03	1.3690E + 03	1.5451E + 03	1.2979E + 03	7.0198E + 04	6.1425E + 03	1.2584E + 03	4.1925E + 03	2.2195E + 03	**1.2452E + 03**
F11	avg_time	4.08E-01	4.98E-01	5.48E-01	4.9207E + 00	7.81E-01	**2.02E-01**	4.90E-01	3.09E-01	3.65E-01	7.48E-01
F12	min	**8.0491E + 03**	1.8526E + 06	7.6601E + 06	1.4049E + 04	6.0115E + 09	1.2102E + 09	3.2171E + 06	5.9124E + 08	2.3042E + 06	2.3382E + 05
F12	std	8.4759E + 06	3.0449E + 07	8.3466E + 07	**2.3781E + 05**	5.6431E + 09	1.9240E + 09	1.1015E + 07	1.6502E + 09	8.2899E + 08	1.1812E + 07
F12	avg	4.0034E + 06	2.8485E + 07	1.0067E + 08	**2.0697E + 05**	1.6357E + 10	6.3836E + 09	1.7535E + 07	5.2094E + 09	1.6585E + 08	3.1765E + 06
F12	avg_time	4.52E-01	5.86E-01	6.32E-01	4.9471E + 00	8.10E-01	**2.42E-01**	5.77E-01	3.44E-01	4.41E-01	8.12E-01
F13	min	6.7135E + 03	1.4451E + 04	5.1673E + 04	**3.5244E + 03**	1.7645E + 09	5.1891E + 08	1.8562E + 05	2.5570E + 07	4.7958E + 04	6.6785E + 03
F13	std	3.1720E + 07	2.9437E + 06	2.8245E + 06	**1.4303E + 04**	8.4345E + 09	2.1419E + 09	2.0645E + 05	1.9093E + 09	1.8625E + 08	2.0547E + 04
F13	avg	2.0058E + 07	6.5347E + 05	1.1803E + 06	**1.7050E + 04**	1.3198E + 10	3.0284E + 09	4.6603E + 05	1.8775E + 09	3.9571E + 07	2.7714E + 04
F13	avg_time	4.90E-01	5.14E-01	5.59E-01	4.9871E + 00	7.92E-01	**2.06E-01**	4.93E-01	3.16E-01	3.69E-01	7.56E-01
F14	min	2.7626E + 03	1.2784E + 04	2.2707E + 03	**1.5718E + 03**	7.1653E + 05	2.5668E + 04	2.6895E + 04	4.3259E + 04	1.6484E + 03	1.0861E + 04
F14	std	3.6912E + 04	1.6202E + 05	4.5293E + 05	**1.9807E + 03**	4.6472E + 07	8.8520E + 05	2.4328E + 05	4.6517E + 05	2.5012E + 04	1.2471E + 06
F14	avg	3.2948E + 04	1.4983E + 05	4.0116E + 05	**3.0177E + 03**	1.7558E + 07	1.4807E + 06	2.1226E + 05	3.9062E + 05	1.2071E + 04	7.6240E + 05
F14	avg_time	5.27E-01	6.94E-01	7.46E-01	4.9976E + 00	8.68E-01	**2.95E-01**	7.15E-01	4.11E-01	5.54E-01	8.45E-01
F15	min	2.5304E + 03	3.2468E + 03	1.5630E + 04	**1.6721E + 03**	7.0561E + 07	1.2111E + 06	2.0968E + 04	2.5982E + 04	4.0790E + 03	2.0239E + 03
F15	std	1.6910E + 04	7.7709E + 04	2.7610E + 05	**1.1862E + 04**	1.8403E + 09	1.4384E + 08	2.9063E + 04	3.8920E + 06	1.4130E + 04	6.9917E + 05
F15	avg	2.3201E + 04	3.9024E + 04	1.4902E + 05	**1.1201E + 04**	1.4655E + 09	1.4359E + 08	6.4531E + 04	3.1055E + 06	2.6532E + 04	1.3286E + 05
F15	avg_time	4.07E-01	4.95E-01	5.34E-01	4.9190E + 00	7.71E-01	**1.99E-01**	4.70E-01	2.97E-01	3.51E-01	7.46E-01
F16	min	2.0999E + 03	2.1858E + 03	2.5327E + 03	2.2291E + 03	5.0284E + 03	3.2283E + 03	2.6810E + 03	2.9899E + 03	2.3043E + 03	**2.0972E + 03**
F16	std	3.8046E + 02	3.9666E + 02	4.5154E + 02	**2.9789E + 02**	2.4424E + 03	8.1613E + 02	4.1073E + 02	3.6137E + 02	3.0607E + 02	3.7817E + 02
F16	avg	2.8757E + 03	2.9320E + 03	3.3530E + 03	**2.7285E + 03**	7.3842E + 03	4.7962E + 03	3.4494E + 03	3.6916E + 03	2.9586E + 03	2.9055E + 03
F16	avg_time	5.24E-01	5.65E-01	6.06E-01	4.9205E + 00	8.04E-01	**2.27E-01**	5.52E-01	3.38E-01	4.20E-01	7.78E-01
F17	min	1.8895E + 03	1.8990E + 03	2.0714E + 03	1.9996E + 03	3.6864E + 03	2.2702E + 03	2.0426E + 03	2.2774E + 03	1.9450E + 03	**1.8868E + 03**
F17	std	3.0133E + 02	2.8849E + 02	2.1012E + 02	1.7091E + 02	1.2965E + 04	4.4701E + 02	2.6831E + 02	2.8075E + 02	**1.6832E + 02**	3.1361E + 02
F17	avg	2.5734E + 03	2.4134E + 03	2.4389E + 03	2.3293E + 03	1.1860E + 04	2.8998E + 03	2.5748E + 03	2.6791E + 03	**2.3287E + 03**	2.6225E + 03
F17	avg_time	6.64E-01	8.59E-01	8.99E-01	5.1376E + 00	9.45E-01	**3.73E-01**	9.13E-01	4.91E-01	7.14E-01	9.40E-01
F18	min	1.1263E + 04	1.8114E + 05	1.3855E + 05	**4.9709E + 03**	1.0408E + 07	1.1214E + 06	8.9172E + 04	1.3990E + 05	2.4285E + 04	5.2174E + 04
F18	std	3.0517E + 05	1.5306E + 06	2.5375E + 06	**2.9001E + 04**	1.4167E + 08	1.0669E + 07	1.4793E + 06	1.9459E + 06	9.5497E + 05	2.2858E + 06
F18	avg	2.1546E + 05	1.5605E + 06	2.6544E + 06	**4.0289E + 04**	8.9614E + 07	1.2731E + 07	1.3094E + 06	2.3791E + 06	3.1355E + 05	1.4708E + 06
F18	avg_time	4.46E-01	5.63E-01	6.18E-01	4.8914E + 00	8.16E-01	**2.33E-01**	5.61E-01	3.49E-01	4.22E-01	7.85E-01
F19	min	2.0528E + 03	2.2213E + 03	5.5652E + 03	**2.0436E + 03**	1.6735E + 08	2.2426E + 06	6.9487E + 04	1.9333E + 06	1.1328E + 04	2.2855E + 03
F19	std	1.0064E + 05	5.8225E + 05	2.6899E + 06	1.0761E + 04	2.6513E + 09	4.2686E + 07	3.1207E + 05	1.5288E + 08	7.3318E + 06	**4.6129E + 03**
F19	avg	4.3151E + 04	2.6818E + 05	3.9807E + 06	1.0972E + 04	1.9257E + 09	3.7790E + 07	5.2515E + 05	1.5520E + 08	2.3847E + 06	**6.5807E + 03**
F19	avg_time	1.7821E + 00	2.8164E + 00	2.7935E + 00	6.1631E + 00	1.9011E + 00	**1.3255E + 00**	3.1979E + 00	1.4291E + 00	2.5955E + 00	1.9178E + 00
F20	min	2.4032E + 03	**2.1454E + 03**	2.2961E + 03	2.1740E + 03	2.7150E + 03	2.2951E + 03	2.3771E + 03	2.3497E + 03	2.3142E + 03	2.3056E + 03
F20	std	1.5345E + 02	1.4752E + 02	1.8422E + 02	2.3834E + 02	2.7643E + 02	1.9978E + 02	2.2126E + 02	**1.4416E + 02**	1.6445E + 02	2.2119E + 02
F20	avg	2.7989E + 03	**2.5420E + 03**	2.6919E + 03	2.5972E + 03	3.1203E + 03	2.7269E + 03	2.8201E + 03	2.5646E + 03	2.5937E + 03	2.8150E + 03
F20	avg_time	7.92E-01	8.93E-01	9.30E-01	5.2807E + 00	9.74E-01	**3.96E-01**	9.74E-01	5.19E-01	7.49E-01	9.62E-01
F21	min	2.3852E + 03	2.3939E + 03	2.4299E + 03	2.3773E + 03	2.7040E + 03	2.5151E + 03	2.4886E + 03	**2.2579E + 03**	2.4160E + 03	2.3430E + 03
F21	std	5.5610E + 01	4.3334E + 01	4.3305E + 01	**3.3004E + 01**	7.1181E + 01	4.2220E + 01	4.0895E + 01	1.0957E + 02	6.1203E + 01	4.1196E + 01
F21	avg	2.4565E + 03	2.4626E + 03	2.5235E + 03	2.4443E + 03	2.7806E + 03	2.6082E + 03	2.5742E + 03	2.4993E + 03	2.5223E + 03	**2.4009E + 03**
F21	avg_time	9.56E-01	1.0641E + 00	1.1021E + 00	5.2415E + 00	1.0657E + 00	**4.67E-01**	1.1337E + 00	5.84E-01	8.95E-01	1.0297E + 00
F22	min	**2.3000E + 03**	2.3032E + 03	2.3614E + 03	2.3001E + 03	8.7755E + 03	5.7081E + 03	2.3239E + 03	4.1284E + 03	2.3722E + 03	2.3000E + 03
F22	std	2.2162E + 03	1.7056E + 03	2.0231E + 03	2.1373E + 03	7.4354E + 02	9.8351E + 02	1.2668E + 03	**5.0495E + 02**	2.0826E + 03	2.4915E + 03
F22	avg	3.9448E + 03	**3.1061E + 03**	3.4867E + 03	4.4891E + 03	9.6855E + 03	8.1579E + 03	7.1079E + 03	5.3154E + 03	5.9533E + 03	4.9828E + 03
F22	avg_time	9.27E-01	1.2357E + 00	1.2744E + 00	5.3537E + 00	1.1116E + 00	**5.48E-01**	1.3661E + 00	6.70E-01	1.0707E + 00	1.1353E + 00
F23	min	2.7646E + 03	2.7841E + 03	2.8517E + 03	2.7958E + 03	3.3183E + 03	3.3075E + 03	2.8602E + 03	2.9495E + 03	2.8856E + 03	**2.7101E + 03**
F23	std	9.7973E + 01	7.2669E + 01	7.0915E + 01	**5.3710E + 01**	2.2374E + 02	1.5386E + 02	1.3039E + 02	8.1167E + 01	1.0632E + 02	8.6694E + 01
F23	avg	2.9025E + 03	2.9043E + 03	2.9907E + 03	2.8793E + 03	3.6523E + 03	3.5194E + 03	3.1587E + 03	3.0911E + 03	3.1109E + 03	**2.8078E + 03**
F23	avg_time	1.1167E + 00	1.3095E + 00	1.3312E + 00	5.3736E + 00	1.1473E + 00	**5.85E-01**	1.4230E + 00	7.04E-01	1.1476E + 00	1.2000E + 00
F24	min	2.9591E + 03	2.9540E + 03	2.9642E + 03	2.9337E + 03	3.5295E + 03	3.5825E + 03	3.1341E + 03	3.1381E + 03	3.0225E + 03	**2.9101E + 03**
F24	std	**6.4102E + 01**	8.3318E + 01	7.6617E + 01	7.3710E + 01	3.0070E + 02	1.3998E + 02	1.5512E + 02	1.0650E + 02	1.1682E + 02	1.1517E + 02
F24	avg	3.0569E + 03	3.0733E + 03	3.1248E + 03	3.0463E + 03	3.9803E + 03	3.8597E + 03	3.4187E + 03	3.3139E + 03	3.2970E + 03	**2.9976E + 03**
F24	avg_time	1.3783E + 00	1.4698E + 00	1.5067E + 00	5.5539E + 00	1.2725E + 00	**6.80E-01**	1.6612E + 00	7.91E-01	1.3157E + 00	1.2541E + 00
F25	min	**2.8835E + 03**	2.8976E + 03	2.9101E + 03	2.8842E + 03	4.2996E + 03	3.3882E + 03	2.8904E + 03	3.3387E + 03	2.8868E + 03	2.8836E + 03
F25	std	**1.2733E + 01**	2.1165E + 01	3.4562E + 01	2.0488E + 01	4.2230E + 03	2.8547E + 02	2.4860E + 01	2.0700E + 02	4.1258E + 02	2.1555E + 01
F25	avg	**2.8921E + 03**	2.9368E + 03	2.9826E + 03	2.9067E + 03	6.4201E + 03	3.8394E + 03	2.9166E + 03	3.7954E + 03	3.1042E + 03	2.9035E + 03
F25	avg_time	9.37E-01	1.1139E + 00	1.1441E + 00	5.3085E + 00	1.0815E + 00	**4.95E-01**	1.1873E + 00	5.96E-01	9.57E-01	1.0835E + 00
F26	min	2.9000E + 03	2.8096E + 03	3.2784E + 03	2.8001E + 03	1.0562E + 04	7.4048E + 03	2.8895E + 03	6.1858E + 03	3.7573E + 03	**2.8000E + 03**
F26	std	7.8406E + 02	1.5886E + 03	1.8302E + 03	1.3350E + 03	1.3266E + 03	9.7040E + 02	1.8127E + 03	**5.7442E + 02**	1.5890E + 03	1.8858E + 03
F26	avg	**5.7486E + 03**	5.7686E + 03	6.2981E + 03	6.0017E + 03	1.2194E + 04	9.4172E + 03	6.9032E + 03	7.2640E + 03	7.8024E + 03	6.3793E + 03
F26	avg_time	1.3899E + 00	1.6030E + 00	1.6183E + 00	5.5468E + 00	1.2714E + 00	**7.22E-01**	1.7481E + 00	8.35E-01	1.4138E + 00	1.3025E + 00
F27	min	**3.2151E + 03**	3.2442E + 03	3.2325E + 03	3.2249E + 03	3.6495E + 03	3.9453E + 03	3.2423E + 03	3.3697E + 03	3.2677E + 03	3.2457E + 03
F27	std	**3.1508E + 01**	4.4041E + 01	8.6340E + 01	5.6397E + 01	5.6135E + 02	2.4169E + 02	1.0270E + 02	1.4615E + 02	1.2284E + 02	6.4537E + 01
F27	avg	**3.2691E + 03**	3.3009E + 03	3.3626E + 03	3.2853E + 03	4.4279E + 03	4.3768E + 03	3.3681E + 03	3.6343E + 03	3.4446E + 03	3.3275E + 03
F27	avg_time	1.4340E + 00	1.7241E + 00	1.7592E + 00	5.5808E + 00	1.3679E + 00	**7.92E-01**	1.9167E + 00	9.22E-01	1.5541E + 00	1.3802E + 00
F28	min	**3.1000E + 03**	3.2190E + 03	3.3034E + 03	3.1981E + 03	6.1637E + 03	4.8042E + 03	3.2275E + 03	4.0508E + 03	3.1988E + 03	3.1998E + 03
F28	std	4.5214E + 01	4.1028E + 01	4.6519E + 01	2.9629E + 01	2.4598E + 03	4.6833E + 02	**2.2010E + 01**	4.5809E + 02	9.3821E + 02	2.2649E + 01
F28	avg	**3.2181E + 03**	3.3064E + 03	3.3760E + 03	3.2470E + 03	7.9589E + 03	5.6977E + 03	3.2696E + 03	4.9816E + 03	3.5654E + 03	3.2364E + 03
F28	avg_time	9.38E-01	1.3869E + 00	1.4249E + 00	5.4590E + 00	1.1969E + 00	**6.37E-01**	1.5279E + 00	7.38E-01	1.2327E + 00	1.2074E + 00
F29	min	3.7700E + 03	3.7654E + 03	4.0083E + 03	3.8202E + 03	6.4633E + 03	5.1046E + 03	3.7805E + 03	3.7845E + 03	3.8095E + 03	**3.6381E + 03**
F29	std	**2.6769E + 02**	3.3518E + 02	4.3649E + 02	2.8912E + 02	4.3831E + 03	9.1806E + 02	3.5652E + 02	2.9775E + 02	6.6015E + 02	3.3699E + 02
F29	avg	**4.1282E + 03**	4.4266E + 03	4.8009E + 03	4.2678E + 03	8.7173E + 03	6.3910E + 03	4.5210E + 03	4.6128E + 03	4.6145E + 03	4.4880E + 03
F29	avg_time	8.87E-01	1.1984E + 00	1.2341E + 00	5.3501E + 00	1.1341E + 00	**5.48E-01**	1.3212E + 00	6.57E-01	1.0511E + 00	1.1236E + 00
F30	min	**6.0243E + 03**	8.4174E + 04	3.7725E + 06	6.5271E + 03	4.0052E + 08	4.6662E + 07	4.6028E + 05	1.3389E + 07	1.0662E + 05	9.1192E + 03
F30	std	3.1715E + 05	5.3253E + 06	9.1424E + 06	**3.0398E + 03**	1.9216E + 09	3.9988E + 08	1.5747E + 06	4.6308E + 07	4.6359E + 07	1.0238E + 04
F30	avg	1.4243E + 05	4.2785E + 06	1.5936E + 07	**1.0793E + 04**	3.3051E + 09	5.4873E + 08	2.9180E + 06	6.4074E + 07	1.8188E + 07	2.4222E + 04
F30	avg_time	1.9529E + 00	3.1057E + 00	3.1056E + 00	6.3497E + 00	1.9987E + 00	**1.4757E + 00**	3.5662E + 00	1.5914E + 00	2.9038E + 00	2.0694E + 00

According to Table 4^4^ the following conclusions can be drawn:

1. **Unimodal Functions (F1**,** F3).**EPO achieves the best results among all compared algorithms in terms of both average (“avg”) and standard deviation (“std”) metrics on F1 and F3, with a win rate of 33% for each metric. Regarding the best-found (“min”) value, EPO obtains the optimal result on F3, while ranking second on F1—its performance is only 33.8% worse than that of IVY, the top performer. These results indicate that EPO exhibits high convergence accuracy and good stability when solving simple convex optimization problems. However, the average runtime of EPO is approximately 2.2 times longer than that of the fastest algorithm, HOA, suggesting that the performance gains come at the cost of computational efficiency.
2. **Simple Multimodal Functions (F4–F10).**EPO secures the best “avg” and “min” results on four functions each (win rate: 19% for both metrics). Specifically, it achieves the lowest average values on F4, F5, F7, and F9, and the best minimum values on F4, F5, F7, and F8. On the remaining functions, EPO remains competitive: its “avg” results on F6, F8, and F10 rank within the top three, with deviations from the best values not exceeding 4.3%; similarly, its “min” results on F6, F9, and F10 are among the best, with gaps ≤ 8.9%. Nevertheless, EPO does not achieve any best “std” result across this category, indicating relatively large performance fluctuations across independent runs and highlighting room for improvement in robustness. Additionally, EPO’s runtime is consistently 1.8 to 2.5 times that of HOA, further illustrating the trade-off between solution quality and computational efficiency.
3. **Hybrid Functions (F11–F20).**EPO wins only once in the “min” metric—on F12 (win rate: 3%). On functions such as F11 and F15–F19, EPO typically ranks second or third in both “avg” and “min” metrics. For instance, on F11, its average result deviates by merely 0.2% from the best; on F16–F17, the gap in the best-found value is ≤ 0.1%. However, on F12–F14 and F18–F19, EPO lags significantly behind the top-performing algorithms—particularly SMO—with differences reaching orders of magnitude (e.g., a staggering 117,545.0% gap in the average value on F13). Moreover, EPO exhibits substantially larger standard deviations across most hybrid functions; on F13, F14, and F18–F19, its “std” values exceed those of the best algorithm by hundreds to thousands of times, revealing insufficient stability when tackling high-dimensional, nonlinear hybrid problems.
4. **Composition Functions (F21–F30).**EPO demonstrates strong performance in this most challenging category, achieving the best results 5 times in “avg” (17%), 5 times in “min” (17%), and 4 times in “std” (13%). The optimal “avg” and “min” performances are concentrated on F25–F29, while the best “std” values occur on F24, F25, F27, and F29. On the remaining composition functions, EPO remains robust: for F21–F24, its “avg” and “min” rankings are mostly second to fourth, with performance gaps generally below 5%; on F26, F28, and F29, its “min” and “std” results are also close to optimal. Although EPO’s runtime remains higher than HOA’s (by 32% to 105%), its superior solution quality and stability confirm its effectiveness in handling highly complex, non-separable, rotated, and shifted optimization landscapes.
5. Overall Comparison with PO.Across the 116 valid test scenarios (29 functions × 4 metrics: avg, min, std, time), EPO outperforms the original PO algorithm with an overall win rate of 0.87. Notably, EPO shows clear advantages over PO in the following metrics: win rate of avg is 0.86,win rate of avg_time is 1.00 (i.e., EPO is always faster than PO), win rate of min is0.93.In summary, EPO consistently surpasses PO across most evaluation criteria, demonstrating significantly enhanced competitiveness and validating the efficacy of the proposed improvements.

**Fig. 6:**
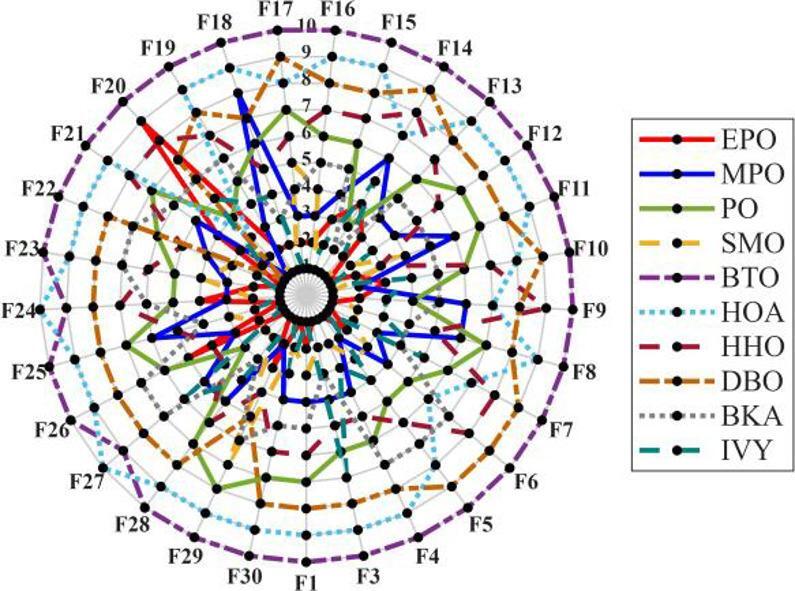
Radar chart.

**Fig. 7:**
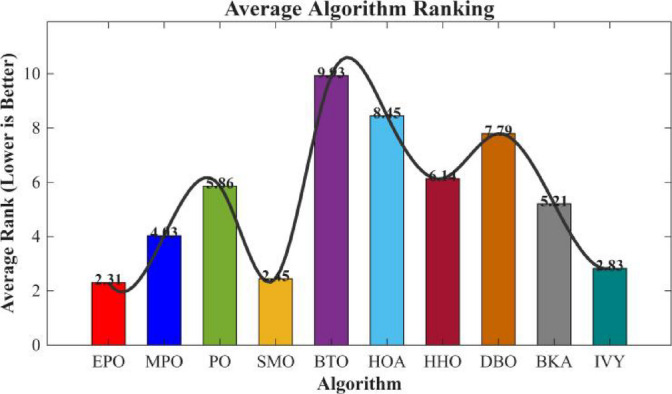
Average algorithmic rankings.

**Fig. 8:**
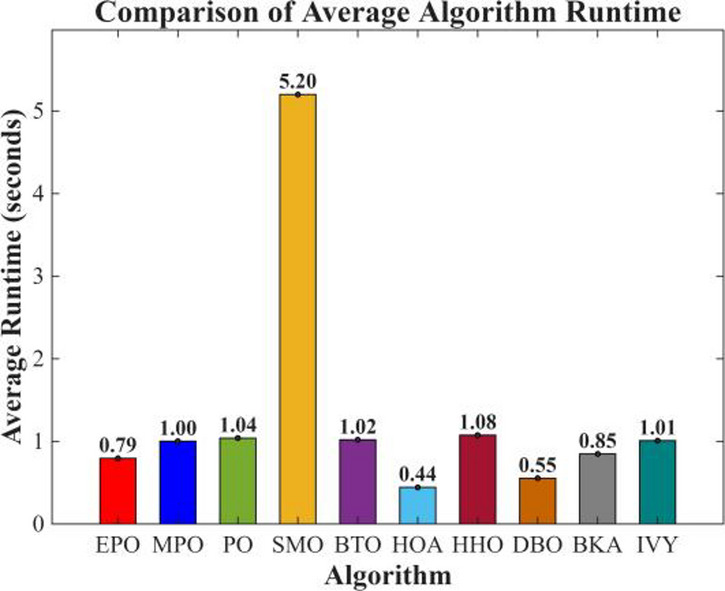
Comparison of average runtime.

**Fig. 9:**
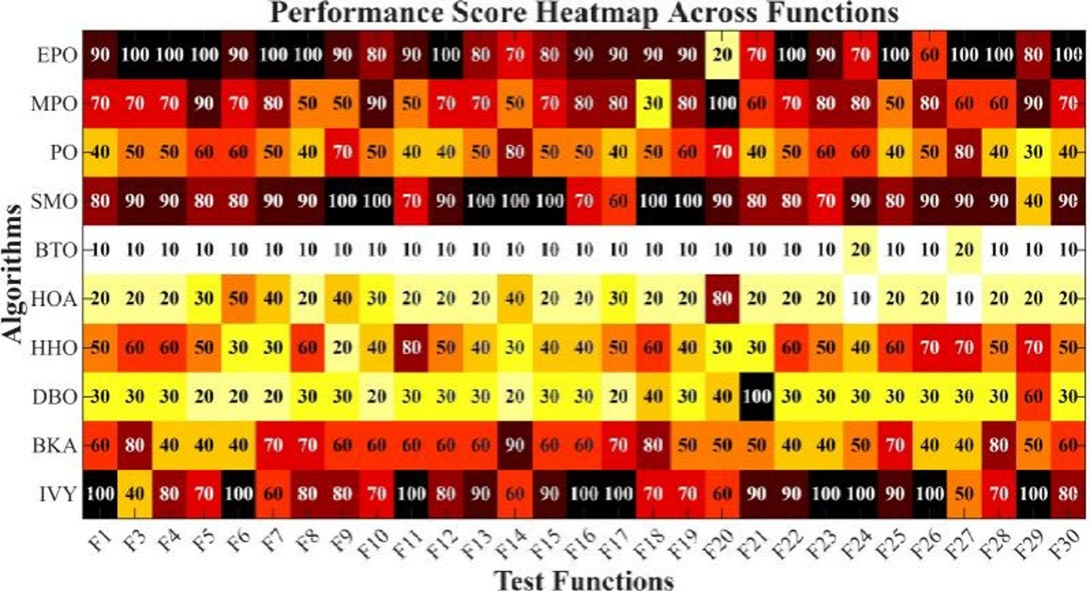
Performance score heatmap across functions.

Figures 6, 7, 8 and 9 show the radar chart, average ranking, comparison of average runtime, and performance score heatmap of the proposed EPO algorithm and comparison algorithms on the CEC2017 benchmark suite (D = 30).

As shown in Figs. 6, 7, 8 and 9, the proposed EPO algorithm achieves optimal performance on most functions., achieves the best overall performance with an average rank of 2.31, securing first place. It exhibits a statistically significant margin over subsequent algorithms, including IVY (average rank: 2.83) and SMO (2.45). The complete ranking order is as follows: EPO > SMO > IVY > MPO > BKA > PO > HHO > DBO > HOA > BTO.The complete ranking order of average runtime is as follows: HOA > DBO > EPO > BKA > MPO > IVY > BTO > PO > HHO > SMO.In summary, EPO achieves a balanced integration of performance and efficiency.

#### Convergence analysis

**Fig. 10:**
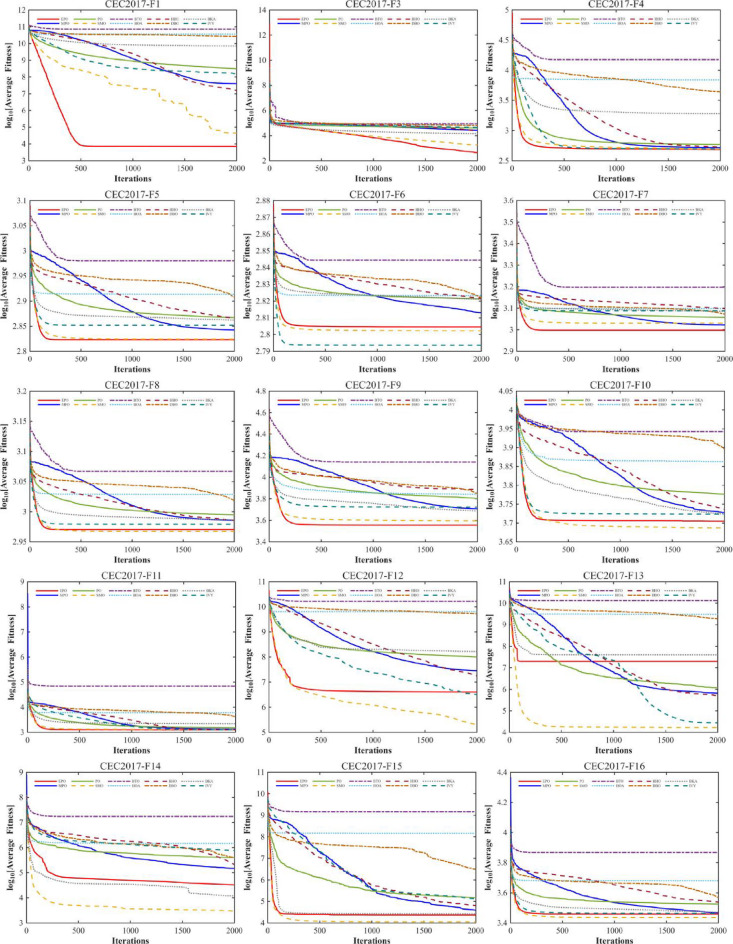

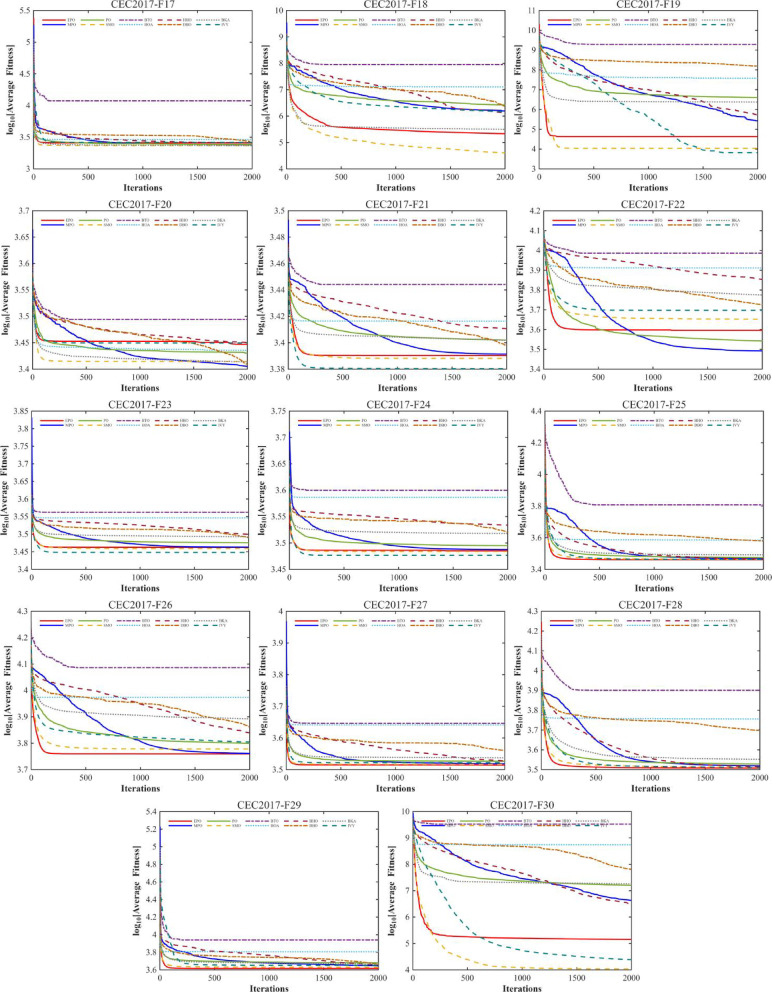
The convergence curves and search history of the proposed technique and other algorithms for IEEE CEC2017 benchmark functions (Dim = 30).

A comprehensive comparative analysis was conducted using the 30-dimensional (D = 30) functions from the CEC2017 benchmark suite. The performance of the proposed algorithm was evaluated against nine state-of-the-art metaheuristics, namely MPO, PO, SMO, BTO, HOA, HHO, DBO, BKA, and IVY. Each algorithm was executed for 30 independent trials, with a population size (Np) of 30 and a maximum of 1,000 iterations (Tmax). The comparative performance is visually represented through convergence curves (Fig. 10).

Experimental results on the CEC2017 30-dimensional benchmark suite (F1–F30, excluding F2),shown in Fig. 10, demonstrate that the proposed Enhanced Particle Optimizer (EPO) exhibits strong overall performance across diverse types of optimization problems. The detailed observations are summarized as follows:

**Unimodal Functions (F1**,** F3). **On F1, EPO rapidly converges to the lowest objective value among all compared algorithms and maintains stability thereafter. On F3, EPO not only achieves a fast initial descent but also continues to improve in the later stages without premature stagnation. These results clearly indicate that EPO possesses high convergence efficiency and strong directional awareness when tackling smooth yet geometrically complex unimodal problems.

**Simple Multimodal Functions (F4-F10). **On F4, F5, F7, and F9, EPO quickly approaches the global optimum neighborhood and achieves the best final solution quality among all algorithms, reflecting its effective ability to escape local optima and maintain population diversity. However, on F8 and F10, although EPO converges rapidly and stably, its final accuracy is slightly inferior to that of SMO, suggesting that SMO’s refined local search strategy is more advantageous in handling highly oscillatory or high-order nonlinear landscapes.

**Hybrid Functions (F11-F20). **On F11, EPO attains the best result among all algorithms, demonstrating its capability to coordinate heterogeneous subcomponents. On F12, EPO stabilizes quickly, but SMO achieves higher final accuracy due to its continued improvement. On F13, EPO’s solution quality is notably worse than that of PO, MPO, and SMO. Similarly, on F14 and F17–F20, EPO converges rapidly and remains stable, yet its final results are generally outperformed by SMO, BKA, PO, or IVY. This suggests that while EPO exhibits high global exploration efficiency in handling strongly coupled, multi-module hybrid problems, its local exploitation depth still requires further enhancement.

**Composition Functions (F21-F30). **As the most challenging class, composition functions involve weighted combinations of multiple base functions with varying scales, rotations, and complex basin structures. Within this category, EPO performs exceptionally well on F25–F29, achieving both the fastest convergence and the highest solution accuracy among all algorithms. However, on F21, F22, F23, F24, and F30, EPO stabilizes quickly but yields lower final accuracy compared to SMO or IVY. Notably, as an improved variant of the original PO algorithm, EPO significantly outperforms both the baseline PO and another enhanced version (MPO) on the majority of test functions—particularly on unimodal and certain composition functions—thereby validating the effectiveness of the proposed enhancements.Overall, EPO strikes a good balance between convergence efficiency, stability, and generalization ability.

In summary, although EPO exhibits slightly lower final accuracy than SMO and a few other advanced algorithms on some hybrid and composition functions, it demonstrates clear advantages in terms of convergence speed, stability, and robustness. Compared to the original PO algorithm, EPO achieves significant improvements in both convergence efficiency and solution quality, highlighting its practical utility and generalization capability.

#### Analysis of box plot results

**Fig. 11:**
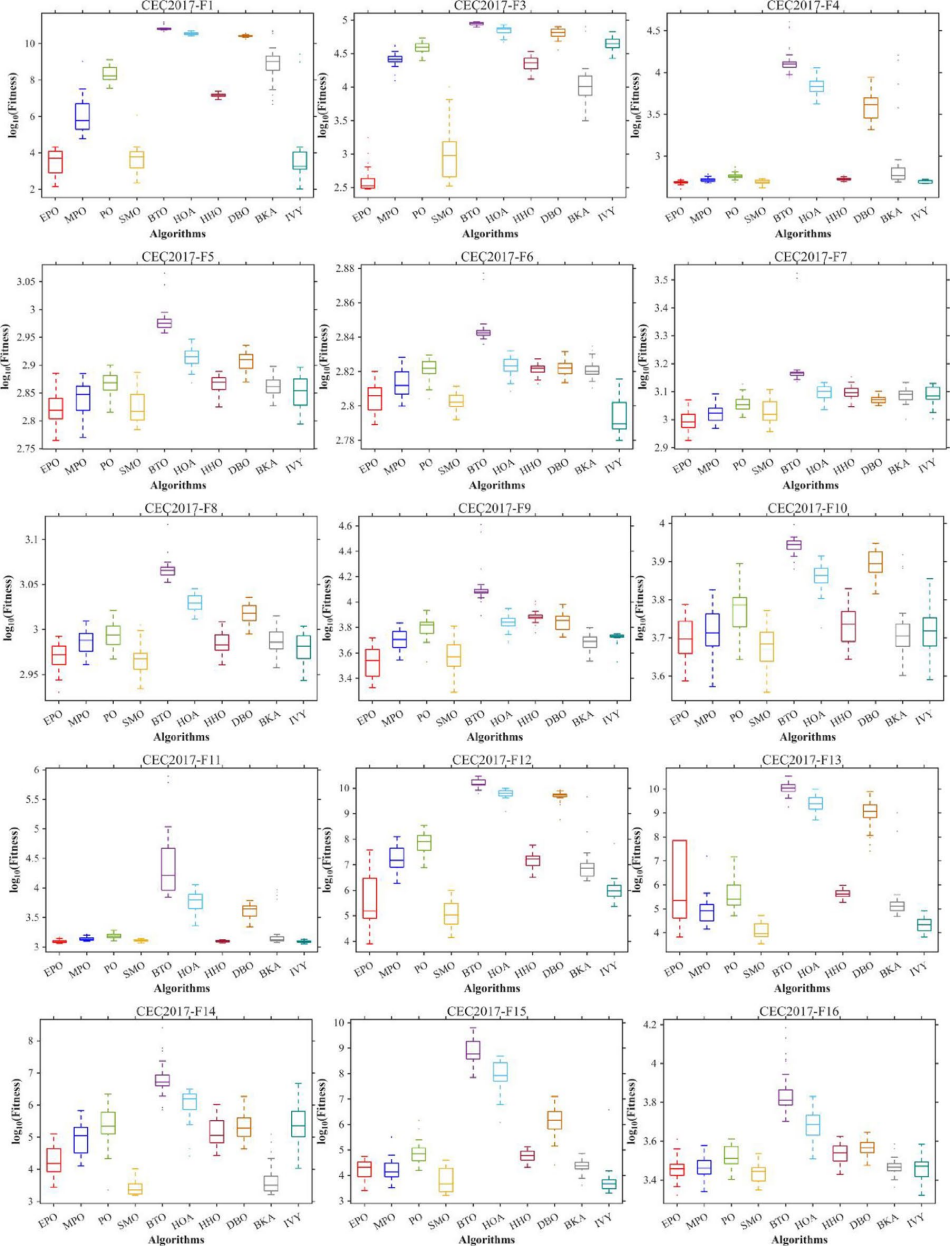

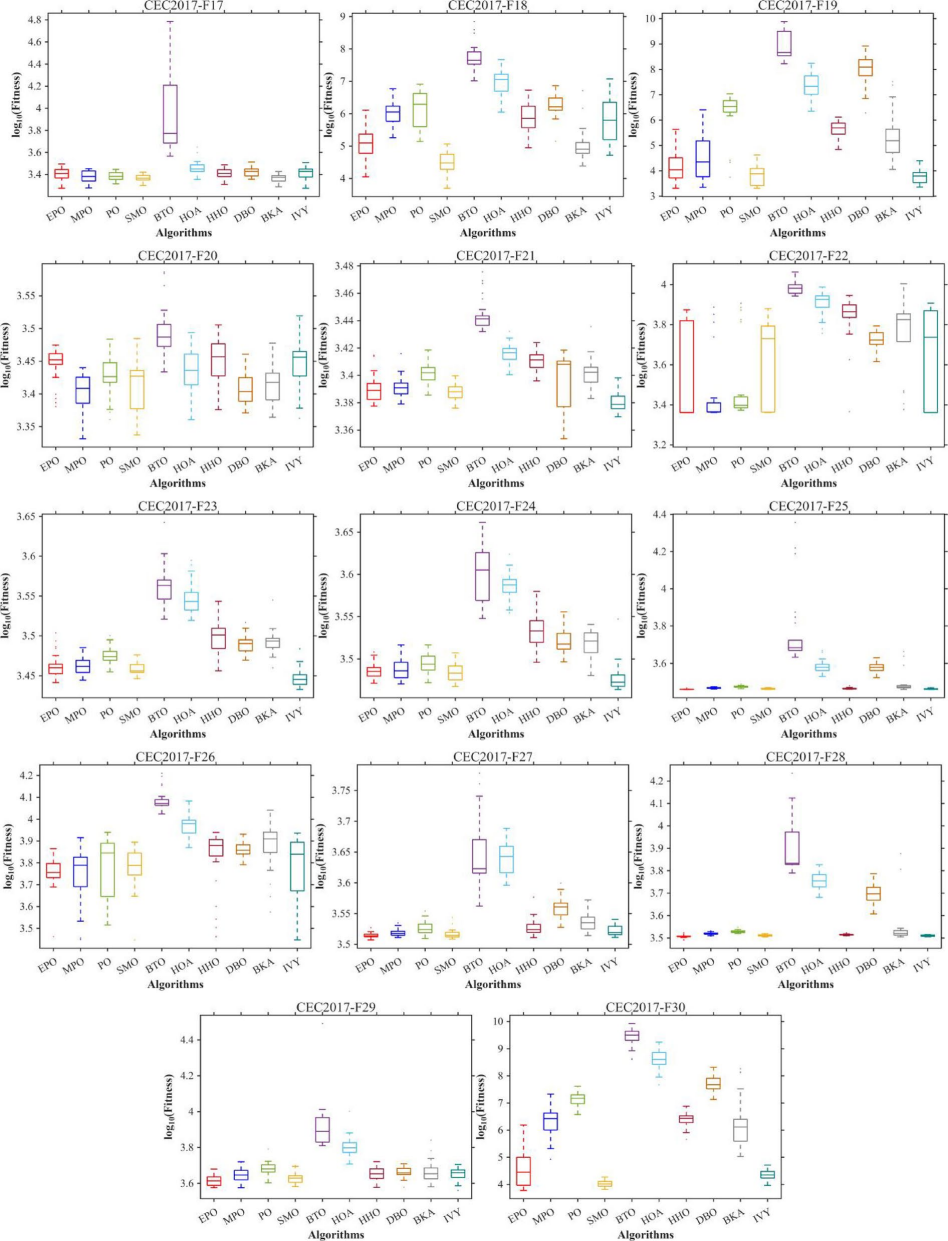
Boxplots for the proposed technique and other algorithms for IEEE CEC2017 benchmark functions (Dim = 30).

A box plot is a standardized method for graphically depicting numerical data through a five-number summary. This summary consists of five key statistical values: the minimum, the first quartile (Q1 or 25th percentile), the median (Q2), the third quartile (Q3 or 75th percentile), and the maximum. Figure 11 presents the resulting box plots from 30 independent runs of the algorithm on the 30-dimensional CEC2017 benchmark functions.The box plots graphically illustrate the performance distribution of EPO relative to the canonical PO algorithm and other benchmark metaheuristics algorithms.

As shown in Fig. 11, the EPO algorithm demonstrates outstanding performance across multiple metrics. Specifically, EPO achieves the lowest median error among all compared algorithms on 11 functions (F3, F4, F7, F9, F11, F22, and F25–F29). It also attains the smallest interquartile range (IQR) on 6 functions (F4, F20, F24, F25, F27, and F28), indicating high consistency and stability. Furthermore, EPO exhibits zero outliers on 16 functions, such as F1, F5–F7, F9–F15, F17–F19, F22, F29, and F30, demonstrating its robustness.

Notably, when compared directly with the PO algorithm, EPO consistently outperforms PO by achieving lower median errors on all 30 test functions, realizing smaller IQRs on 10 functions (F4, F11, F16, F18, F20, F24, F25, F26, F27, and F28), and maintaining zero outliers on 18 functions.

In summary, EPO not only leads in overall benchmark performance but also significantly surpasses PO in terms of accuracy, stability, and robustness. These results validate the effectiveness of its design mechanisms and highlight its substantial potential for practical applications.

#### Analysis of Wilcoxon rank sum test results

**Fig. 12:**
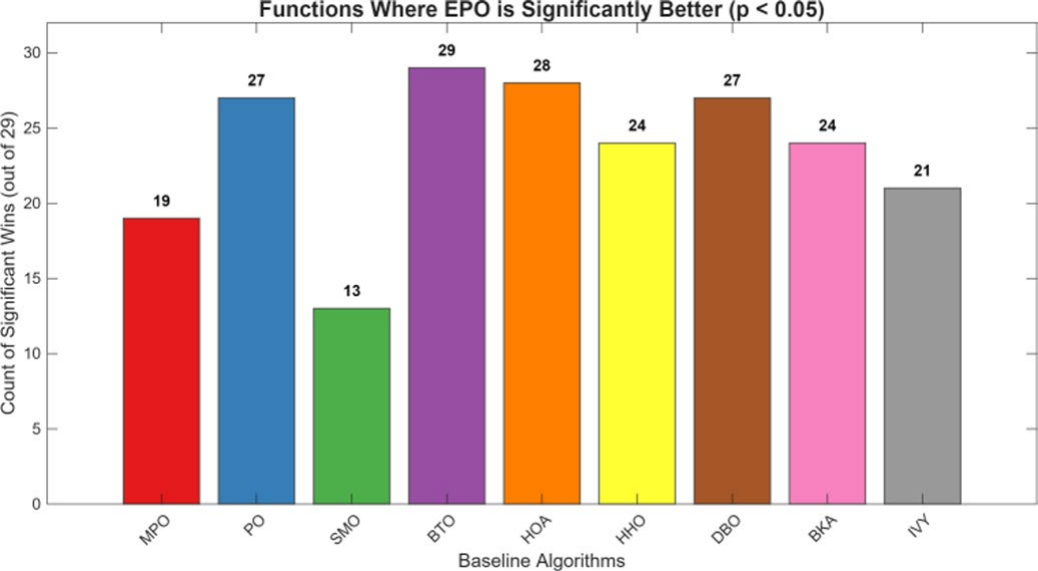
Boxplots for the proposed technique and other algorithms for IEEE CEC2017 benchmark functions (Dim = 30).

To rigorously evaluate the performance advantages of the proposed EPO algorithm, this study employs the Wilcoxon rank-sum test to conduct pairwise comparisons with nine state-of-the-art optimization algorithms (MPO, PO, SMO, BTO, HOA, HHO, DBO, BKA, and IVY) across 29 benchmark functions at a significance level of α = 0.05. The results of the Wilcoxon rank-sum test are presented in Table 5 and illustrated in Fig. 12.

**Table 5:** Wilcoxon rank sum test results.

Function	MPO	PO	SMO	BTO	HOA	HHO	DBO	BKA	IVY
F1	3.0199E-11	3.0199E-11	**5.0114E-01**	3.0199E-11	3.0199E-11	3.0199E-11	3.0199E-11	3.0199E-11	**6.5204E-01**
F3	3.0199E-11	3.0199E-11	1.5964E-07	3.0199E-11	3.0199E-11	3.0199E-11	3.0199E-11	3.0199E-11	3.0199E-11
F4	2.4913E-06	2.1544E-10	**3.1830E-01**	3.0199E-11	3.0199E-11	5.4617E-09	3.0199E-11	4.6159E-10	**2.5188E-01**
F5	5.3221E-03	1.3594E-07	**8.7663E-01**	3.0199E-11	4.0772E-11	5.5329E-08	3.6897E-11	2.1959E-07	4.9818E-04
F6	6.2027E-04	2.9215E-09	**2.5805E-01**	3.0199E-11	1.4643E-10	1.2057E-10	1.9568E-10	5.5727E-10	4.9426E-05
F7	5.0842E-03	7.6950E-08	3.0339E-03	3.0199E-11	6.0658E-11	5.4941E-11	6.1210E-10	4.6159E-10	6.1210E-10
F8	2.6806E-04	7.6950E-08	**2.0621E-01**	3.0199E-11	3.0199E-11	3.3679E-04	3.0199E-11	9.7917E-05	3.6439E-02
F9	2.4913E-06	3.8202E-10	**1.9073E-01**	3.0199E-11	9.9186E-11	3.0199E-11	3.0199E-11	1.2493E-05	1.2870E-09
F10	**1.9073E-01**	2.4327E-05	**2.1702E-01**	3.0199E-11	8.1527E-11	**5.0120E-02**	3.0199E-11	**7.2827E-01**	**3.1119E-01**
F11	6.5261E-07	2.3715E-10	1.1738E-03	3.0199E-11	3.0199E-11	**7.0127E-02**	3.0199E-11	2.9590E-05	**6.1001E-01**
F12	1.0666E-07	1.2057E-10	**6.1452E-02**	3.0199E-11	3.0199E-11	2.0283E-07	3.0199E-11	1.3367E-05	1.0763E-02
F13	**5.1877E-02**	**9.5873E-01**	7.6950E-08	3.0199E-11	3.0199E-11	**7.3940E-01**	3.1589E-10	**6.7350E-01**	5.4620E-06
F14	6.2828E-06	1.6980E-08	8.8910E-10	3.0199E-11	1.9568E-10	9.8329E-08	2.0338E-09	8.2919E-06	6.5277E-08
F15	**7.7312E-01**	1.2541E-07	1.0576E-03	3.0199E-11	3.0199E-11	1.4733E-07	1.0937E-10	**3.7108E-01**	2.4327E-05
F16	**5.1060E-01**	8.1465E-05	**9.9258E-02**	3.0199E-11	7.3891E-11	3.0939E-06	2.9215E-09	**3.1119E-01**	**5.0114E-01**
F17	**5.5546E-02**	**5.3685E-02**	8.1200E-04	3.0199E-11	2.3800E-03	**9.3519E-01**	**2.7071E-01**	5.8737E-04	**4.9178E-01**
F18	2.4386E-09	1.0105E-08	9.5332E-07	3.0199E-11	3.6897E-11	9.0632E-08	3.8202E-10	**1.8577E-01**	2.1327E-05
F19	**1.5798E-01**	1.2870E-09	**7.0127E-02**	3.0010E-11	3.0199E-11	3.8202E-10	3.0199E-11	3.0103E-07	2.3800E-03
F20	1.4733E-07	7.6171E-03	4.4592E-04	2.0283E-07	**1.1199E-01**	**4.1191E-01**	1.3853E-06	7.2208E-06	**7.8446E-01**
F21	**3.2553E-01**	4.4205E-06	**6.6273E-01**	3.0199E-11	4.6159E-10	7.7725E-09	3.2651E-02	4.6390E-05	3.8307E-05
F22	3.1463E-02	1.1227E-02	2.1504E-02	3.0180E-11	1.1731E-09	8.3486E-08	**6.7865E-02**	1.3247E-04	8.3137E-03
F23	**5.5923E-01**	4.6390E-05	**5.2014E-01**	3.0199E-11	3.0199E-11	1.1023E-08	3.0811E-08	1.8500E-08	2.2780E-05
F24	**5.1060E-01**	4.9818E-04	**4.7335E-01**	3.0199E-11	3.0199E-11	5.4941E-11	7.3891E-11	1.6947E-09	5.6073E-05
F25	6.1210E-10	4.0772E-11	3.9881E-04	3.0199E-11	3.0199E-11	2.3897E-08	3.0199E-11	5.0723E-10	3.7782E-02
F26	**2.9047E-01**	4.6756E-02	**6.5671E-02**	3.0199E-11	3.0199E-11	7.6588E-05	1.0702E-09	4.4440E-07	6.3772E-03
F27	3.5012E-03	8.8411E-07	**4.0354E-01**	3.0199E-11	3.0199E-11	9.0632E-08	3.0199E-11	1.0702E-09	1.1747E-04
F28	3.8249E-09	5.4941E-11	7.2951E-04	3.0199E-11	3.0199E-11	6.5261E-07	3.0199E-11	1.3594E-07	2.9205E-02
F29	6.2027E-04	4.3106E-08	**8.2357E-02**	3.0199E-11	3.0199E-11	5.6073E-05	3.0103E-07	3.1821E-04	6.3560E-05
F30	5.0723E-10	3.0199E-11	6.5486E-04	3.0199E-11	3.0199E-11	5.4941E-11	3.0199E-11	2.6695E-09	**4.8252E-01**

The results presented in Table 5; Fig. 12 indicate that, across a total of 261 pairwise Wilcoxon rank-sum tests (29 functions×9 algorithms), the proposed EPO algorithm achieves statistically significantly better performance than its competitors in 212 cases (81.2%) at the significance level of α = 0.05. Specifically, EPO demonstrates a win rate exceeding 93% against PO, BTO, HOA, and DBO. Notably, it attains significant superiority over BTO on all 29 functions (100% win rate). In contrast, MPO, SMO, and IVY exhibit relatively stronger competitiveness, showing no statistically significant difference from EPO on at least 25% of the test functions. Nevertheless, EPO still outperforms these three algorithms in the majority of comparisons and remains statistically superior overall.

In summary, EPO not only exhibits highly significant performance advantages in the vast majority of test scenarios but also demonstrates exceptional robustness across a diverse range of optimization problems.

### Performance analysis on 50-dimensional functions

#### Statistics analysis

To evaluate performance, a comprehensive analysis was conducted using the 30-dimensional (D = 50) functions of the CEC2017 benchmark suite. The proposed algorithm was benchmarked against nine state-of-the-art metaheuristics, namely MPO, PO, SMO, BTO, HOA, HHO, DBO, BKA, and IVY. For all experiments, the population size (Np) was set to 30, the maximum number of iterations (Tmax) was 2,000, and each algorithm was executed for 30 independent runs. Performance was quantified using five statistical indicators: minimum (Min), mean (Avg), median (Median), worst (Worst), and standard deviation (Std). These results are presented in Table 6.

**Table 6:** The statistical results of benchmark functions using the proposed technique and other algorithms (Dim = 30).

Function	Item	EPO	MPO	PO	SMO	BTO	HOA	HHO	DBO	BKA	IVY
F1	min	**1.4942E + 02**	6.5468E + 06	5.4116E + 08	1.0200E + 06	9.5514E + 10	5.8788E + 10	6.1544E + 07	5.7427E + 10	6.1015E + 08	1.7355E + 02
F1	std	**5.8235E + 03**	2.2231E + 08	1.2280E + 09	8.1132E + 08	4.5816E + 10	1.0527E + 10	1.3011E + 07	5.4466E + 09	2.2429E + 10	2.5242E + 07
F1	avg	**4.8720E + 03**	1.0268E + 08	1.9436E + 09	2.8734E + 08	1.2810E + 11	8.7276E + 10	9.0746E + 07	6.8350E + 10	1.7631E + 10	4.6126E + 06
F1	avg_time	5.01E-01	6.82E-01	7.73E-01	5.2467E + 00	1.2370E + 00	**2.32E-01**	6.04E-01	3.53E-01	4.45E-01	8.28E-01
F3	min	1.7591E + 04	7.2364E + 04	9.7810E + 04	**1.5522E + 04**	1.6310E + 05	1.0690E + 05	4.9676E + 04	1.3399E + 05	2.7640E + 04	1.2710E + 05
F3	std	2.9138E + 04	2.1130E + 04	**1.3846E + 04**	1.3909E + 04	5.7829E + 07	1.5916E + 04	1.7945E + 04	2.1775E + 04	4.8934E + 04	8.3227E + 04
F3	avg	5.1022E + 04	1.1655E + 05	1.3035E + 05	**3.2495E + 04**	1.3957E + 07	1.5401E + 05	8.4070E + 04	1.7334E + 05	6.6182E + 04	2.6604E + 05
F3	avg_time	5.15E-01	6.61E-01	7.47E-01	5.1234E + 00	1.2231E + 00	**2.29E-01**	6.02E-01	3.62E-01	4.44E-01	8.31E-01
F4	min	4.4193E + 02	6.1909E + 02	7.2327E + 02	5.4674E + 02	3.1108E + 04	1.8029E + 04	5.8502E + 02	1.1481E + 04	6.3995E + 02	**4.3288E + 02**
F4	std	1.0566E + 02	1.1717E + 02	1.5210E + 02	6.4372E + 01	2.2766E + 04	3.6468E + 03	**5.0975E + 01**	1.4452E + 03	4.9481E + 03	5.1669E + 01
F4	avg	**5.4904E + 02**	7.3130E + 02	9.8870E + 02	6.7338E + 02	4.2512E + 04	2.3463E + 04	6.8784E + 02	1.4873E + 04	2.6031E + 03	5.6144E + 02
F4	avg_time	4.97E-01	6.73E-01	7.54E-01	5.2068E + 00	1.2250E + 00	**2.31E-01**	5.91E-01	3.46E-01	4.54E-01	8.45E-01
F5	min	7.5769E + 02	7.3706E + 02	8.2708E + 02	**7.2892E + 02**	1.1883E + 03	9.9251E + 02	8.4570E + 02	1.0046E + 03	8.3565E + 02	7.9451E + 02
F5	std	4.9426E + 01	4.3400E + 01	4.5897E + 01	5.6244E + 01	8.6259E + 01	4.0082E + 01	2.8007E + 01	**2.4228E + 01**	7.5652E + 01	3.3667E + 01
F5	avg	8.4208E + 02	8.5802E + 02	9.2162E + 02	**8.1536E + 02**	1.2285E + 03	1.0731E + 03	9.0135E + 02	1.0681E + 03	9.0621E + 02	8.5772E + 02
F5	avg_time	6.85E-01	9.50E-01	1.0213E + 00	5.3261E + 00	1.3559E + 00	**3.66E-01**	9.45E-01	4.77E-01	7.22E-01	9.85E-01
F6	min	6.3089E + 02	6.4701E + 02	6.6467E + 02	6.3330E + 02	6.9824E + 02	6.6744E + 02	6.6329E + 02	6.7378E + 02	6.5765E + 02	**6.0787E + 02**
F6	std	8.0414E + 00	7.2609E + 00	5.3971E + 00	9.1278E + 00	1.4548E + 01	6.6209E + 00	4.2882E + 00	**3.8223E + 00**	8.9581E + 00	1.6866E + 01
F6	avg	6.5037E + 02	6.6239E + 02	6.7501E + 02	6.4785E + 02	7.1355E + 02	6.8330E + 02	6.7292E + 02	6.8151E + 02	6.7073E + 02	**6.3471E + 02**
F6	avg_time	1.1003E + 00	1.6130E + 00	1.6748E + 00	5.6912E + 00	1.6367E + 00	**6.97E-01**	1.7411E + 00	8.17E-01	1.3616E + 00	1.3382E + 00
F7	min	**1.1205E + 03**	1.1996E + 03	1.2985E + 03	1.2862E + 03	1.9677E + 03	1.6478E + 03	1.6022E + 03	1.5901E + 03	1.4911E + 03	1.3585E + 03
F7	std	1.5231E + 02	1.1338E + 02	1.4520E + 02	2.0764E + 02	7.8034E + 02	8.1146E + 01	9.1192E + 01	**6.0063E + 01**	9.1585E + 01	1.1365E + 02
F7	avg	**1.4054E + 03**	1.4347E + 03	1.6657E + 03	1.6854E + 03	2.2690E + 03	1.8136E + 03	1.8256E + 03	1.7334E + 03	1.7137E + 03	1.6819E + 03
F7	avg_time	6.90E-01	9.62E-01	1.0423E + 00	5.3790E + 00	1.3604E + 00	**3.77E-01**	9.58E-01	4.78E-01	7.40E-01	1.00E+00
F8	min	**1.0129E + 03**	1.0194E + 03	1.1820E + 03	1.0142E + 03	1.4793E + 03	1.3395E + 03	1.1439E + 03	1.2821E + 03	1.1317E + 03	1.0846E + 03
F8	std	5.0665E + 01	5.9896E + 01	3.7950E + 01	4.2904E + 01	7.9266E + 01	3.6330E + 01	**3.5741E + 01**	3.6478E + 01	9.1083E + 01	3.7385E + 01
F8	avg	1.1171E + 03	1.1611E + 03	1.2460E + 03	**1.1078E + 03**	1.5626E + 03	1.4130E + 03	1.1943E + 03	1.3500E + 03	1.2311E + 03	1.1726E + 03
F8	avg_time	6.80E-01	9.54E-01	1.0299E + 00	5.3431E + 00	1.3566E + 00	**3.75E-01**	9.55E-01	4.85E-01	7.27E-01	9.78E-01
F9	min	**5.5818E + 03**	1.1207E + 04	1.6045E + 04	5.9558E + 03	3.5215E + 04	1.9935E + 04	2.1645E + 04	2.2128E + 04	1.1787E + 04	1.0817E + 04
F9	std	1.7974E + 03	2.3174E + 03	2.1725E + 03	2.7155E + 03	1.9954E + 04	3.0044E + 03	3.4017E + 03	3.2254E + 03	4.6911E + 03	**7.2331E + 02**
F9	avg	**9.4322E + 03**	1.4920E + 04	2.0476E + 04	1.1380E + 04	4.6433E + 04	2.6396E + 04	2.6361E + 04	2.8550E + 04	1.5711E + 04	1.3048E + 04
F9	avg_time	6.74E-01	9.52E-01	1.0252E + 00	5.3649E + 00	1.3546E + 00	**3.70E-01**	9.66E-01	4.92E-01	7.24E-01	9.76E-01
F10	min	**5.2939E + 03**	6.4739E + 03	7.3661E + 03	6.0212E + 03	1.4809E + 04	1.1322E + 04	7.5675E + 03	1.0993E + 04	6.2120E + 03	6.2845E + 03
F10	std	1.0236E + 03	1.2326E + 03	1.3678E + 03	8.5830E + 02	**6.5163E + 02**	8.5031E + 02	1.0801E + 03	1.0351E + 03	1.5768E + 03	9.1665E + 02
F10	avg	**7.3840E + 03**	9.1825E + 03	1.0505E + 04	8.1249E + 03	1.5653E + 04	1.3025E + 04	9.0807E + 03	1.3671E + 04	9.0475E + 03	8.2987E + 03
F10	avg_time	7.96E-01	1.1218E + 00	1.2048E + 00	5.4356E + 00	1.4148E + 00	**4.47E-01**	1.1596E + 00	6.06E-01	8.91E-01	1.0655E + 00
F11	min	1.2244E + 03	1.4982E + 03	2.0130E + 03	1.3142E + 03	2.0418E + 04	1.1889E + 04	1.3747E + 03	8.8958E + 03	1.3989E + 03	**1.2241E + 03**
F11	std	7.6658E + 01	2.6681E + 02	5.4647E + 02	1.8473E + 02	2.6132E + 04	2.9791E + 03	**7.4717E + 01**	1.5931E + 03	5.3439E + 03	4.1690E + 02
F11	avg	**1.3842E + 03**	1.8597E + 03	2.7326E + 03	1.5085E + 03	3.5247E + 04	1.8142E + 04	1.5163E + 03	1.1961E + 04	4.0247E + 03	1.7599E + 03
F11	avg_time	5.73E-01	7.86E-01	8.63E-01	5.2706E + 00	1.2696E + 00	**2.88E-01**	7.69E-01	4.14E-01	5.61E-01	9.02E-01
F12	min	**4.4874E + 05**	4.1545E + 07	8.6449E + 07	4.6402E + 05	5.2064E + 10	2.8789E + 10	2.6218E + 07	1.8723E + 10	1.2676E + 07	8.6687E + 05
F12	std	1.9938E + 07	1.2099E + 08	3.1503E + 08	**8.1644E + 06**	1.8854E + 10	1.0600E + 10	5.4334E + 07	9.3273E + 09	1.6062E + 10	7.0520E + 07
F12	avg	1.1111E + 07	1.8851E + 08	6.5028E + 08	**8.1990E + 06**	8.4595E + 10	4.9609E + 10	8.8598E + 07	3.1323E + 10	6.8942E + 09	1.9937E + 07
F12	avg_time	6.54E-01	9.30E-01	1.0122E + 00	5.3640E + 00	1.3386E + 00	**3.58E-01**	9.02E-01	4.87E-01	6.98E-01	9.56E-01
F13	min	1.6160E + 04	3.0211E + 04	1.2505E + 06	**5.2739E + 03**	3.8135E + 10	6.7970E + 09	1.5095E + 06	1.7364E + 09	5.5549E + 05	6.9194E + 03
F13	std	4.7012E + 08	3.3931E + 07	4.4264E + 07	**1.1940E + 04**	1.6686E + 10	8.7839E + 09	2.0822E + 06	6.0647E + 09	4.0010E + 09	2.8972E + 07
F13	avg	8.5889E + 07	6.5779E + 06	3.9558E + 07	**1.7151E + 04**	5.7450E + 10	2.4226E + 10	3.7095E + 06	1.0546E + 10	7.3827E + 08	5.3705E + 06
F13	avg_time	5.95E-01	7.87E-01	8.77E-01	5.3030E + 00	1.2660E + 00	**2.93E-01**	7.55E-01	4.19E-01	5.69E-01	9.06E-01
F14	min	7.9519E + 03	2.0185E + 05	2.8659E + 05	**3.6287E + 03**	2.4350E + 07	1.7861E + 07	1.8029E + 05	6.5874E + 05	4.4750E + 03	7.4673E + 04
F14	std	1.7009E + 05	8.0506E + 05	1.4422E + 06	**3.7040E + 04**	1.1293E + 08	2.6574E + 07	6.5861E + 05	5.7989E + 06	6.3426E + 06	1.1368E + 06
F14	avg	1.8768E + 05	9.9644E + 05	2.3193E + 06	**3.5761E + 04**	1.1848E + 08	5.5262E + 07	1.1454E + 06	6.4232E + 06	2.1867E + 06	1.2263E + 06
F14	avg_time	7.57E-01	1.0754E + 00	1.1638E + 00	5.3747E + 00	1.4197E + 00	**4.39E-01**	1.1158E + 00	5.73E-01	8.72E-01	1.0631E + 00
F15	min	5.8308E + 03	6.0207E + 03	5.8849E + 04	**1.9974E + 03**	5.4146E + 09	2.7772E + 08	1.8607E + 05	9.8758E + 07	3.8739E + 04	2.8613E + 03
F15	std	1.3923E + 04	3.5022E + 04	9.9969E + 06	**7.3413E + 03**	8.1314E + 09	2.2699E + 09	2.1556E + 05	1.0211E + 09	8.8716E + 08	3.2437E + 04
F15	avg	2.6739E + 04	4.5627E + 04	2.5681E + 06	**9.5715E + 03**	1.4541E + 10	4.2902E + 09	5.7969E + 05	1.2627E + 09	2.7021E + 08	2.0125E + 04
F15	avg_time	5.50E-01	7.60E-01	8.35E-01	5.2579E + 00	1.2561E + 00	**2.75E-01**	7.07E-01	3.93E-01	5.27E-01	8.70E-01
F16	min	2.9661E + 03	3.2554E + 03	3.7216E + 03	2.9373E + 03	6.7653E + 03	5.5663E + 03	2.7955E + 03	4.2266E + 03	2.9377E + 03	**2.5470E + 03**
F16	std	4.3311E + 02	4.4304E + 02	6.9695E + 02	**4.0099E + 02**	2.3724E + 03	1.0636E + 03	6.2288E + 02	5.0649E + 02	1.2757E + 03	5.1903E + 02
F16	avg	3.8504E + 03	4.3168E + 03	4.7553E + 03	**3.5454E + 03**	1.1380E + 04	7.3112E + 03	4.1415E + 03	5.2532E + 03	4.2752E + 03	3.6457E + 03
F16	avg_time	6.77E-01	8.69E-01	9.55E-01	5.2942E + 00	1.3053E + 00	**3.34E-01**	8.53E-01	4.61E-01	6.53E-01	9.39E-01
F17	min	2.6672E + 03	2.7762E + 03	2.8683E + 03	**2.3939E + 03**	6.4098E + 03	3.5243E + 03	2.6637E + 03	3.8184E + 03	2.9371E + 03	2.6957E + 03
F17	std	4.1685E + 02	**3.5455E + 02**	4.8806E + 02	4.1798E + 02	1.1328E + 05	7.2420E + 02	4.0304E + 02	4.4666E + 02	5.1187E + 02	4.2977E + 02
F17	avg	3.5139E + 03	3.5914E + 03	3.9840E + 03	**3.2843E + 03**	5.3388E + 04	4.8659E + 03	3.7665E + 03	4.5256E + 03	3.7059E + 03	3.6086E + 03
F17	avg_time	9.35E-01	1.3471E + 00	1.4203E + 00	5.6418E + 00	1.5416E + 00	**5.63E-01**	1.4293E + 00	6.88E-01	1.1183E + 00	1.1866E + 00
F18	min	9.7687E + 04	3.2347E + 05	4.6397E + 05	**3.4571E + 04**	9.4292E + 07	1.9060E + 07	2.9985E + 05	1.3478E + 06	6.0467E + 04	3.6372E + 05
F18	std	3.9091E + 05	3.3125E + 06	7.6513E + 06	**1.9769E + 05**	2.7721E + 08	4.7927E + 07	3.2975E + 06	8.2809E + 06	1.8807E + 07	9.9778E + 05
F18	avg	4.7385E + 05	5.1086E + 06	1.0973E + 07	**2.2381E + 05**	4.1343E + 08	9.6086E + 07	4.2808E + 06	9.1338E + 06	7.9146E + 06	1.7592E + 06
F18	avg_time	6.19E-01	8.55E-01	9.40E-01	5.2176E + 00	1.3262E + 00	**3.30E-01**	8.61E-01	4.60E-01	6.44E-01	9.38E-01
F19	min	**2.3162E + 03**	3.0775E + 03	8.4695E + 04	3.7536E + 03	1.5102E + 09	1.1318E + 08	1.9621E + 05	1.0480E + 08	1.3265E + 05	6.4628E + 03
F19	std	4.7523E + 04	1.7992E + 06	6.1890E + 06	**1.1396E + 04**	4.2003E + 09	7.1857E + 08	7.6124E + 05	5.6158E + 08	2.3848E + 08	1.5892E + 08
F19	avg	3.4197E + 04	6.5483E + 05	6.9644E + 06	**1.7076E + 04**	6.1649E + 09	1.2259E + 09	1.1245E + 06	5.9359E + 08	6.0015E + 07	4.4122E + 07
F19	avg_time	2.9223E + 00	4.6177E + 00	4.6307E + 00	7.3064E + 00	3.0430E + 00	**2.1670E + 00**	5.3171E + 00	2.3012E + 00	4.3393E + 00	2.8366E + 00
F20	min	**2.6645E + 03**	2.7029E + 03	2.8814E + 03	2.6674E + 03	3.9428E + 03	3.1037E + 03	2.8219E + 03	3.0526E + 03	2.7219E + 03	2.9141E + 03
F20	std	3.9071E + 02	3.3444E + 02	3.5743E + 02	3.1683E + 02	3.8074E + 02	2.8870E + 02	3.0450E + 02	3.1706E + 02	**2.5476E + 02**	3.2861E + 02
F20	avg	3.4233E + 03	3.3294E + 03	3.5793E + 03	3.2593E + 03	4.4078E + 03	3.5686E + 03	3.4207E + 03	3.6066E + 03	**3.1298E + 03**	3.4836E + 03
F20	avg_time	9.86E-01	1.4360E + 00	1.5057E + 00	5.6328E + 00	1.5834E + 00	**6.08E-01**	1.5546E + 00	7.42E-01	1.1924E + 00	1.2423E + 00
F21	min	2.4514E + 03	2.5802E + 03	2.6450E + 03	2.5333E + 03	3.1413E + 03	2.8649E + 03	2.7248E + 03	2.8320E + 03	2.6789E + 03	**2.4075E + 03**
F21	std	7.0221E + 01	7.6214E + 01	5.8497E + 01	5.6937E + 01	1.8013E + 02	6.0382E + 01	6.5962E + 01	**4.4654E + 01**	1.0250E + 02	8.4979E + 01
F21	avg	2.5999E + 03	2.7068E + 03	2.7922E + 03	2.6457E + 03	3.3095E + 03	2.9751E + 03	2.8447E + 03	2.9293E + 03	2.8244E + 03	**2.5506E + 03**
F21	avg_time	1.2125E + 00	1.8267E + 00	1.8732E + 00	5.8312E + 00	1.7542E + 00	**#######**	1.9444E + 00	9.14E-01	1.5552E + 00	1.4087E + 00
F22	min	8.6293E + 03	8.8475E + 03	**3.3288E + 03**	8.4670E + 03	1.5959E + 04	1.3179E + 04	9.5266E + 03	9.1480E + 03	8.8333E + 03	8.0909E + 03
F22	std	9.4459E + 02	1.0925E + 03	1.9850E + 03	8.9676E + 02	**6.3839E + 02**	6.9331E + 02	1.2404E + 03	1.8040E + 03	1.6265E + 03	1.0255E + 03
F22	avg	1.0342E + 04	1.0941E + 04	1.1853E + 04	**1.0082E + 04**	1.7342E + 04	1.4901E + 04	1.1423E + 04	1.4995E + 04	1.0602E + 04	1.0286E + 04
F22	avg_time	1.4110E + 00	2.1211E + 00	2.1665E + 00	5.9482E + 00	1.8696E + 00	**9.21E-01**	2.3457E + 00	1.0836E + 00	1.8536E + 00	1.5604E + 00
F23	min	2.9776E + 03	3.1128E + 03	3.2660E + 03	3.0835E + 03	4.0829E + 03	4.0278E + 03	3.4242E + 03	3.5503E + 03	3.4272E + 03	**2.9154E + 03**
F23	std	2.0709E + 02	**1.0765E + 02**	1.4016E + 02	1.0948E + 02	4.1983E + 02	2.3331E + 02	1.8102E + 02	1.1183E + 02	2.0734E + 02	1.3891E + 02
F23	avg	3.2593E + 03	3.3612E + 03	3.5530E + 03	3.2735E + 03	4.7656E + 03	4.5157E + 03	3.8066E + 03	3.7482E + 03	3.7176E + 03	**3.0870E + 03**
F23	avg_time	1.5284E + 00	2.2986E + 00	2.3479E + 00	6.0458E + 00	1.9971E + 00	**1.0096E + 00**	2.5050E + 00	1.1644E + 00	2.0183E + 00	1.6581E + 00
F24	min	3.1460E + 03	3.1282E + 03	3.3657E + 03	3.1716E + 03	4.2729E + 03	4.6705E + 03	3.7459E + 03	3.6415E + 03	3.4782E + 03	**3.0157E + 03**
F24	std	1.8914E + 02	1.6180E + 02	1.7368E + 02	**1.3016E + 02**	5.1313E + 02	2.0595E + 02	2.5717E + 02	2.0996E + 02	1.7437E + 02	2.2679E + 02
F24	avg	3.5058E + 03	3.4670E + 03	3.6601E + 03	3.4136E + 03	5.0213E + 03	4.9804E + 03	4.2694E + 03	4.0429E + 03	3.7878E + 03	**3.2755E + 03**
F24	avg_time	1.8592E + 00	2.5780E + 00	2.6442E + 00	6.2364E + 00	2.1279E + 00	**1.1683E + 00**	2.8558E + 00	1.2630E + 00	2.2425E + 00	1.7460E + 00
F25	min	**2.9312E + 03**	3.0894E + 03	3.1824E + 03	3.0842E + 03	1.2799E + 04	8.0306E + 03	3.0964E + 03	7.9888E + 03	3.1319E + 03	3.0437E + 03
F25	std	3.8544E + 01	8.9149E + 01	1.4309E + 02	4.7372E + 01	1.2985E + 04	1.2698E + 03	3.0754E + 01	8.4591E + 02	2.8412E + 03	**1.9413E + 01**
F25	avg	**3.0502E + 03**	3.2001E + 03	3.4233E + 03	3.1654E + 03	1.9201E + 04	1.1568E + 04	3.1622E + 03	1.0166E + 04	4.8286E + 03	3.0942E + 03
F25	avg_time	1.2515E + 00	1.9304E + 00	2.0071E + 00	5.9321E + 00	1.7752E + 00	**8.42E-01**	2.0608E + 00	9.53E-01	1.6797E + 00	1.4639E + 00
F26	min	**2.9000E + 03**	3.3499E + 03	4.3899E + 03	5.9066E + 03	1.6156E + 04	1.3952E + 04	4.8277E + 03	1.1313E + 04	6.1339E + 03	3.6016E + 03
F26	std	3.0217E + 03	3.2176E + 03	3.0454E + 03	1.4864E + 03	3.9830E + 03	**8.9202E + 02**	1.8481E + 03	9.3070E + 02	2.2441E + 03	2.1564E + 03
F26	avg	**6.7755E + 03**	7.8846E + 03	1.0189E + 04	9.7039E + 03	1.8957E + 04	1.5786E + 04	1.0471E + 04	1.3339E + 04	1.2249E + 04	1.0620E + 04
F26	avg_time	1.7368E + 00	2.7177E + 00	2.7537E + 00	6.3470E + 00	2.1650E + 00	**1.2253E + 00**	2.9811E + 00	1.3367E + 00	2.4515E + 00	1.8547E + 00
F27	min	**3.4222E + 03**	3.5536E + 03	3.5612E + 03	3.5529E + 03	5.3020E + 03	5.9984E + 03	3.8250E + 03	4.3198E + 03	3.5790E + 03	3.4918E + 03
F27	std	2.2118E + 02	**1.5769E + 02**	3.3239E + 02	1.9426E + 02	1.4602E + 03	4.9048E + 02	4.4742E + 02	3.7589E + 02	6.1723E + 02	5.0833E + 02
F27	avg	**3.7814E + 03**	3.8741E + 03	4.0119E + 03	3.8071E + 03	8.1165E + 03	6.9614E + 03	4.3237E + 03	4.9123E + 03	4.3609E + 03	4.0109E + 03
F27	avg_time	2.1029E + 00	3.0105E + 00	3.0589E + 00	6.4518E + 00	2.2886E + 00	**1.3463E + 00**	3.3188E + 00	1.4785E + 00	2.6900E + 00	2.0051E + 00
F28	min	3.2632E + 03	3.3548E + 03	3.6217E + 03	3.3710E + 03	1.0782E + 04	8.7776E + 03	3.3715E + 03	6.3539E + 03	3.5459E + 03	**3.2597E + 03**
F28	std	3.9596E + 01	1.3815E + 02	2.2344E + 02	2.0397E + 02	3.0936E + 03	9.3418E + 02	5.0302E + 01	5.4345E + 02	2.2931E + 03	**2.5011E + 01**
F28	avg	**3.3160E + 03**	3.5580E + 03	3.9322E + 03	3.5424E + 03	1.4686E + 04	1.0412E + 04	3.4849E + 03	7.4758E + 03	5.0322E + 03	3.3295E + 03
F28	avg_time	1.5633E + 00	2.4339E + 00	2.4922E + 00	6.2219E + 00	2.0256E + 00	**1.1020E + 00**	2.6348E + 00	1.2248E + 00	2.1972E + 00	1.7234E + 00
F29	min	**3.8663E + 03**	5.1767E + 03	6.0234E + 03	4.4477E + 03	2.1969E + 04	9.6606E + 03	4.8215E + 03	6.7018E + 03	5.0363E + 03	4.1981E + 03
F29	std	4.5251E + 02	5.9862E + 02	9.1075E + 02	**4.4991E + 02**	8.7137E + 05	1.4635E + 04	7.4285E + 02	2.3959E + 03	2.9459E + 03	5.4957E + 02
F29	avg	**5.1855E + 03**	6.2605E + 03	7.2570E + 03	5.2400E + 03	4.3439E + 05	1.8771E + 04	5.9653E + 03	9.8534E + 03	7.4312E + 03	5.3578E + 03
F29	avg_time	1.2949E + 00	1.9646E + 00	2.0204E + 00	5.9404E + 00	1.7856E + 00	**8.48E-01**	2.1197E + 00	9.80E-01	1.7003E + 00	1.4704E + 00
F30	min	1.0499E + 06	2.6709E + 07	1.1229E + 08	**7.3488E + 05**	5.1240E + 09	6.1460E + 08	1.9890E + 07	2.6873E + 08	1.1460E + 07	9.5695E + 05
F30	std	2.1081E + 06	4.2226E + 07	7.2096E + 07	6.2396E + 05	3.4623E + 09	1.3461E + 09	1.4357E + 07	9.6772E + 08	1.3158E + 09	**6.0593E + 05**
F30	avg	3.3735E + 06	7.9200E + 07	2.2562E + 08	**1.4235E + 06**	8.2430E + 09	2.5208E + 09	3.8577E + 07	1.3979E + 09	4.3490E + 08	1.5042E + 06
F30	avg_time	3.0189E + 00	5.0095E + 00	4.9992E + 00	7.6778E + 00	3.2841E + 00	**2.3638E + 00**	5.7807E + 00	2.5129E + 00	4.7748E + 00	3.0538E + 00

Unimodal functions (F1, F3):EPO achieved the best results in all three metrics—avg, min, and std—on F1. On F3, however, it ranked second in both avg and min, and seventh in std. Moreover, its runtime was significantly higher than that of the fastest algorithm (HOA), with an overhead of approximately 115%–124%.

Multimodal functions (F4–F10):EPO obtained the best avg on F4, F7, F9, and F10, and achieved the best min on F7–F10. However, it failed to secure any top ranking in std across these seven functions (win rate: 0%). Its runtime was consistently 1.8–2.2 times that of HOA.

Hybrid functions (F11–F20):EPO attained the best avg only on F11 and the best min on F12, F19, and F20. On the remaining functions, it exhibited substantial gaps compared to leading algorithms—particularly SMO—with avg values exceeding the best results by over 100% on functions such as F13 and F14. Furthermore, EPO recorded no wins in std, and its runtime was notably higher than that of the most efficient competitors.

Composition functions (F21–F30):EPO demonstrated strong optimization capability on complex composite problems, achieving the best avg on F25–F29 and the best min on F25–F27 and F29. Nevertheless, it did not achieve a single top ranking in std across this category. Its runtime ranged from 1.4 to 2.3 times that of HOA.

Across all 116 valid test scenarios, EPO achieved an overall win rate of 0.90 against PO. Specifically, it significantly outperformed PO in avg (0.97), avg_time (1.00), and min (0.97). In summary, EPO demonstrates superior performance over PO in the majority of evaluation metrics and exhibits greater overall competitiveness.

**Fig. 13:**
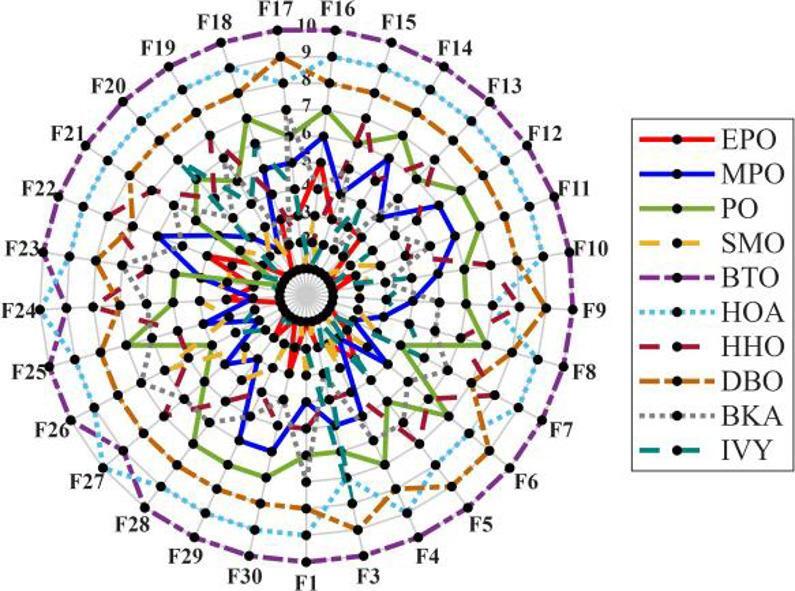
Radar chart.

**Fig. 14:**
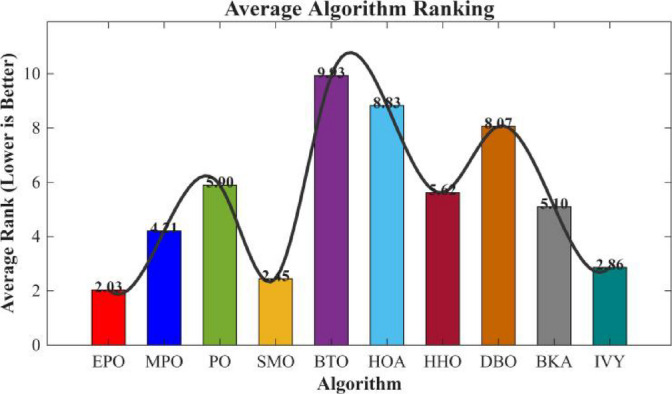
Average ranking.

**Fig. 15:**
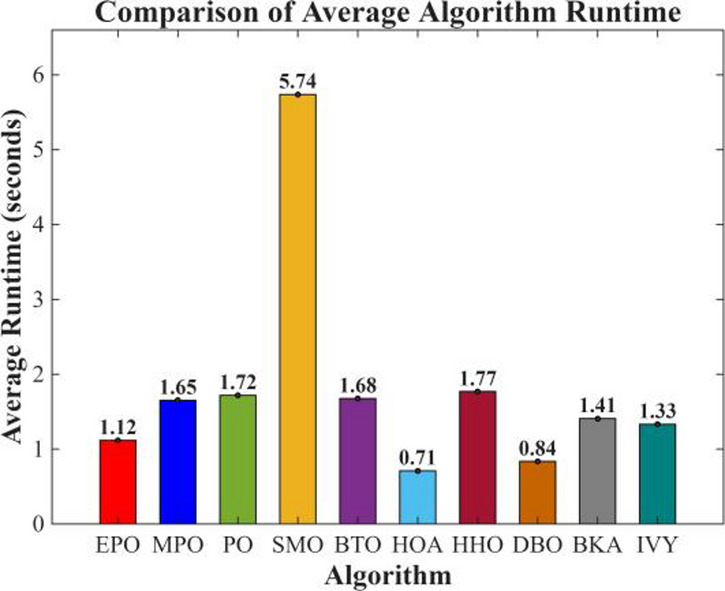
Comparison of average runtime.

**Fig. 16:**
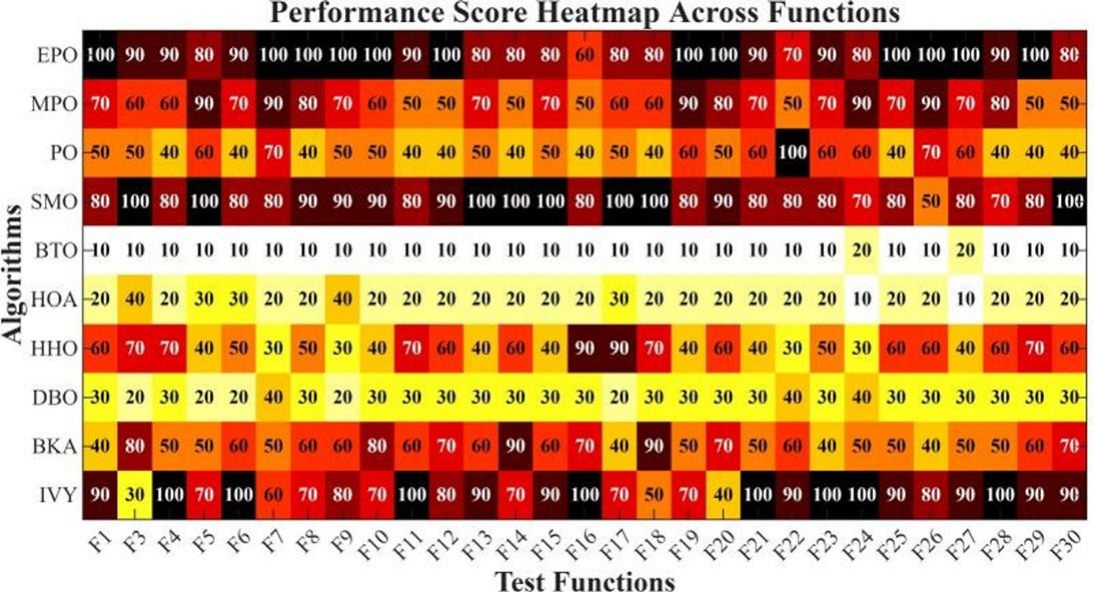
Performance score heatmap across functions.

Figures 13, 14, 15 and 16 show the radar chart, average ranking, comparison of average runtime, and performance score heatmap of the proposed EPO algorithm and comparison algorithms on the CEC2017 benchmark suite (D = 50).

As shown in Figs. 13, 14, 15 and 16, the proposed EPO algorithm achieves optimal performance on most functions., achieves the best overall performance with an average rank of 2.03, securing first place. It exhibits a statistically significant margin over subsequent algorithms, including IVY (average rank: 2.86) and SMO (2.45). The complete ranking order is as follows: EPO > SMO > IVY > MPO > BKA > PO > HHO > DBO > HOA > BTO.The complete ranking order of average runtime is as follows: HOA > DBO > EPO > IVY > BKA > MPO > BTO > PO > HHO > SMO.In summary, EPO achieves a balanced integration of performance and efficiency.

#### Convergence analysis

**Fig. 17:**
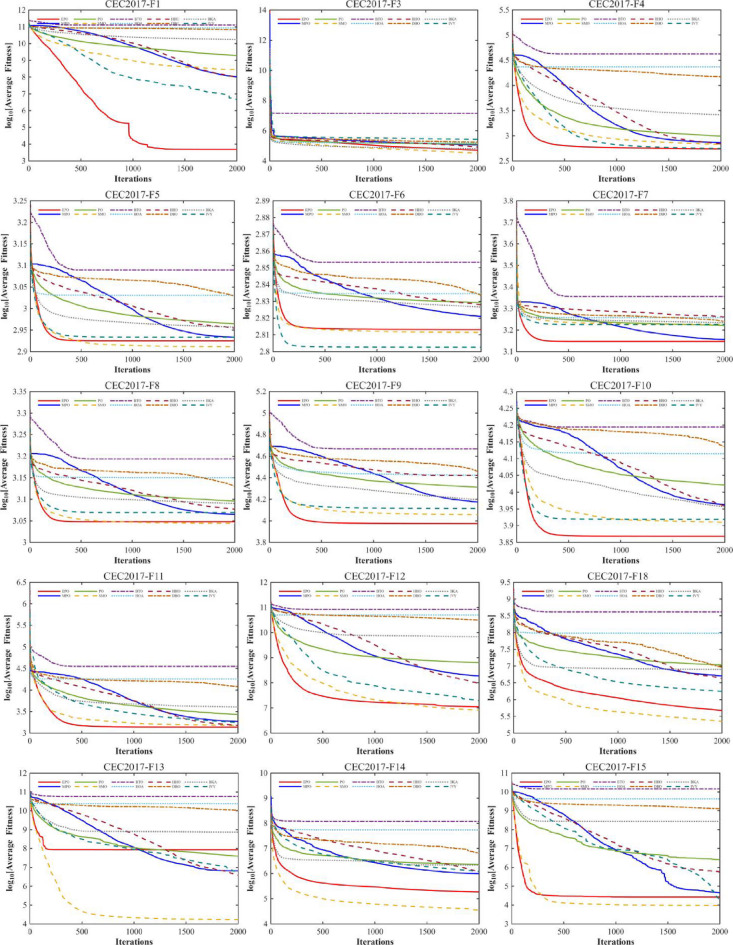

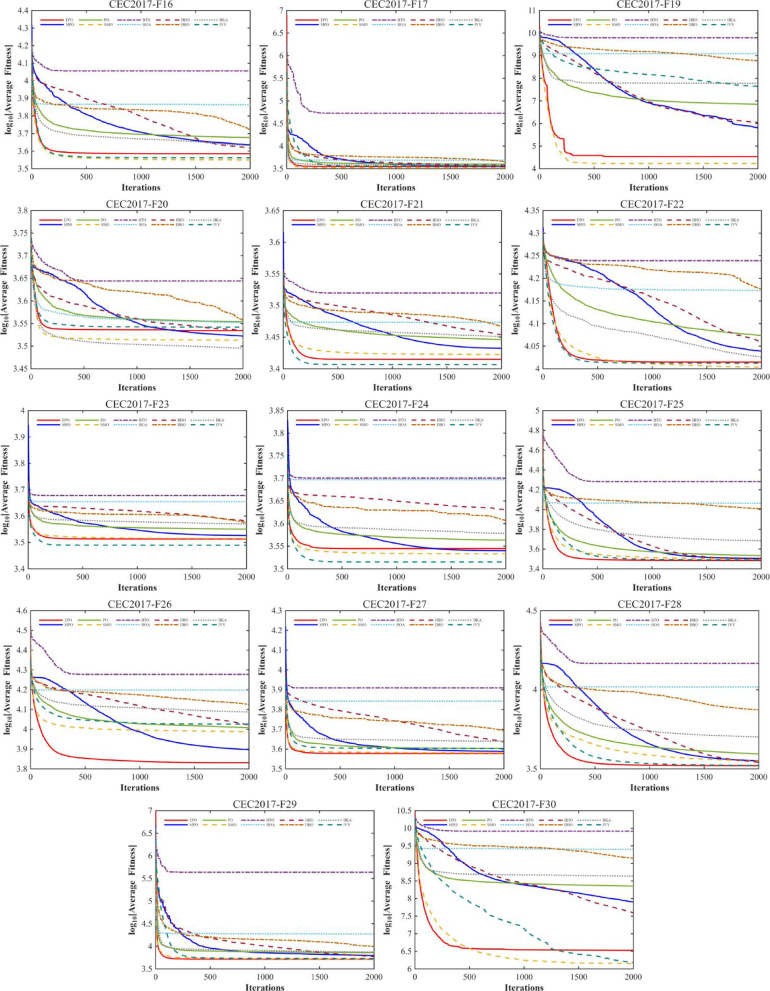
The convergence curves of the proposed technique and other algorithms for IEEE CEC2017 benchmark functions (Dim = 50).

A comprehensive comparative analysis was conducted using the 30-dimensional (D = 5000) functions of the CEC2017 benchmark suite. The performance of the proposed algorithm was benchmarked against nine state-of-the-art metaheuristics, namely MPO, PO, SMO, BTO, HOA, HHO, DBO, BKA, and IVY. All algorithms were executed for 30 independent trials with a population size (Np) of 30 and a maximum of 1,000 iterations (Tmax). The comparative performance is visually illustrated through convergence curves (Fig. 17).

Experimental results on the 50-dimensional CEC2017 benchmark suite (F1–F30, excluding F2),shown in Fig. 17, reveal that the proposed Enhanced Particle Optimizer (EPO)—an improved variant of the original PO algorithm—demonstrates strong convergence efficiency and stability across diverse problem landscapes.

1. **Unimodal functions (F1**,** F3)**, EPO achieves the best accuracy among all algorithms on F1, with rapid convergence and stable performance. On F3 (Shifted and Rotated Rosenbrock), EPO exhibits continuous improvement throughout the search process, though its final accuracy remains slightly inferior to SMO, reflecting a minor limitation in fine-tuning within narrow valleys.
2. **Simple multimodal functions (F4–F10)**, EPO attains the highest solution quality on F4, F7, F9, and F10, showcasing robust global exploration and effective escape from local optima. However, on F5, F6, and F8, while EPO converges quickly and stably, its precision is consistently outperformed by SMO; notably, on F6, IVY demonstrates markedly superior performance, suggesting that EPO’s diversity maintenance mechanism may be less effective in highly oscillatory landscapes.
3. **Hybrid functions (F11–F20)**, EPO secures the best result on F11, highlighting its ability to coordinate heterogeneous subcomponents. Nevertheless, on most other hybrid functions (F12–F20), EPO—despite fast and stable convergence—is consistently surpassed by SMO, and in several cases (e.g., F13, F14, F15, F16) also by MPO, PO, IVY, or HHO. This indicates that while EPO efficiently explores complex mixed-structure search spaces, its local exploitation depth is insufficient to match the most advanced competitors in high-dimensional, strongly coupled scenarios.
4. **Composition functions (F21–F30)**, EPO excels on F25–F29, achieving both the fastest convergence and the highest accuracy among all algorithms—a testament to its effectiveness in handling highly non-separable, rotated, and shifted composite problems. However, on F21–F24 and F30, EPO is outperformed primarily by IVY and SMO, with MPO occasionally showing competitive late-stage refinement (e.g., F24). These results suggest that EPO’s strength lies in mid-to-high complexity composition problems where structural regularity exists, but it faces challenges in the most irregular or scale-heterogeneous compositions.

Overall, EPO significantly outperforms the original PO and the alternative PO variant (MPO) across the majority of test cases, confirming the efficacy of the proposed enhancements. While SMO and IVY exhibit superior precision on many hybrid and composition functions, EPO maintains a compelling balance between convergence speed, stability, and solution quality, particularly in unimodal, certain multimodal, and mid-tier composition problems.

#### Analysis of box plot results

**Fig. 18:**
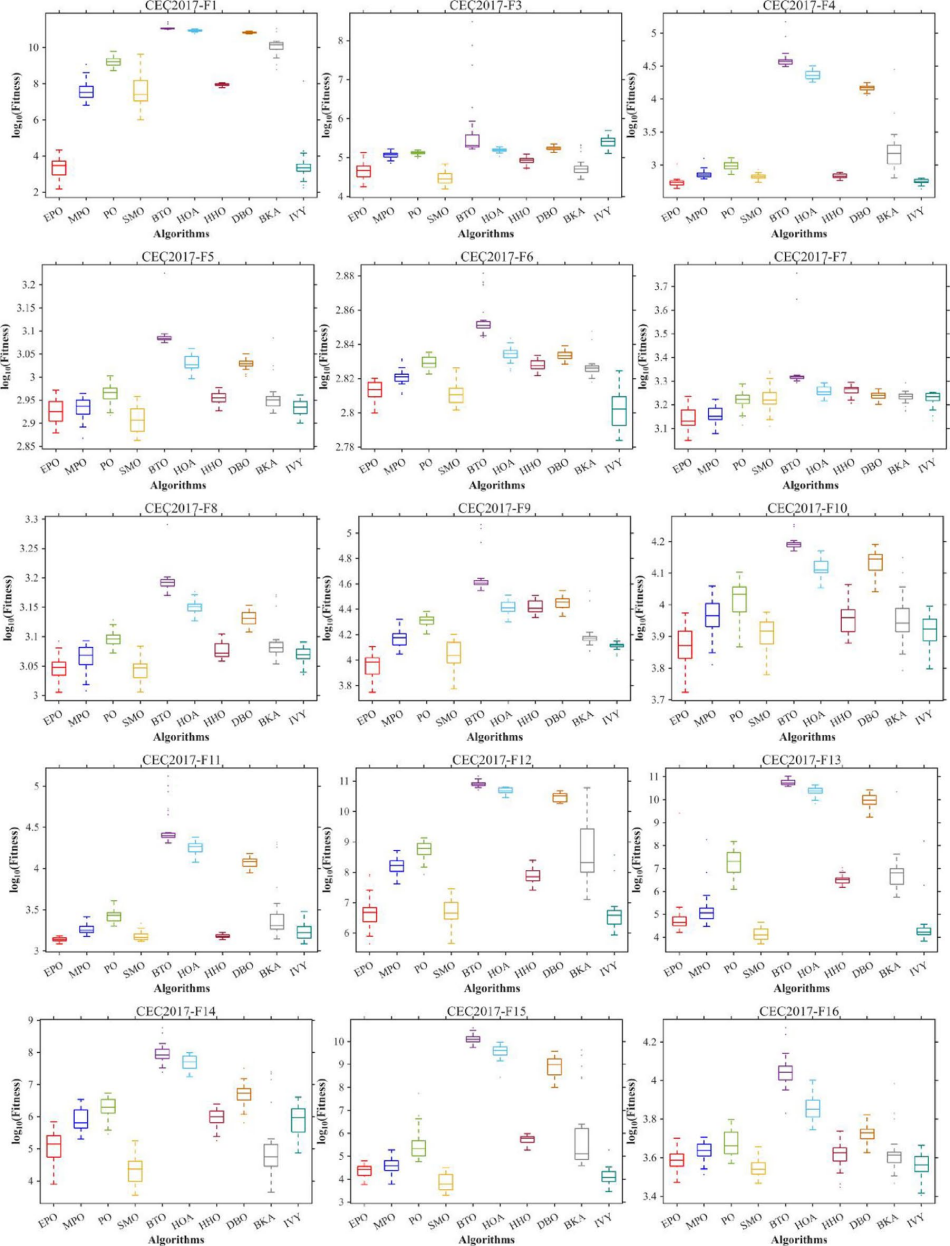

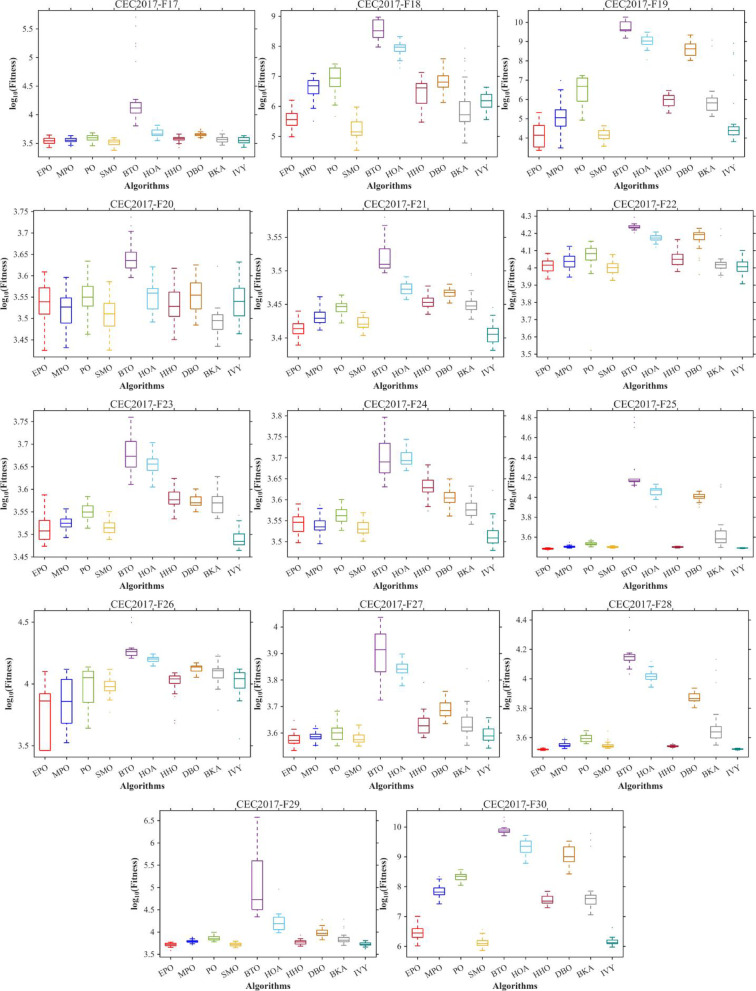
Boxplots for the proposed technique and other algorithms for IEEE CEC2017 benchmark functions (Dim = 50).

Figure 18 is the box graph obtained after 30 runs of the algorithm on CEC2027 with D = 50, The box plots graphically illustrate the performance distribution of EPO relative to the canonical PO algorithm and other benchmark metaheuristics.

As illustrated in Fig. 18, the EPO algorithm exhibits excellent overall optimization performance. It achieves the lowest median error among all compared algorithms on 10 benchmark functions (F4, F7, F9, F10, F11, F19, F25, F27, F28, and F29). Although EPO does not attain the globally smallest interquartile range (IQR) on any function, its IQR values remain consistently competitive across the test suite. Particularly noteworthy is that EPO produces no outliers on 21 functions, demonstrating exceptional run-to-run stability.

In direct comparison with the PO algorithm, EPO yields lower median errors on all 30 test functions, achieves smaller IQRs on 12 functions, and exhibits zero outliers on 24 functions. These results conclusively demonstrate that EPO comprehensively outperforms PO in terms of accuracy, stability, and robustness, underscoring its superior practical applicability in real-world optimization scenarios.

#### Analysis of Wilcoxon rank sum test results

**Fig. 19:**
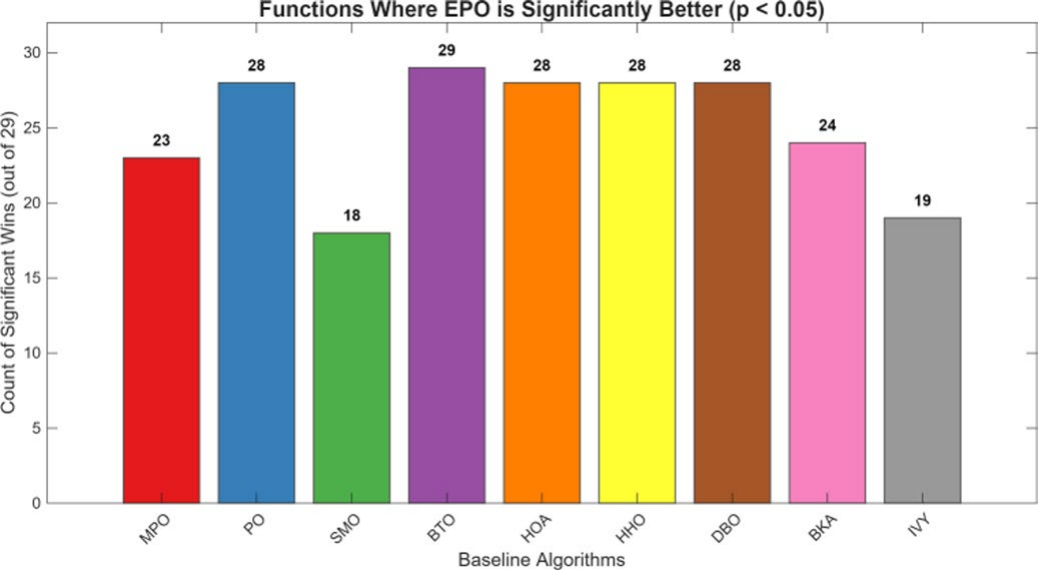
Boxplots for the proposed technique and other algorithms for IEEE CEC2017 benchmark functions (Dim = 50).

To rigorously evaluate the performance advantages of the proposed EPO algorithm, pairwise Wilcoxon rank-sum tests were conducted at a significance level of α = 0.05 between EPO and nine state-of-the-art optimization algorithms—MPO, PO, SMO, BTO, HOA, HHO, DBO, BKA, and IVY—across the 29 CEC2017 benchmark functions. The results of the Wilcoxon rank-sum tests are presented in Table 7; Fig. 19.

**Table 7:** Wilcoxon rank sum test results.

Function	MPO	PO	SMO	BTO	HOA	HHO	DBO	BKA	IVY
F1	3.0199E-11	3.0199E-11	3.0199E-11	3.0199E-11	3.0199E-11	3.0199E-11	3.0199E-11	3.0199E-11	**5.0114E-01**
F3	5.9673E-09	4.6159E-10	4.2259E-03	3.0199E-11	4.5043E-11	1.8608E-06	3.0199E-11	**1.7613E-01**	3.6897E-11
F4	5.0723E-10	2.6099E-10	5.9673E-09	3.0199E-11	3.0199E-11	7.3803E-10	3.0199E-11	6.6955E-11	**5.3685E-02**
F5	**1.4945E-01**	4.8011E-07	**5.5546E-02**	3.0199E-11	3.0199E-11	6.7362E-06	3.0199E-11	1.4067E-04	**1.8577E-01**
F6	2.7829E-07	3.0199E-11	**1.3345E-01**	3.0199E-11	3.0199E-11	3.0199E-11	3.0199E-11	6.6955E-11	1.8916E-04
F7	**2.5805E-01**	3.2555E-07	7.5991E-07	3.0199E-11	4.9752E-11	7.3891E-11	3.1589E-10	2.2273E-09	1.8500E-08
F8	2.2658E-03	2.8716E-10	**6.2040E-01**	3.0199E-11	3.0199E-11	1.8731E-07	3.0199E-11	2.8314E-08	2.7726E-05
F9	2.3715E-10	3.0199E-11	4.8560E-03	3.0199E-11	3.0199E-11	3.0199E-11	3.0199E-11	6.6955E-11	2.3715E-10
F10	1.1077E-06	5.0723E-10	4.8560E-03	3.0199E-11	3.0199E-11	1.0277E-06	3.0199E-11	6.2828E-06	1.3703E-03
F11	3.6897E-11	3.0199E-11	3.1830E-03	3.0199E-11	3.0199E-11	2.3768E-07	3.0199E-11	4.6159E-10	1.6351E-05
F12	8.9934E-11	3.0199E-11	**6.4142E-01**	3.0199E-11	3.0199E-11	9.7555E-10	3.0199E-11	1.6132E-10	**4.6427E-01**
F13	6.7650E-05	5.5727E-10	2.0152E-08	3.0199E-11	3.0199E-11	5.5727E-10	3.6897E-11	5.0723E-10	5.1857E-07
F14	1.2023E-08	8.9934E-11	1.1077E-06	3.0199E-11	3.0199E-11	1.1737E-09	3.3384E-11	**5.0120E-02**	6.0459E-07
F15	1.1711E-02	3.3384E-11	4.8011E-07	3.0199E-11	3.0199E-11	3.0199E-11	3.0199E-11	8.9934E-11	6.5486E-04
F16	1.4067E-04	8.1975E-07	4.4272E-03	3.0199E-11	3.0199E-11	2.4157E-02	1.2057E-10	**2.2257E-01**	**1.2967E-01**
F17	**4.0354E-01**	3.9881E-04	**5.7460E-02**	3.0199E-11	3.4742E-10	1.2212E-02	1.0702E-09	**1.8577E-01**	**4.2896E-01**
F18	5.5727E-10	9.9186E-11	3.1821E-04	3.0199E-11	3.0199E-11	3.8249E-09	4.0772E-11	3.6439E-02	8.4848E-09
F19	1.4298E-05	4.9752E-11	**8.8830E-01**	3.0199E-11	3.0199E-11	3.3384E-11	3.0199E-11	6.0658E-11	**9.0490E-02**
F20	**2.9047E-01**	**1.6687E-01**	**5.7460E-02**	6.0658E-11	**2.5805E-01**	**7.7312E-01**	**1.2967E-01**	4.4592E-04	**8.3026E-01**
F21	2.4913E-06	1.6132E-10	1.5638E-02	3.0199E-11	3.0199E-11	4.9752E-11	3.0199E-11	1.2057E-10	6.6689E-03
F22	3.3874E-02	2.4913E-06	**3.1830E-01**	3.0199E-11	3.0199E-11	1.0035E-03	1.2870E-09	**9.5873E-01**	**8.3026E-01**
F23	6.0971E-03	3.0103E-07	**3.0418E-01**	3.0199E-11	3.0199E-11	5.0723E-10	7.3803E-10	3.8249E-09	2.0058E-04
F24	**5.0114E-01**	2.4994E-03	4.5146E-02	3.0199E-11	3.0199E-11	4.9752E-11	2.1544E-10	1.1937E-06	1.0907E-05
F25	1.2057E-10	3.0199E-11	6.0658E-11	3.0199E-11	3.0199E-11	3.6897E-11	3.0199E-11	3.0199E-11	2.6784E-06
F26	**1.8090E-01**	2.8389E-04	3.5923E-05	3.0199E-11	3.0199E-11	2.6784E-06	6.6955E-11	9.2603E-09	2.4913E-06
F27	2.6077E-02	2.8913E-03	**5.7929E-01**	3.0199E-11	3.0199E-11	2.1947E-08	4.5043E-11	3.5201E-07	1.9883E-02
F28	1.4643E-10	3.0199E-11	8.1527E-11	3.0199E-11	3.0199E-11	4.5043E-11	3.0199E-11	3.0199E-11	1.0763E-02
F29	3.8249E-09	3.0199E-11	**8.3026E-01**	3.0199E-11	3.0199E-11	8.2919E-06	3.0199E-11	1.2023E-08	**2.2257E-01**
F30	3.0199E-11	3.0199E-11	8.1975E-07	3.0199E-11	3.0199E-11	3.0199E-11	3.0199E-11	3.0199E-11	2.1540E-06

The results presented in Table 7; Fig. 19 indicate that, out of a total of 261 pairwise Wilcoxon rank-sum tests, the EPO algorithm achieved significantly better performance in 225 cases (86.2%) at the significance level of *p* < 0.05. Specifically, EPO attained win rates exceeding 93% against PO, BTO, HOA, HHO, and DBO, and demonstrated statistically superior performance on all 29 functions when compared to BTO (100% win rate). In contrast, SMO and IVY exhibited relatively competitive performance, showing no significant difference from EPO on at least 25% of the test functions. In summary, EPO not only demonstrates highly significant performance advantages in the vast majority of test scenarios but also exhibits exceptional robustness across diverse optimization problems.

### Performance analysis on 100-dimensional functions

#### Statistics analysis

To evaluate performance, a comprehensive analysis was conducted using the 30-dimensional (D = 100) functions of the CEC2017 benchmark suite. The proposed algorithm was benchmarked against nine state-of-the-art metaheuristics, namely MPO, PO, SMO, BTO, HOA, HHO, DBO, BKA, and IVY. For all experiments, the population size (Np) was set to 30, the maximum number of iterations (Tmax) was 1,000, and each algorithm was executed for 30 independent runs. Performance was quantified using five statistical indicators: minimum (Min), mean (Avg), median (Median), worst (Worst), and standard deviation (Std). These results are presented in Table 8.

**Table 8:** The statistical results of benchmark functions using the proposed technique and other algorithms (Dim = 30).

Function	Item	EPO	MPO	PO	SMO	BTO	HOA	HHO	DBO	BKA	IVY
F1	min	1.6235E + 05	9.2299E + 08	1.1907E + 10	6.7368E + 09	2.5246E + 11	2.1053E + 11	7.7113E + 08	1.9870E + 11	4.9257E + 10	**1.3845E + 03**
F1	std	**1.9550E + 06**	3.2144E + 09	4.5096E + 09	9.6600E + 09	1.1296E + 11	1.1451E + 10	1.6932E + 08	9.1060E + 09	1.5245E + 10	4.3315E + 08
F1	avg	**1.5641E + 06**	4.1587E + 09	2.0684E + 10	1.9894E + 10	3.0468E + 11	2.3078E + 11	1.0062E + 09	2.1483E + 11	7.5418E + 10	1.9967E + 08
F1	avg_time	9.41E-01	1.4115E + 00	1.5821E + 00	5.8464E + 00	2.3759E + 00	**4.79E-01**	1.2628E + 00	6.10E-01	9.93E-01	1.1181E + 00
F3	min	**1.3588E + 05**	2.9631E + 05	2.8334E + 05	1.6330E + 05	3.2943E + 05	2.8482E + 05	2.3892E + 05	2.8576E + 05	1.5314E + 05	3.0328E + 05
F3	std	3.5997E + 04	4.3044E + 04	1.6566E + 04	3.6844E + 04	1.5681E + 10	**1.0110E + 04**	1.6162E + 04	5.6091E + 04	1.8735E + 04	1.8300E + 05
F3	avg	2.1657E + 05	3.4761E + 05	3.1890E + 05	2.3838E + 05	2.8685E + 09	3.1176E + 05	2.6927E + 05	3.6588E + 05	**1.8591E + 05**	5.9848E + 05
F3	avg_time	9.54E-01	1.3874E + 00	1.5461E + 00	5.7413E + 00	2.3756E + 00	**4.81E-01**	1.3017E + 00	6.45E-01	9.91E-01	1.1215E + 00
F4	min	**6.1778E + 02**	1.0438E + 03	2.0696E + 03	1.5812E + 03	8.7937E + 04	4.9981E + 04	1.0045E + 03	4.1513E + 04	3.9541E + 03	7.0264E + 02
F4	std	**6.3148E + 01**	6.4433E + 02	6.7062E + 02	1.0261E + 03	6.5555E + 04	1.1664E + 04	1.3620E + 02	6.4894E + 03	2.6145E + 04	8.6723E + 01
F4	avg	**7.4010E + 02**	1.6672E + 03	3.0781E + 03	2.8579E + 03	1.3012E + 05	6.7663E + 04	1.2577E + 03	5.2053E + 04	1.6512E + 04	8.5275E + 02
F4	avg_time	9.25E-01	1.4235E + 00	1.5876E + 00	5.8577E + 00	2.3885E + 00	**4.90E-01**	1.2716E + 00	6.30E-01	1.0072E + 00	1.1252E + 00
F5	min	1.2024E + 03	1.2933E + 03	1.4886E + 03	**1.1103E + 03**	2.0642E + 03	1.8128E + 03	1.4361E + 03	1.8067E + 03	1.3270E + 03	1.2661E + 03
F5	std	5.8715E + 01	5.7032E + 01	5.8916E + 01	1.2022E + 02	3.5953E + 02	4.6981E + 01	5.5791E + 01	5.3164E + 01	1.6935E + 02	**3.8566E + 01**
F5	avg	**1.3127E + 03**	1.4451E + 03	1.5683E + 03	1.3670E + 03	2.2681E + 03	1.9391E + 03	1.5556E + 03	1.8948E + 03	1.4579E + 03	1.3406E + 03
F5	avg_time	1.2499E + 00	1.9248E + 00	2.0922E + 00	6.1368E + 00	2.6177E + 00	**7.43E-01**	1.9553E + 00	8.75E-01	1.5236E + 00	1.3867E + 00
F6	min	6.4716E + 02	6.5879E + 02	6.7917E + 02	6.4864E + 02	7.1119E + 02	6.8734E + 02	6.7922E + 02	6.8662E + 02	6.6716E + 02	**6.4027E + 02**
F6	std	4.9258E + 00	4.9647E + 00	4.4961E + 00	5.9638E + 00	1.2835E + 01	4.4685E + 00	3.6097E + 00	**3.2337E + 00**	1.1551E + 01	6.7391E + 00
F6	avg	6.5939E + 02	6.7240E + 02	6.8629E + 02	6.6186E + 02	7.1827E + 02	6.9706E + 02	6.8574E + 02	6.9428E + 02	6.7573E + 02	**6.5636E + 02**
F6	avg_time	2.0126E + 00	3.2040E + 00	3.3364E + 00	6.8172E + 00	3.2578E + 00	**1.3574E + 00**	3.4723E + 00	1.4982E + 00	2.7303E + 00	2.0294E + 00
F7	min	**2.3937E + 03**	2.5179E + 03	3.0694E + 03	2.8916E + 03	3.8885E + 03	3.3564E + 03	3.2925E + 03	3.4323E + 03	3.0631E + 03	2.5655E + 03
F7	std	2.2992E + 02	2.7102E + 02	1.3726E + 02	4.2294E + 02	1.9445E + 03	1.5251E + 02	1.6764E + 02	**7.3738E + 01**	1.6584E + 02	2.0541E + 02
F7	avg	**2.8561E + 03**	3.1219E + 03	3.4056E + 03	3.7874E + 03	4.3886E + 03	3.6904E + 03	3.6876E + 03	3.5756E + 03	3.3388E + 03	3.1152E + 03
F7	avg_time	1.2764E + 00	1.9492E + 00	2.0907E + 00	6.2092E + 00	2.6453E + 00	**7.53E-01**	1.9583E + 00	8.77E-01	1.5561E + 00	1.4224E + 00
F8	min	1.5483E + 03	1.5220E + 03	1.9712E + 03	**1.4862E + 03**	2.5894E + 03	2.2247E + 03	1.8490E + 03	2.1095E + 03	1.7032E + 03	1.5512E + 03
F8	std	9.2525E + 01	1.3050E + 02	7.2332E + 01	1.2394E + 02	2.8271E + 02	**6.4494E + 01**	7.5068E + 01	6.8160E + 01	2.4242E + 02	8.6787E + 01
F8	avg	**1.7130E + 03**	1.8741E + 03	2.0812E + 03	1.7697E + 03	2.7223E + 03	2.3915E + 03	1.9880E + 03	2.3061E + 03	1.9765E + 03	1.7955E + 03
F8	avg_time	1.2736E + 00	1.9741E + 00	2.1186E + 00	6.1619E + 00	2.6572E + 00	**7.56E-01**	1.9777E + 00	8.90E-01	1.5419E + 00	1.4090E + 00
F9	min	**1.9127E + 04**	2.3918E + 04	4.0265E + 04	2.0860E + 04	7.4564E + 04	5.0247E + 04	4.6176E + 04	5.9272E + 04	2.6025E + 04	2.0755E + 04
F9	std	1.6764E + 03	4.4981E + 03	6.0590E + 03	3.7941E + 03	5.5207E + 04	5.8164E + 03	6.3771E + 03	5.7987E + 03	1.3483E + 04	**7.3001E + 02**
F9	avg	2.3258E + 04	3.5341E + 04	5.0411E + 04	2.8207E + 04	1.0969E + 05	6.2111E + 04	5.7658E + 04	7.2234E + 04	3.3739E + 04	**2.3119E + 04**
F9	avg_time	1.2736E + 00	1.9747E + 00	2.1257E + 00	6.2270E + 00	2.6383E + 00	**7.64E-01**	2.0383E + 00	9.18E-01	1.5399E + 00	1.4102E + 00
F10	min	**1.3162E + 04**	1.6727E + 04	2.1114E + 04	1.5652E + 04	3.2129E + 04	2.6425E + 04	1.8760E + 04	2.7292E + 04	1.4502E + 04	1.3936E + 04
F10	std	1.6180E + 03	1.8409E + 03	1.5019E + 03	1.2140E + 03	**1.0401E + 03**	1.3082E + 03	1.2435E + 03	1.2690E + 03	3.2115E + 03	1.3074E + 03
F10	avg	**1.5839E + 04**	2.0746E + 04	2.3956E + 04	1.8159E + 04	3.3248E + 04	2.8830E + 04	2.1332E + 04	3.0761E + 04	1.8604E + 04	1.6526E + 04
F10	avg_time	1.4819E + 00	2.3051E + 00	2.4704E + 00	6.3512E + 00	2.7801E + 00	**9.00E-01**	2.3896E + 00	1.1064E + 00	1.8642E + 00	1.5734E + 00
F11	min	**2.6638E + 03**	2.8886E + 04	6.0259E + 04	4.9284E + 03	1.9763E + 05	1.0790E + 05	1.0188E + 04	1.0537E + 05	1.4923E + 04	2.0575E + 04
F11	std	2.1282E + 04	1.4089E + 04	1.5373E + 04	**6.7659E + 03**	2.3761E + 09	3.1983E + 04	8.8075E + 03	2.5991E + 04	3.5375E + 04	2.0375E + 04
F11	avg	1.4630E + 04	5.0566E + 04	9.2593E + 04	**1.3109E + 04**	6.1035E + 08	1.7333E + 05	2.7884E + 04	1.5630E + 05	3.5734E + 04	5.1869E + 04
F11	avg_time	1.0876E + 00	1.6406E + 00	1.8017E + 00	5.9369E + 00	2.5064E + 00	**6.00E-01**	1.5749E + 00	7.63E-01	1.2270E + 00	1.2518E + 00
F12	min	6.4187E + 06	2.7771E + 08	2.3747E + 09	1.6156E + 08	1.5884E + 11	1.0923E + 11	3.3179E + 08	9.2949E + 10	3.2264E + 09	**5.6740E + 06**
F12	std	6.1364E + 07	7.1655E + 08	9.2673E + 08	7.4642E + 08	4.8120E + 10	1.8509E + 10	2.1809E + 08	1.0663E + 10	5.5881E + 10	**1.5144E + 07**
F12	avg	4.1336E + 07	1.2329E + 09	3.6765E + 09	9.3440E + 08	2.1970E + 11	1.4522E + 11	6.6441E + 08	1.1690E + 11	3.4974E + 10	**2.4859E + 07**
F12	avg_time	1.2582E + 00	1.9507E + 00	2.0901E + 00	6.1723E + 00	2.6509E + 00	**7.42E-01**	1.9100E + 00	8.95E-01	1.5111E + 00	1.3895E + 00
F13	min	**1.1665E + 04**	6.6052E + 04	1.1695E + 07	1.5607E + 04	4.1179E + 10	1.8674E + 10	6.4675E + 06	2.1546E + 10	1.7668E + 06	1.2329E + 04
F13	std	**4.0493E + 04**	2.0383E + 07	1.3623E + 08	1.3119E + 06	1.1726E + 10	5.4999E + 09	1.9812E + 06	3.0526E + 09	1.2373E + 10	4.1661E + 08
F13	avg	**5.4058E + 04**	6.0749E + 06	1.1604E + 08	2.8145E + 05	5.2157E + 10	2.9945E + 10	1.0191E + 07	2.8178E + 10	5.7997E + 09	9.4052E + 07
F13	avg_time	1.0667E + 00	1.6421E + 00	1.8387E + 00	6.0473E + 00	2.4999E + 00	**6.00E-01**	1.5924E + 00	7.47E-01	1.2255E + 00	1.2462E + 00
F14	min	**1.2408E + 05**	8.2285E + 05	4.5249E + 06	2.4928E + 05	4.6646E + 07	1.3572E + 07	2.3068E + 06	9.6415E + 06	3.8353E + 05	4.6048E + 05
F14	std	**2.2859E + 05**	3.1979E + 06	3.3896E + 06	4.7620E + 05	2.6398E + 08	1.2866E + 07	8.5834E + 05	6.1539E + 06	1.8558E + 07	7.2143E + 05
F14	avg	**4.2055E + 05**	6.0641E + 06	1.0647E + 07	8.3572E + 05	2.6164E + 08	3.1268E + 07	3.5733E + 06	1.8875E + 07	6.7566E + 06	1.7516E + 06
F14	avg_time	1.4382E + 00	2.2585E + 00	2.4018E + 00	6.2991E + 00	2.7954E + 00	**9.88E-01**	2.4922E + 00	1.1396E + 00	1.8560E + 00	1.5781E + 00
F15	min	9.2826E + 03	8.8755E + 03	1.8461E + 06	**3.7104E + 03**	1.9923E + 10	1.0782E + 10	1.2947E + 06	3.8893E + 09	4.8460E + 05	6.2677E + 03
F15	std	3.5910E + 04	8.0990E + 06	2.7207E + 07	**4.6203E + 03**	7.9357E + 09	3.5811E + 09	1.0631E + 06	2.5833E + 09	3.7506E + 09	3.4767E + 08
F15	avg	4.8856E + 04	1.6848E + 06	2.3825E + 07	**1.0588E + 04**	3.0631E + 10	1.5829E + 10	2.8071E + 06	9.5211E + 09	9.9763E + 08	1.1635E + 08
F15	avg_time	1.0738E + 00	1.6981E + 00	1.8448E + 00	5.8981E + 00	2.5462E + 00	**5.84E-01**	1.5579E + 00	7.37E-01	1.1966E + 00	1.2286E + 00
F16	min	**4.8617E + 03**	6.9056E + 03	8.4769E + 03	4.8773E + 03	2.2571E + 04	1.4035E + 04	6.6252E + 03	1.2305E + 04	5.0353E + 03	4.8972E + 03
F16	std	**5.4429E + 02**	1.1207E + 03	1.4106E + 03	6.7613E + 02	5.1067E + 03	1.7653E + 03	6.7427E + 02	1.2383E + 03	3.5018E + 03	6.4356E + 02
F16	avg	**6.0637E + 03**	8.8799E + 03	1.0856E + 04	6.1838E + 03	2.7852E + 04	1.7342E + 04	7.7400E + 03	1.4926E + 04	9.2287E + 03	6.0651E + 03
F16	avg_time	1.2073E + 00	1.8664E + 00	2.0252E + 00	6.0049E + 00	2.6831E + 00	**6.92E-01**	1.8218E + 00	8.59E-01	1.4263E + 00	1.3593E + 00
F17	min	5.1132E + 03	4.7435E + 03	5.7689E + 03	4.9062E + 03	1.2130E + 06	1.8789E + 05	4.9205E + 03	1.0243E + 04	**4.5185E + 03**	4.5988E + 03
F17	std	7.4939E + 02	8.5999E + 02	1.5471E + 03	7.0970E + 02	4.5817E + 07	1.9138E + 06	7.1114E + 02	3.0128E + 04	4.1481E + 05	**5.1464E + 02**
F17	avg	6.3029E + 03	6.5407E + 03	7.7793E + 03	6.0861E + 03	3.4473E + 07	2.5155E + 06	6.3499E + 03	3.0413E + 04	1.1544E + 05	**5.6570E + 03**
F17	avg_time	1.8414E + 00	2.7918E + 00	2.9615E + 00	6.4889E + 00	3.1295E + 00	**1.1571E + 00**	2.9738E + 00	1.3291E + 00	2.3841E + 00	1.8430E + 00
F18	min	4.4702E + 05	1.6973E + 06	1.7570E + 06	**3.3622E + 05**	6.7267E + 07	1.5720E + 07	1.3789E + 06	6.9653E + 06	3.6993E + 05	4.6458E + 05
F18	std	**5.1419E + 05**	2.3245E + 06	4.8998E + 06	1.6105E + 06	2.5516E + 08	2.4910E + 07	1.8156E + 06	1.2139E + 07	3.2625E + 07	8.6344E + 05
F18	avg	**1.1355E + 06**	5.9635E + 06	1.0968E + 07	2.0286E + 06	2.7148E + 08	6.4351E + 07	4.7015E + 06	2.5373E + 07	1.2367E + 07	1.5579E + 06
F18	avg_time	1.2183E + 00	1.8609E + 00	2.0299E + 00	5.9619E + 00	2.7072E + 00	**7.03E-01**	1.8391E + 00	8.65E-01	1.4396E + 00	1.3702E + 00
F19	min	2.6941E + 03	8.4710E + 04	5.4438E + 06	2.5834E + 03	1.6606E + 10	8.6680E + 09	3.8250E + 06	2.7698E + 09	4.2624E + 06	**2.4810E + 03**
F19	std	2.6159E + 05	8.4127E + 06	2.7369E + 07	**1.9779E + 04**	8.0773E + 09	3.3414E + 09	4.2063E + 06	2.8136E + 09	4.9505E + 09	3.5616E + 08
F19	avg	1.1004E + 05	7.5816E + 06	4.2095E + 07	**1.5249E + 04**	2.9866E + 10	1.5405E + 10	1.0307E + 07	7.8161E + 09	1.6930E + 09	7.6061E + 07
F19	avg_time	5.6858E + 00	9.2778E + 00	9.0144E + 00	9.8202E + 00	6.0023E + 00	**4.2238E + 00**	1.0284E + 01	4.3523E + 00	8.4985E + 00	4.9625E + 00
F20	min	**3.8643E + 03**	5.1634E + 03	5.3999E + 03	4.6691E + 03	7.6093E + 03	5.5345E + 03	5.0820E + 03	5.4448E + 03	4.9406E + 03	4.6239E + 03
F20	std	6.0755E + 02	5.3003E + 02	3.9374E + 02	5.9001E + 02	8.8094E + 02	5.3110E + 02	4.3008E + 02	6.1485E + 02	**3.1175E + 02**	5.5275E + 02
F20	avg	5.5020E + 03	5.8549E + 03	6.0627E + 03	5.6254E + 03	8.4314E + 03	6.6317E + 03	6.0624E + 03	7.0152E + 03	**5.4145E + 03**	5.6235E + 03
F20	avg_time	1.8967E + 00	2.8788E + 00	3.0451E + 00	6.5417E + 00	3.1151E + 00	**1.2208E + 00**	3.1310E + 00	1.3982E + 00	2.4871E + 00	1.8609E + 00
F21	min	2.9992E + 03	3.1483E + 03	3.5767E + 03	3.2137E + 03	4.5061E + 03	3.9655E + 03	3.6418E + 03	3.9398E + 03	3.5869E + 03	**2.7603E + 03**
F21	std	1.5664E + 02	1.6289E + 02	1.6967E + 02	1.4810E + 02	3.3744E + 02	1.9940E + 02	2.2015E + 02	**1.2893E + 02**	3.7253E + 02	1.9508E + 02
F21	avg	3.2668E + 03	3.5749E + 03	3.9400E + 03	3.4598E + 03	4.9529E + 03	4.4525E + 03	4.1381E + 03	4.2082E + 03	4.1709E + 03	**3.0606E + 03**
F21	avg_time	2.6528E + 00	4.2377E + 00	4.3520E + 00	7.4224E + 00	3.7635E + 00	**1.8641E + 00**	4.6389E + 00	2.0316E + 00	3.7530E + 00	2.5464E + 00
F22	min	**1.5002E + 04**	1.8831E + 04	2.1602E + 04	1.7830E + 04	3.3128E + 04	2.9265E + 04	2.2736E + 04	2.6495E + 04	1.7879E + 04	1.5060E + 04
F22	std	1.6895E + 03	1.8831E + 03	2.1164E + 03	1.2853E + 03	1.1411E + 03	**1.0060E + 03**	1.2315E + 03	1.9184E + 03	3.6192E + 03	1.9559E + 03
F22	avg	**1.9259E + 04**	2.2933E + 04	2.6317E + 04	2.0331E + 04	3.5303E + 04	3.1578E + 04	2.5276E + 04	3.2570E + 04	2.2556E + 04	1.9765E + 04
F22	avg_time	3.0419E + 00	4.7794E + 00	4.9189E + 00	7.6606E + 00	3.9233E + 00	**2.1122E + 00**	5.3836E + 00	2.3296E + 00	4.3118E + 00	2.8125E + 00
F23	min	3.8599E + 03	3.8701E + 03	4.2711E + 03	3.7384E + 03	6.0126E + 03	6.3800E + 03	4.7844E + 03	4.8942E + 03	4.3357E + 03	**3.2907E + 03**
F23	std	2.1679E + 02	2.3487E + 02	2.3369E + 02	2.4750E + 02	1.0891E + 03	4.9134E + 02	3.2104E + 02	2.7348E + 02	2.6583E + 02	**1.6237E + 02**
F23	avg	4.4854E + 03	4.4073E + 03	4.8320E + 03	4.2425E + 03	7.3984E + 03	7.3680E + 03	5.3068E + 03	5.3869E + 03	5.0071E + 03	**3.5976E + 03**
F23	avg_time	3.3752E + 00	5.4726E + 00	5.5651E + 00	8.1049E + 00	4.2204E + 00	**2.4326E + 00**	6.0280E + 00	2.6232E + 00	4.9231E + 00	3.1366E + 00
F24	min	4.5401E + 03	4.9068E + 03	5.2888E + 03	4.8181E + 03	9.3038E + 03	1.0229E + 04	5.9245E + 03	6.6174E + 03	5.3395E + 03	**4.0378E + 03**
F24	std	**3.7097E + 02**	4.3962E + 02	4.0707E + 02	4.1109E + 02	1.6649E + 03	7.9738E + 02	4.8517E + 02	9.1603E + 02	5.8955E + 02	5.1618E + 02
F24	avg	5.2779E + 03	5.5467E + 03	6.0710E + 03	5.5098E + 03	1.1412E + 04	1.1961E + 04	6.9430E + 03	8.1783E + 03	6.4891E + 03	**4.6621E + 03**
F24	avg_time	3.7325E + 00	5.9819E + 00	6.1031E + 00	8.4515E + 00	4.6288E + 00	**2.7358E + 00**	6.7573E + 00	2.9028E + 00	5.5209E + 00	3.4573E + 00
F25	min	**3.2819E + 03**	3.8669E + 03	4.6230E + 03	4.1133E + 03	2.5123E + 04	2.0829E + 04	3.7245E + 03	1.6144E + 04	5.7731E + 03	3.3252E + 03
F25	std	**4.8160E + 01**	2.6141E + 02	3.6698E + 02	7.4845E + 02	2.9437E + 04	1.9937E + 03	6.1555E + 01	9.9676E + 02	5.3903E + 03	1.3076E + 02
F25	avg	**3.3816E + 03**	4.1830E + 03	5.2128E + 03	5.0195E + 03	3.6774E + 04	2.4329E + 04	3.8358E + 03	1.8496E + 04	9.3497E + 03	3.5390E + 03
F25	avg_time	3.2284E + 00	5.1942E + 00	5.3161E + 00	8.0975E + 00	4.2825E + 00	**2.3412E + 00**	5.6851E + 00	2.4795E + 00	4.7256E + 00	3.0559E + 00
F26	min	**3.2833E + 03**	6.7028E + 03	1.6803E + 04	2.0804E + 04	4.9594E + 04	4.2822E + 04	1.4063E + 04	2.8709E + 04	2.3622E + 04	8.6185E + 03
F26	std	5.8938E + 03	5.2524E + 03	4.5869E + 03	3.1983E + 03	1.6010E + 04	3.2074E + 03	**2.6597E + 03**	4.1164E + 03	6.9329E + 03	4.2618E + 03
F26	avg	**1.7868E + 04**	2.5789E + 04	2.9578E + 04	2.6703E + 04	6.4896E + 04	4.8449E + 04	2.6308E + 04	3.6287E + 04	3.3110E + 04	2.5225E + 04
F26	avg_time	4.3969E + 00	6.7826E + 00	6.8478E + 00	8.9256E + 00	4.8678E + 00	**3.0737E + 00**	7.5854E + 00	3.6053E + 00	6.6572E + 00	3.9257E + 00
F27	min	**3.5985E + 03**	3.8430E + 03	3.8635E + 03	3.6761E + 03	1.2017E + 04	1.1016E + 04	3.7783E + 03	6.4906E + 03	3.9538E + 03	3.6077E + 03
F27	std	**2.1474E + 02**	4.6560E + 02	4.7472E + 02	3.2309E + 02	1.6689E + 03	1.2018E + 03	3.6716E + 02	9.5463E + 02	1.7159E + 03	4.5330E + 02
F27	avg	**3.8724E + 03**	4.3504E + 03	4.5776E + 03	4.2948E + 03	1.4826E + 04	1.3500E + 04	4.5852E + 03	8.0367E + 03	5.8273E + 03	4.2939E + 03
F27	avg_time	4.8272E + 00	7.6812E + 00	7.7743E + 00	9.4323E + 00	5.4667E + 00	**3.5422E + 00**	8.6756E + 00	3.7465E + 00	7.1665E + 00	4.3173E + 00
F28	min	3.4422E + 03	3.8397E + 03	5.0125E + 03	4.5575E + 03	2.9162E + 04	2.5602E + 04	3.8241E + 03	1.8451E + 04	6.3187E + 03	**3.4274E + 03**
F28	std	**3.8860E + 01**	6.8746E + 02	8.2875E + 02	1.3124E + 03	1.1820E + 04	2.3454E + 03	1.6679E + 02	1.5873E + 03	1.6616E + 03	4.4298E + 01
F28	avg	**3.5065E + 03**	4.5747E + 03	6.5890E + 03	6.4196E + 03	4.2925E + 04	3.1062E + 04	4.0430E + 03	2.1261E + 04	9.2507E + 03	3.5182E + 03
F28	avg_time	4.0755E + 00	6.5816E + 00	6.8637E + 00	8.9109E + 00	4.9032E + 00	**3.0196E + 00**	7.2996E + 00	3.1719E + 00	6.1052E + 00	3.7510E + 00
F29	min	**6.4794E + 03**	8.2305E + 03	1.1409E + 04	7.4591E + 03	1.8563E + 05	3.2054E + 04	8.5523E + 03	1.8459E + 04	9.7539E + 03	6.9250E + 03
F29	std	7.1676E + 02	1.3179E + 03	1.6337E + 03	7.8400E + 02	8.1075E + 06	8.7900E + 04	8.4855E + 02	1.3345E + 04	3.0981E + 04	**6.0834E + 02**
F29	avg	**7.8169E + 03**	1.1525E + 04	1.4272E + 04	9.2699E + 03	3.1292E + 06	1.3975E + 05	1.0237E + 04	3.8285E + 04	1.7488E + 04	8.1148E + 03
F29	avg_time	2.9322E + 00	4.6072E + 00	4.7153E + 00	7.6959E + 00	3.8966E + 00	**2.0382E + 00**	5.0788E + 00	2.2169E + 00	4.1537E + 00	2.7703E + 00
F30	min	9.9344E + 04	4.2307E + 07	1.4221E + 08	1.3043E + 05	3.6235E + 10	1.4783E + 10	4.2676E + 07	1.3251E + 10	3.4532E + 07	**5.3318E + 04**
F30	std	2.8875E + 06	2.8640E + 08	3.0991E + 08	5.0037E + 06	8.7701E + 09	5.4358E + 09	2.7269E + 07	3.5823E + 09	7.8667E + 09	**2.7462E + 05**
F30	avg	2.9145E + 06	2.9112E + 08	7.1218E + 08	1.8211E + 06	4.6164E + 10	2.7867E + 10	8.8976E + 07	2.2434E + 10	2.5576E + 09	**2.7530E + 05**
F30	avg_time	6.6503E + 00	1.0923E + 01	1.0960E + 01	1.1085E + 01	6.9524E + 00	**5.1336E + 00**	1.2562E + 01	5.3029E + 00	1.0274E + 01	5.8960E + 00

To rigorously assess the performance of the proposed EPO algorithm, a detailed comparative analysis was conducted across four function categories of the CEC2017 benchmark suite (D = 30), evaluating metrics including average value (avg), minimum value (min), standard deviation (std), and average runtime (avg_time). The results are summarized as follows.

Unimodal functions (F1, F3):EPO achieved a win rate of 0.17 (1/6) in each of the avg, min, and std metrics. Specifically, EPO obtained the best avg on F1 and ranked second on F3 (trailing BKA by 16.5%). For min, EPO achieved the best result on F3 and ranked second on F1 (behind IVY by a large margin of 11,626.2%). In terms of std, EPO performed best on F1 and ranked fifth on F3 (trailing HOA by 256.1%). Regarding avg_time, EPO ranked third on both F1 and F3, with runtimes 96.7% and 98.5% slower than the fastest algorithm (HOA), respectively.

Multimodal functions (F4–F10):EPO achieved win rates of 0.24 (5/21) for avg, 0.19 (4/21) for min, and 0.05 (1/21) for std. It obtained the best avg on F4, F5, F7, F8, and F10, and ranked second on F6 and F9 (with margins of 0.5% and 0.6%, respectively). For min, EPO was best on F4, F7, F9, and F10, and placed second or third on F5, F6, and F8, with gaps within 8.3%. However, except for F4, EPO generally ranked lower in std, particularly on F10 where it placed eighth (55.6% worse than the best). In avg_time, EPO consistently ranked third across all seven multimodal functions, with runtimes approximately 1.6–2.0 times that of HOA.

Hybrid functions (F11–F20):Win rates were 0.13 (4/30) for avg, 0.17 (5/30) for min, and 0.13 (4/30) for std. EPO achieved the best avg and std on F13, F14, F16, and F18, and obtained the best min on F11, F13, F14, F16, and F20. Nevertheless, notable gaps were observed on F12, F15, and F19; for instance, EPO’s avg on F15 was 361.4% higher than the best result, and its std on F19 was 1,222.6% worse. In terms of avg_time, EPO consistently ranked third or fourth across all ten hybrid functions, with runtimes 45.6%–84.0% slower than HOA.

Composition functions (F21–F30):EPO achieved win rates of 0.20 (6/30) for avg, 0.17 (5/30) for min, and 0.13 (4/30) for std. It obtained the best avg on F22, F25–F29 and the best min on F22, F25–F27, and F29. For std, EPO showed the highest stability on F24, F25, F27, and F28. However, on F30, EPO was significantly outperformed by IVY, with avg and std gaps of 958.6% and 951.5%, respectively. In avg_time, EPO ranked fourth on all composition functions, with runtimes 30%–44% slower than HOA.

Across all 116 valid test scenarios, EPO achieved an overall win rate of 0.93 against PO. Specifically, EPO significantly outperformed PO in avg (1.00), avg_time (1.00), min (1.00), and std (0.72). These results confirm that EPO is substantially more competitive than PO across the majority of evaluation metrics.

**Fig. 20:**
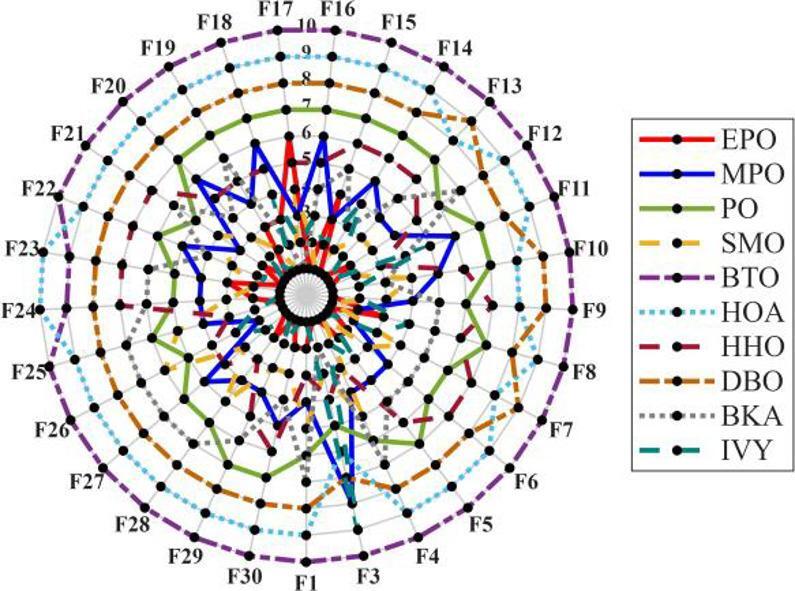
Radar chart.

**Fig. 21:**
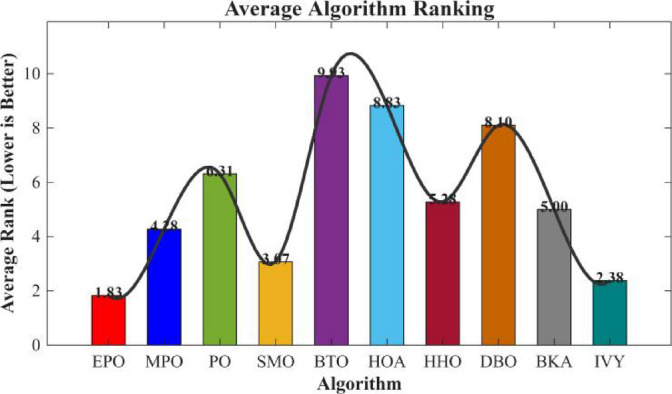
Average ranking.

**Fig. 22:**
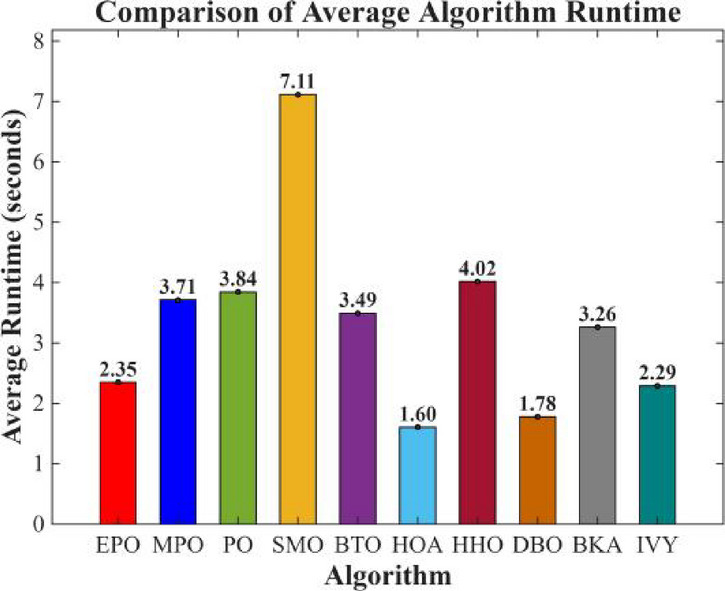
Comparison of average runtime.

**Fig. 23:**
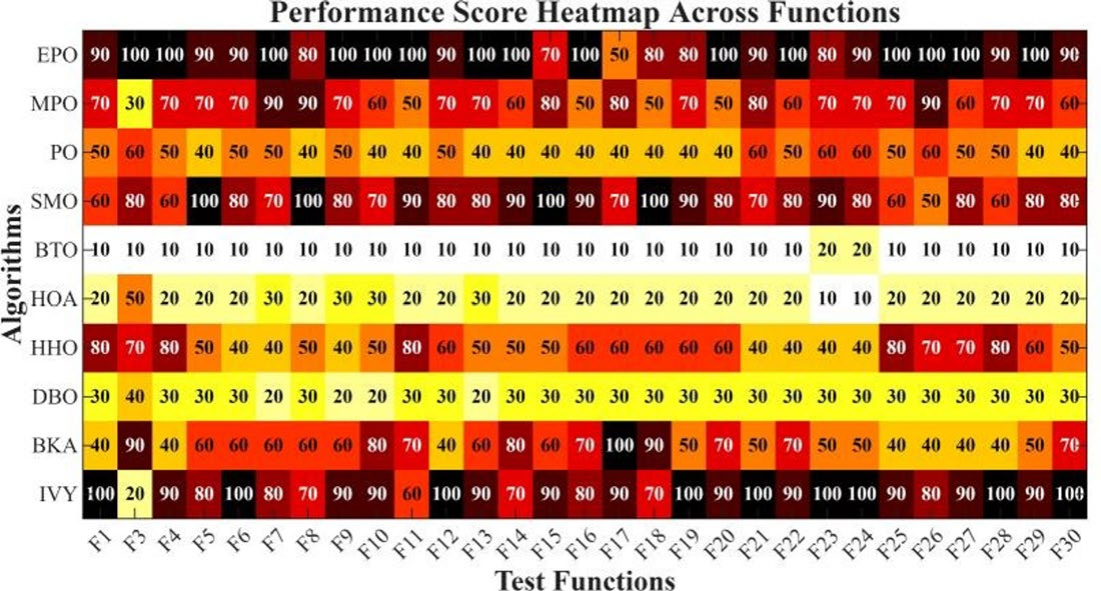
Performance score heatmap across functions.

Figures 20, 21, 22 and 23 show the radar chart, average ranking, comparison of average runtime, and performance score heatmap of the proposed EPO algorithm and comparison algorithms on the CEC2017 benchmark suite (D = 50).

As shown in Figs. 20, 21, 22 and 23, the proposed EPO algorithm achieves optimal performance on most functions., achieves the best overall performance with an average rank of 1.83, securing first place. It exhibits a statistically significant margin over subsequent algorithms, including IVY (average rank: 2.38) and SMO (3.07). The complete ranking order is as follows: EPO > IVY > SMO > MPO > HHO > BKA > PO > DBO > HOA > BTO.The complete ranking order of average runtime is as follows: HOA > DBO > EPO > BKA > BTO > MPO > PO > HHO > SMO.In summary, EPO achieves a balanced integration of performance and efficiency.

#### Convergence analysis

**Fig. 24:**
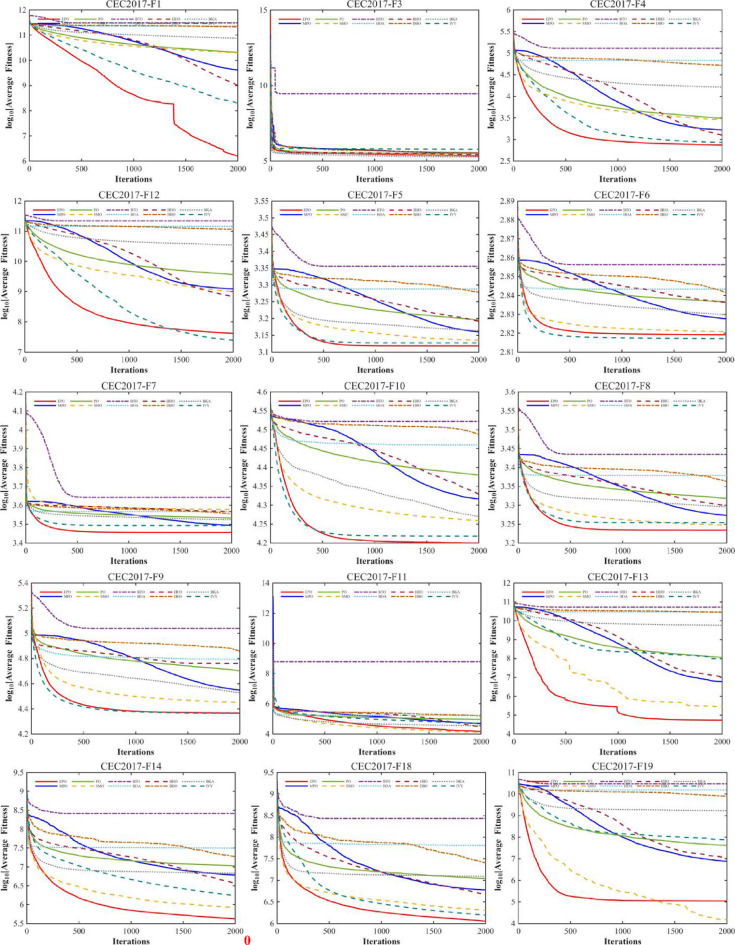

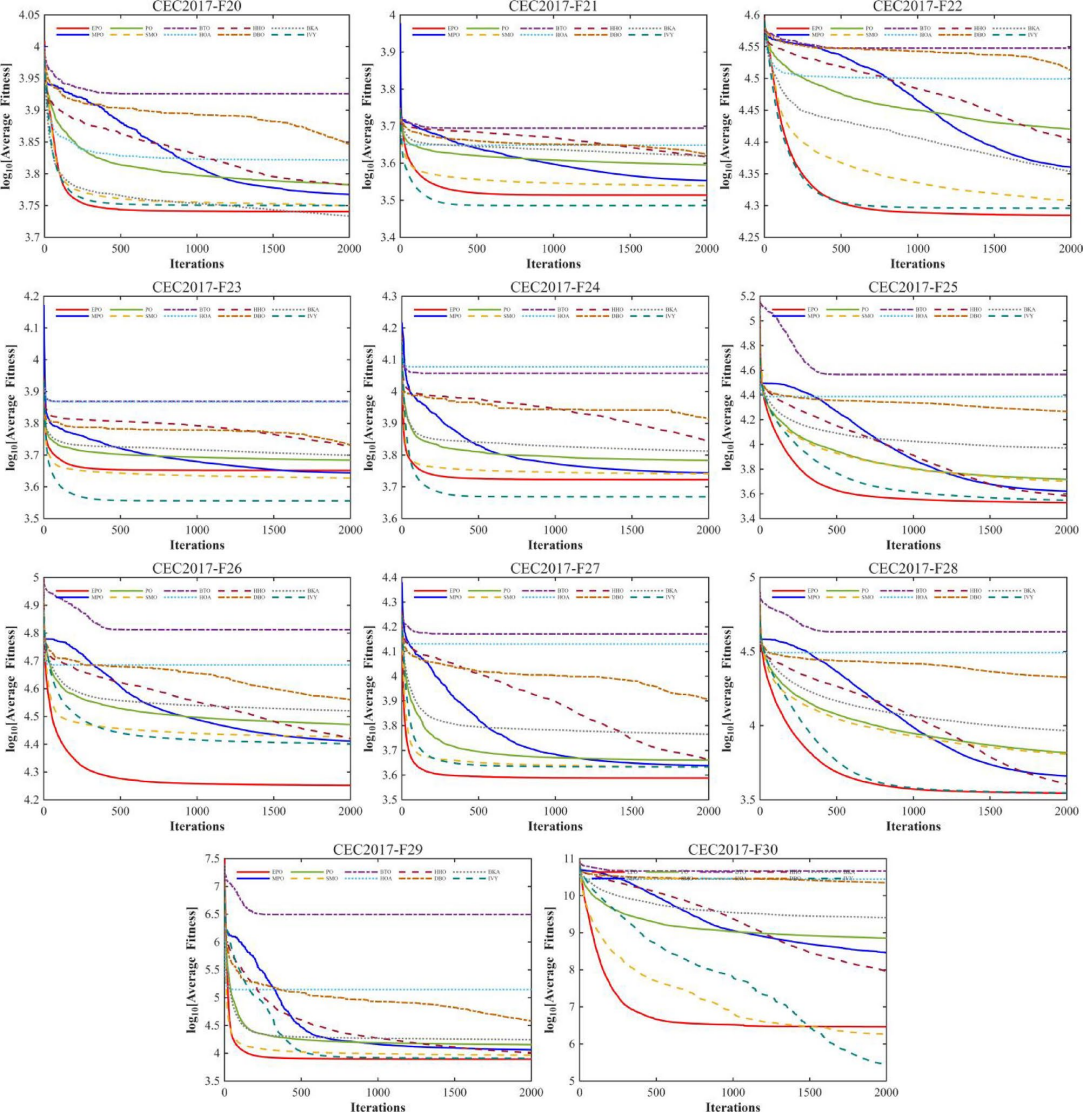
The convergence curves of the proposed technique and other algorithms for IEEE CEC2017 benchmark functions (Dim = 100).

A comprehensive comparative analysis was conducted using the 30-dimensional (D = 100) functions of the CEC2017 benchmark suite. The performance of the proposed algorithm was benchmarked against nine state-of-the-art metaheuristics, namely MPO, PO, SMO, BTO, HOA, HHO, DBO, BKA, and IVY. All algorithms were executed for 30 independent trials with a population size (Np) of 30 and a maximum of 1,000 iterations (Tmax). The comparative performance is visually illustrated through convergence curves (Fig. 24).

Experimental results on **the** 100-dimensional CEC2017 benchmark suite (F1–F30, excluding F2),shown in Fig. 24, reveal that the proposed Enhanced Particle Optimizer (EPO) demonstrates strong convergence efficiency and stability across diverse problem landscapes.

1. **Unimodal functions (F1**,** F3). **EPO achieves the best final accuracy on F1, with rapid convergence and continuous refinement throughout the search process. On F3 (Shifted and Rotated Rosenbrock), EPO converges quickly and remains stable, though its final precision is slightly inferior to BKA—a minor limitation in navigating the narrow, curved valley characteristic of this function.
2. **Simple multimodal functions (F4–F10). **EPO delivers outstanding performance: it obtains the best-known results on F4, F5, F7, F8, F9, and F10, often with sustained improvement until termination. Notably, on F9, although EPO achieves the highest accuracy, its convergence speed is marginally slower than IVY, indicating a trade-off between exploration thoroughness and initial exploitation rate.
3. **Hybrid functions (F11–F20). **EPO shows competitive yet mixed performance. It attains optimal or near-optimal solutions on F13, F14, F16, and F18, with F18 yielding the best result among all algorithms. However, on several other hybrid functions—**particularly** F11, F12, F15, F19, and F20—EPO is outperformed by SMO, IVY, or BKA in the later stages of optimization. For instance, on F15, despite fast initial convergence, EPO’s final accuracy lags behind both SMO and IVY; on F20, BKA demonstrates superior late-stage refinement. These findings suggest that while EPO effectively handles moderate hybrid complexity, it faces challenges in problems with highly heterogeneous subcomponents and strong inter-variable coupling at 100 dimensions.
4. **Composition functions (F21–F30). **EPO demonstrates remarkable robustness. It achieves the best performance on F22, F25–F29, consistently exhibiting rapid convergence coupled with continuous or stable high-precision outcomes. On F27 and F29, EPO not only reaches the lowest objective values but also maintains exceptional stability. Conversely, on F21, F23, F24, and F30, IVY emerges as the top performer, with SMO and MPO occasionally surpassing EPO in the final iterations (e.g., F23). Nevertheless, EPO’s dominance across five out of ten composition functions underscores its effectiveness in tackling high-dimensional, non-separable, rotated, and shifted composite landscapes.

Overall, EPO significantly outperforms the original PO and the alternative PO variant (MPO) across the majority of test cases, validating the efficacy of the proposed enhancements. While SMO and IVY exhibit superior precision on certain hybrid and **composition** functions, EPO consistently balances fast convergence, solution accuracy, and stability—particularly in unimodal, simple multimodal, and mid-to-high complexity composition problems—making it a highly competitive optimizer for large-scale real-world applications.

#### Analysis of box plot results

**Fig. 25:**
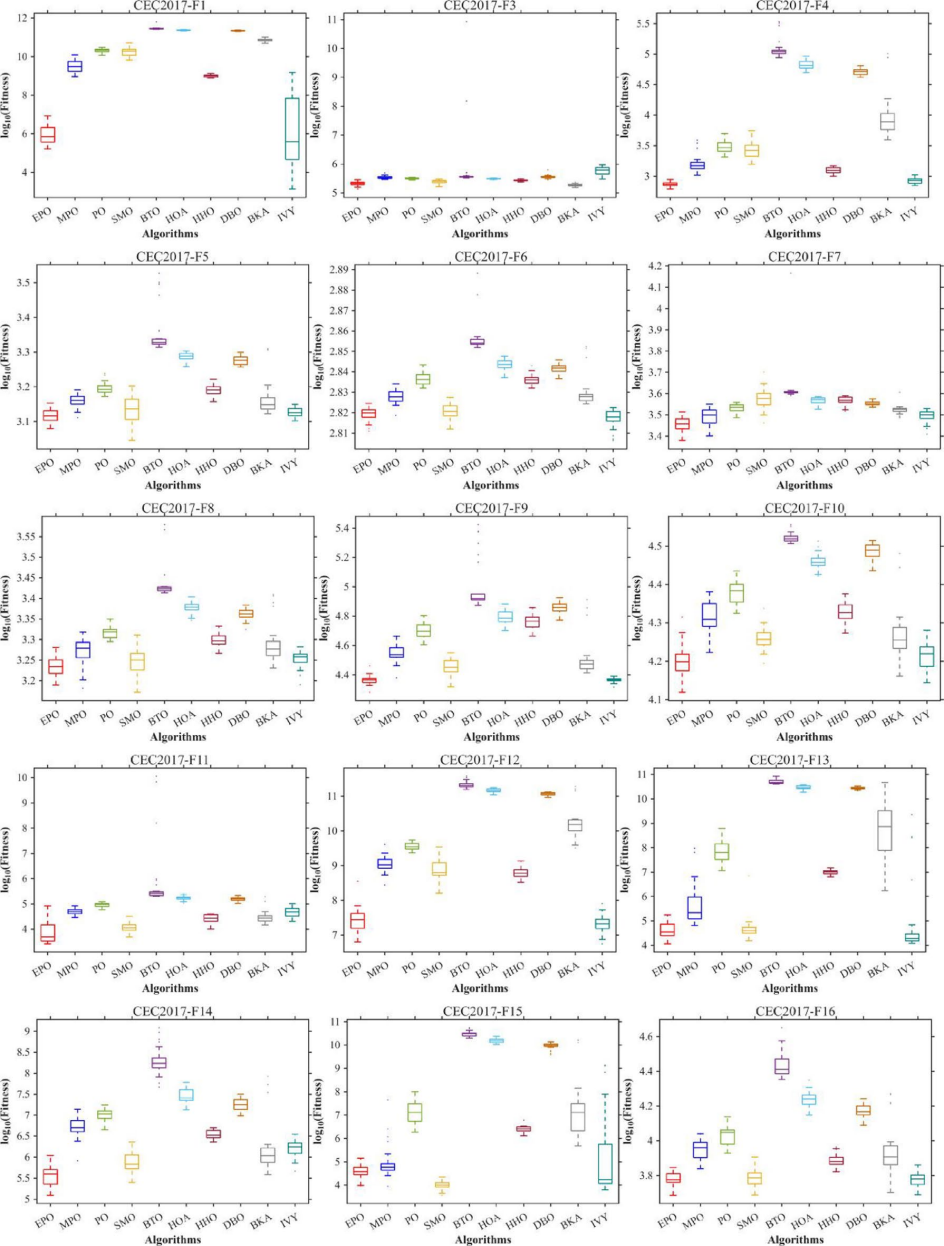

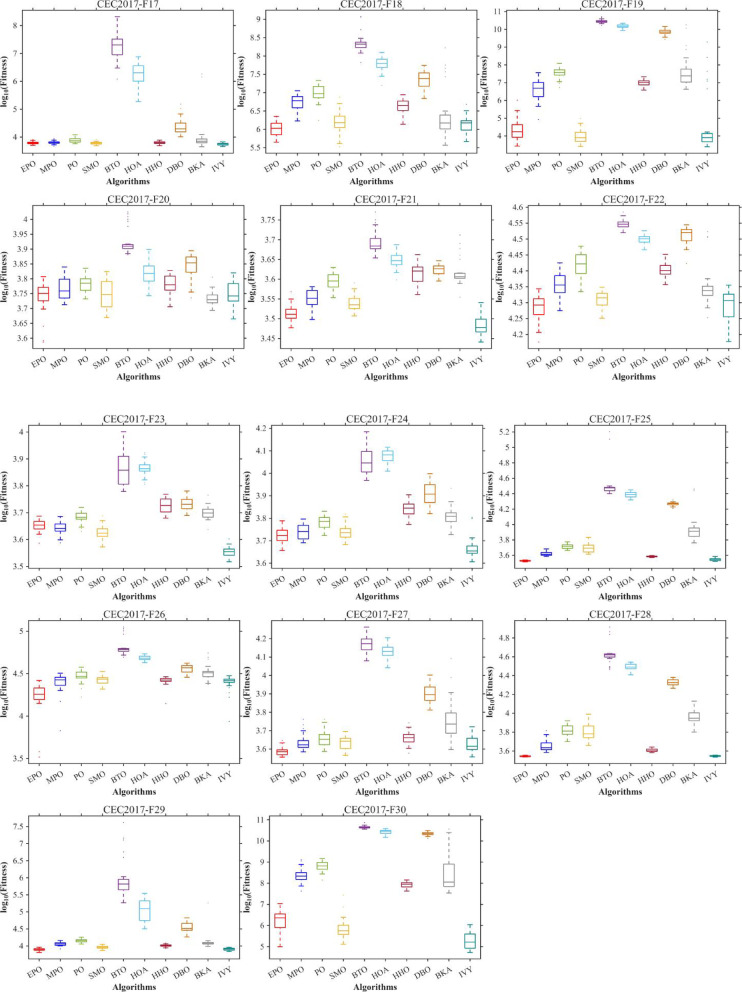
Boxplots for the proposed technique and other algorithms for IEEE CEC2017 benchmark functions.

Figure 25 is the box graph obtained after 30 runs of the algorithm, the box plots graphically illustrate the performance distribution of EPO relative to the canonical PO algorithm and other benchmark metaheuristics.

As shown in Fig. 25, the EPO algorithm demonstrates outstanding overall optimization performance. In a comprehensive comparison against all benchmark algorithms, EPO achieves the lowest median error on 16 functions, including most multimodal functions from F4 to F11 as well as complex composite functions such as F22 and F25–F29. Furthermore, EPO attains the smallest interquartile range (IQR) on F4, F27, and F28, reflecting its superior accuracy and stability. Additionally, EPO exhibits zero outliers on 15 functions, confirming its strong robustness.

Notably, when directly compared with the PO algorithm, EPO yields lower median errors on all 30 test functions, achieves smaller IQRs on 14 functions, and produces no outliers on 18 functions. These results collectively demonstrate that EPO not only leads in overall benchmark performance but also significantly outperforms PO, making it highly suitable for high-dimensional and complex optimization problems due to its exceptional accuracy, stability, and reliability.

#### Analysis of Wilcoxon rank sum test results

**Fig. 26:**
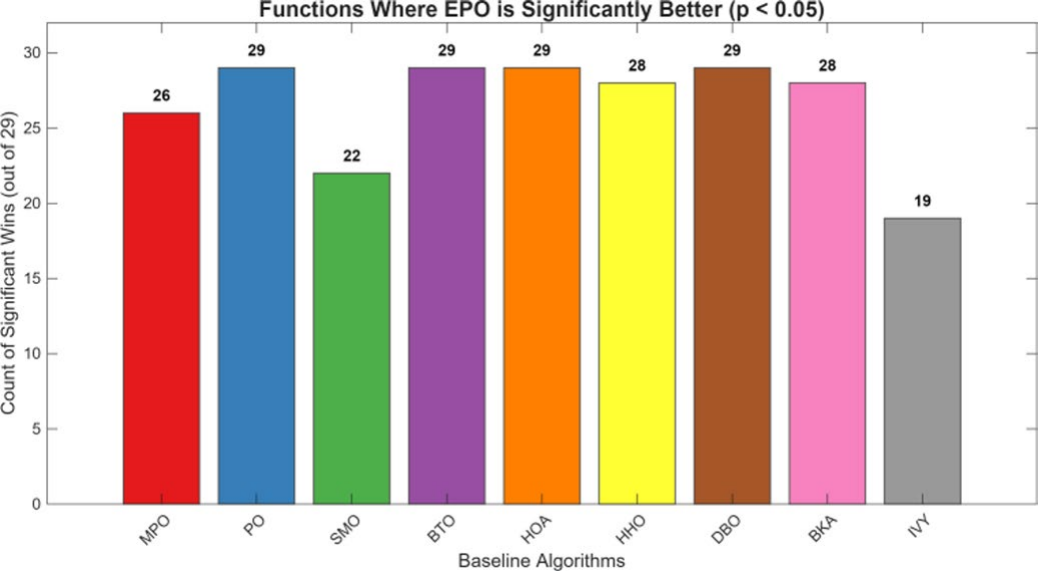
Boxplots for the proposed technique and other algorithms for IEEE CEC2017 benchmark functions (Dim = 100).

To rigorously evaluate the performance advantage of the proposed EPO algorithm, pairwise Wilcoxon rank-sum tests were conducted at a significance level of α = 0.05 between EPO and nine state-of-the-art optimization algorithms—MPO, PO, SMO, BTO, HOA, HHO, DBO, BKA, and IVY—across the 29 CEC2017 benchmark functions. The results of the Wilcoxon rank-sum tests are presented in Table 9; Fig. 26.

**Table 9:** Wilcoxon rank sum test results (Dim = 100).

Function	MPO	PO	SMO	BTO	HOA	HHO	DBO	BKA	IVY
F1	3.0199E-11	3.0199E-11	3.0199E-11	3.0199E-11	3.0199E-11	3.0199E-11	3.0199E-11	3.0199E-11	**7.3940E-01**
F3	3.0199E-11	4.0772E-11	2.2360E-02	3.0199E-11	4.0772E-11	2.5721E-07	3.3384E-11	5.6073E-05	3.0199E-11
F4	3.0199E-11	3.0199E-11	3.0199E-11	3.0199E-11	3.0199E-11	3.0199E-11	3.0199E-11	3.0199E-11	2.8790E-06
F5	3.4971E-09	3.0199E-11	**6.3533E-02**	3.0199E-11	3.0199E-11	3.0199E-11	3.0199E-11	2.1959E-07	4.8413E-02
F6	2.6099E-10	3.0199E-11	**1.6687E-01**	3.0199E-11	3.0199E-11	3.0199E-11	3.0199E-11	3.6897E-11	**8.5000E-02**
F7	5.2640E-04	1.0937E-10	2.1544E-10	3.0199E-11	3.0199E-11	3.0199E-11	3.0199E-11	2.6099E-10	3.5923E-05
F8	2.3168E-06	3.0199E-11	3.6439E-02	3.0199E-11	3.0199E-11	4.0772E-11	3.0199E-11	1.5581E-08	6.5486E-04
F9	5.4941E-11	3.0199E-11	2.0023E-06	3.0199E-11	3.0199E-11	3.0199E-11	3.0199E-11	8.9934E-11	**8.6499E-01**
F10	4.1997E-10	3.0199E-11	1.4733E-07	3.0199E-11	3.0199E-11	8.9934E-11	3.0199E-11	5.4620E-06	4.8413E-02
F11	4.6856E-08	1.6132E-10	6.0971E-03	3.0199E-11	3.0199E-11	8.8829E-06	3.0199E-11	4.7445E-06	6.5277E-08
F12	3.3384E-11	3.0199E-11	5.4941E-11	3.0199E-11	3.0199E-11	3.3384E-11	3.0199E-11	3.0199E-11	**1.2967E-01**
F13	4.5726E-09	3.0199E-11	**8.8830E-01**	3.0199E-11	3.0199E-11	3.0199E-11	3.0199E-11	3.0199E-11	1.3703E-03
F14	4.0772E-11	3.0199E-11	3.8307E-05	3.0199E-11	3.0199E-11	3.0199E-11	3.0199E-11	9.2603E-09	1.9568E-10
F15	3.8481E-03	3.0199E-11	2.6695E-09	3.0199E-11	3.0199E-11	3.0199E-11	3.0199E-11	3.0199E-11	3.2651E-02
F16	4.0772E-11	3.0199E-11	**8.6499E-01**	3.0199E-11	3.0199E-11	2.1544E-10	3.0199E-11	2.2273E-09	**9.9410E-01**
F17	**2.1156E-01**	3.3681E-05	**2.4581E-01**	3.0199E-11	3.0199E-11	**4.9178E-01**	3.0199E-11	4.2259E-03	3.7704E-04
F18	4.9752E-11	4.9752E-11	1.2732E-02	3.0199E-11	3.0199E-11	9.9186E-11	3.0199E-11	1.0763E-02	2.8129E-02
F19	1.2057E-10	3.0199E-11	1.2732E-02	3.0199E-11	3.0199E-11	3.0199E-11	3.0199E-11	3.0199E-11	**9.6263E-02**
F20	**1.0233E-01**	1.4067E-04	**7.3940E-01**	3.0199E-11	1.6947E-09	1.4932E-04	1.2870E-09	**7.4827E-02**	**7.2827E-01**
F21	7.6950E-08	3.6897E-11	7.2208E-06	3.0199E-11	3.0199E-11	3.3384E-11	3.0199E-11	3.3384E-11	1.0407E-04
F22	3.4971E-09	3.6897E-11	1.0763E-02	3.0199E-11	3.0199E-11	3.0199E-11	3.0199E-11	1.0277E-06	**2.2257E-01**
F23	**1.3345E-01**	7.0430E-07	1.4067E-04	3.0199E-11	3.0199E-11	4.5043E-11	3.0199E-11	8.8910E-10	3.6897E-11
F24	2.8129E-02	1.2023E-08	**5.3685E-02**	3.0199E-11	3.0199E-11	3.6897E-11	3.0199E-11	2.8716E-10	3.2555E-07
F25	3.0199E-11	3.0199E-11	3.0199E-11	3.0199E-11	3.0199E-11	3.0199E-11	3.0199E-11	3.0199E-11	4.4440E-07
F26	3.5201E-07	1.5465E-09	7.1186E-09	3.0199E-11	3.0199E-11	4.5726E-09	3.0199E-11	6.6955E-11	2.5721E-07
F27	3.8053E-07	3.1967E-09	2.6784E-06	3.0199E-11	3.0199E-11	1.8567E-09	3.0199E-11	9.9186E-11	1.8682E-05
F28	3.0199E-11	3.0199E-11	3.0199E-11	3.0199E-11	3.0199E-11	3.0199E-11	3.0199E-11	3.0199E-11	**2.5188E-01**
F29	6.6955E-11	3.0199E-11	2.6015E-08	3.0199E-11	3.0199E-11	1.4643E-10	3.0199E-11	3.0199E-11	**9.9258E-02**
F30	3.0199E-11	3.0199E-11	1.6798E-03	3.0199E-11	3.0199E-11	3.0199E-11	3.0199E-11	3.0199E-11	3.2555E-07

The results in Table 9; Fig. 26 indicate that, out of a total of 261 statistical comparisons, EPO achieved significantly better performance in 239 cases (91.6%) at the significance level of *p* < 0.05. Specifically, EPO obtained win rates exceeding 93% against PO, BTO, HOA, HHO, DBO, and BKA, and demonstrated statistically superior performance on all 29 functions (100% win rate) when compared to PO, BTO, HOA, and DBO. In contrast, IVY exhibited relatively competitive performance, showing no significant difference from EPO on at least 25% of the functions; nevertheless, EPO still significantly outperformed IVY on 19 out of 29 functions (65.5%). In summary, EPO not only exhibits highly significant performance advantages in the vast majority of test scenarios but also demonstrates exceptional robustness across diverse optimization problems.

## EPO for wireless sensor network node deployment

### WSN coverage optimization: a mathematical model

In this study, the Wireless Sensor Network (WSN) deployment is formulated within a two-dimensional monitoring area of size $L \times W$. The model operates under the following key assumptions: all sensor nodes are homogeneous with an identical circular sensing range^58^, defined by a sensing radius ${R_{\text{s}}}$. To maintain network-wide connectivity, the communication radius ${R_c}$is set to twice the sensing radius, i.e., ${R_c}$= 2${R_{\text{s}}}$.The problem involves a set of sensor nodes, $V=\{ {B_1},{B_2}, \cdots ,{B_S}\}$, ${B_i}$’s coordinates is ${B_i} {x_i} {y_i}$. To evaluate coverage, the monitoring area is discretized into a finite set of $m \times n$target points,${A_j}=({x_j},{y_j}),j \in \{ 1,2, \cdots ,m \times n\}$. Coverage is determined using a Boolean sensing model, where a target point ${A_j}$ is considered covered if it lies within the sensing radius${R_{\text{s}}}$ of at least one sensor node.This coverage condition is based on the Euclidean distance between a sensor node at coordinates ${B_i}$ and a target point ${A_j}$ which is defined as:

18 $$d({B_i},{A_j})=\sqrt {{{({x_i} - {x_j})}^2}+{{({y_i} - {y_j})}^2}}$$

where $d({B_i},{A_j})$ is the Euclidean distance. ${A_j} {x_j} {y_j}$, ${B_i} {x_i} {y_i}$are the coordinates of the target point and the sensor node, respectively.

This study adopts a Boolean sensing model. If the distance between the sensor node ${B_i} {x_i} {y_i}$ and the target point ${A_j} {x_j} {y_j}$ is less than or equal to the sensing radius ${R_{\text{s}}}$, the detection probability is 1; otherwise, it is 0. The probability that sensor node ${B_i} {x_i} {y_i}$ covers the target point ${A_j} {x_j} {y_j}$ is expressed as follows

19 $$P({B_i},{A_j})=\left\{ {\begin{array}{*{20}{l}} 1&{d({B_i},{A_j}) \leqslant {R_s}} \\ 0&{d({B_i},{A_j})>{R_s}} \end{array}} \right.$$

The coverage of a target point${A_j}$ by an individual sensor ${B_i}$ is determined by a binary condition: If $d({B_i},{A_j}) \leqslant {R_s}$, the sensor ${B_i}$successfully covers the target point ${A_j}$.If $d({B_i},{A_j})>{R_s}$, the sensor ${B_i}$ fails to cover the target point ${A_j}$.Since a single point within the monitoring area can be simultaneously sensed by multiple sensor nodes, the collective probability that a target point ${A_j}$ is covered by the entire set of nodes, given by:

20 $${P_c}(V,{A_j})=1 - \prod\limits_{{i=1}}^{n} {(1 - P(} {B_i},{A_j}))$$

The total coverage rate for the deployment region, achieved by the node set *V*, is formulated as:

21 $${R_{cover}}=\frac{{\sum\limits_{{j=1}}^{{m \times n}} {{P_c}} (V,{A_j})}}{{m \times n}}$$

The objective of WSN deployment optimization is to maximize the monitoring coverage subject to the constraint of using a minimal number of sensors. This can be formulated as a constrained engineering optimization problem, as shown in the following equation:

22 $$f=\hbox{max} ({R_{cover}})=\hbox{min} (1 - {R_{cover}})$$

23 $${\text{s}}{\text{.t}}{\text{.}}\left\{ {\begin{array}{*{20}{c}} {{g_1} = \sum\limits_{j = 1}^{m \times n} p (V,{A_j})0} \\ {{g_2} = \sum\limits_{j = 1}^{m \times n} p (V,{A_j}) - m \times n0} \\ {{g_3} = d({B_i},{A_j}) - {R_s}0} \end{array}} \right.$$

### WSN coverage optimization: optimization results

To investigate the efficiency of the EPO algorithm in optimizing WSN coverage, three distinct case studies were conducted. In each case, the performance of EPO was benchmarked against the standard PO algorithm. The fundamental parameters for Case 1, Case 2, and Case 3 are detailed in Table 10.

**Table 10:** Basic parameters for WSN coverage optimization.

Parameters	Case 1	Case 2	Case 3
Number of Iteration	500	500	500
Number of nodes	20	30	40
Node radius	12	12	12
Coverage area	100 m × 100 m	100 m × 100 m	100 m × 100 m

### Case 1: focuses on optimizing the coverage rate and connectivity for a 20-node WSN

**Fig. 27:**
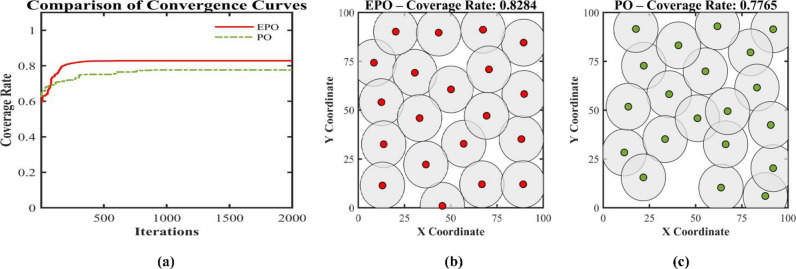
Comparative analysis of coverage in WSN (*N* = 20). (**a**) Comparison of Final Coverage Rates (**b**) EPO-Optimal Node Layouts (**c**) PO-Optimal Node Layouts.

The EPO and PO algorithms were employed to optimize the coverage problem for a Wireless Sensor Network (WSN) composed of 20 nodes, with the network coverage rate serving as the fitness function. The resulting optimized coverage maps and their corresponding fitness values are presented in Fig. 27. Specifically, Fig. 27(a) displays the fitness values for both algorithms, Fig. 27(b) shows the node deployment optimized by the EPO algorithm, and Fig. 27(c) illustrates the deployment optimized by the PO algorithm.

As depicted in Fig. 27(a), the PO algorithm achieved a coverage rate of 0.7765, whereas the EPO algorithm demonstrated significant superiority, reaching a maximum coverage rate of 0.8284, proving most effective in maximizing the monitored area. Figures 27(b) and (c) visualize the coverage maps for the 20-node network deployed within a 100 × 100 region. In these figures, the gray areas represent the covered zones, i.e., the regions effectively monitored by the sensor nodes. The red dots indicate the node positions, while the remaining areas highlight the uncovered regions, commonly referred to as “coverage holes.“A visual comparison of Figs. 27(b) and (c) reveals that the deployment optimized by the EPO algorithm results in significantly smaller uncovered regions and a more uniform node distribution compared to the outcome of the PO algorithm. Nevertheless, the maximum coverage rate in Fig. 27 is only 0.8284. This limitation is primarily attributed to the small sensing radius and the insufficient number of deployed nodes.

### Case 2: focuses on optimizing the coverage rate and connectivity for a 30-node WSN

**Fig. 28:**
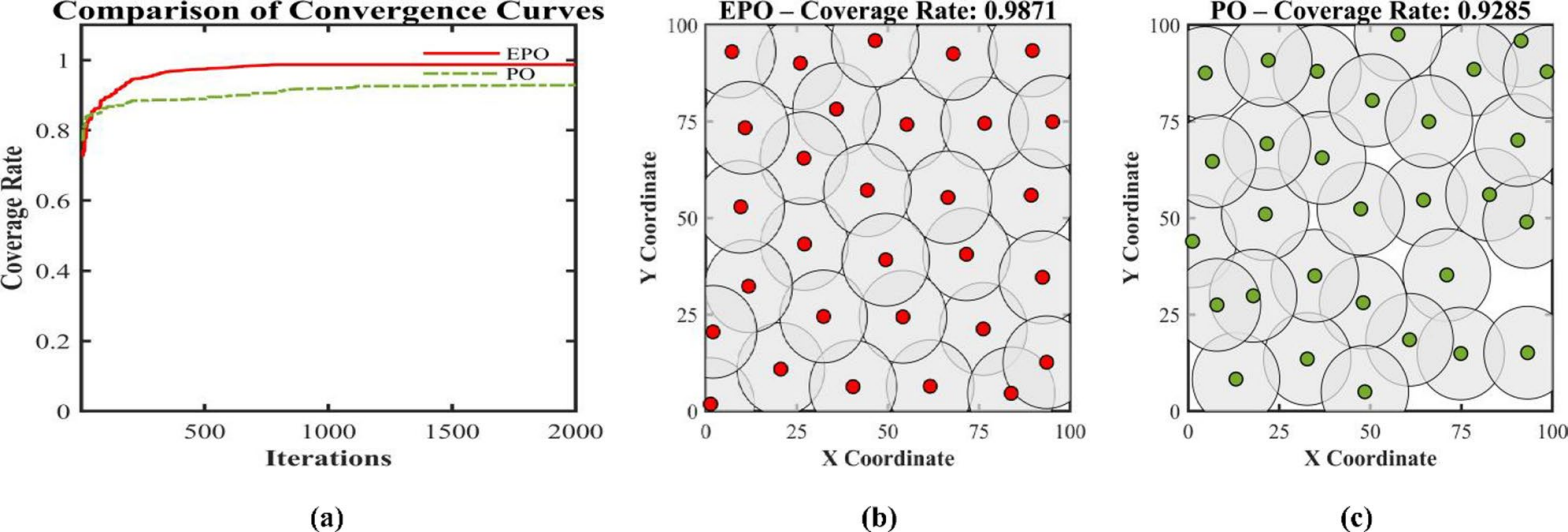
Comparative analysis of coverage in WSN (*N* = 30). (**a**) Comparison of Final Coverage Rates (**b**) EPO-Optimal Node Layouts (**c**) PO-Optimal Node Layouts.

The EPO and PO algorithms were employed to optimize the coverage problem for a Wireless Sensor Network (WSN) composed of 30 nodes, with the network coverage rate serving as the fitness function. The optimized coverage maps and their corresponding fitness values are presented in Fig. 28. Specifically, Fig. 28(a) displays the fitness values for both algorithms, Fig. 28(b) shows the node deployment optimized by the EPO algorithm, and Fig. 28(c) illustrates the deployment optimized by the PO algorithm.

As depicted in Fig. 28(a), the PO algorithm achieved a coverage rate of 0.9285, whereas the EPO algorithm demonstrated significant superiority, reaching a maximum coverage rate of 0.9871, proving most effective in maximizing the monitored area. A visual comparison of Figs. 28(b) and (c) reveals that the deployment optimized by the EPO algorithm results in significantly smaller uncovered regions and a more uniform node distribution compared to the outcome of the PO algorithm.

### Case 3: focuses on optimizing the coverage rate and connectivity for a 40-node WSN

**Fig. 29:**
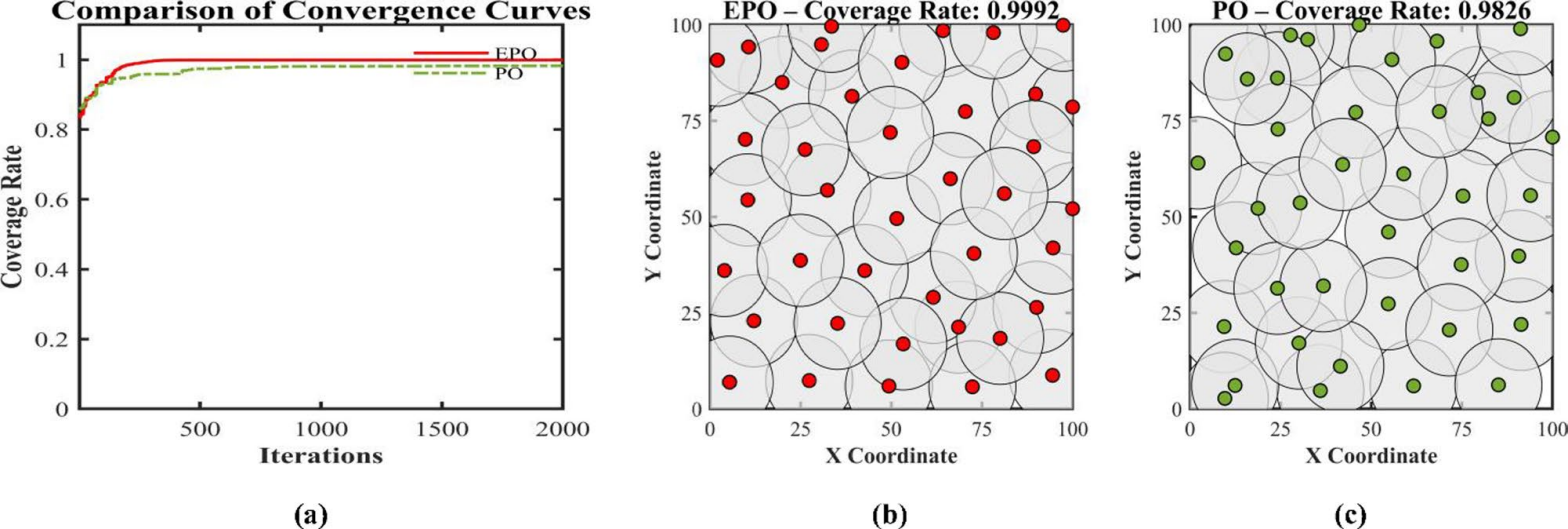
Comparative analysis of coverage in WSN (*N* = 40). (**a**) Comparison of Final Coverage Rates (**b**) EPO-Optimal Node Layouts (**c**) PO-Optimal Node Layouts.

The EPO and PO algorithms were employed to optimize the coverage problem for a Wireless Sensor Network (WSN) composed of 40 nodes, with the network coverage rate serving as the fitness function. The optimized coverage maps and their corresponding fitness values are presented in Fig. 29. Specifically, Fig. 29(a) displays the fitness values for both algorithms, Fig. 29(b) shows the node deployment optimized by the EPO algorithm, and Fig. 29(c) illustrates the deployment optimized by the PO algorithm.

As depicted in Fig. 29(a), the PO algorithm achieved a coverage rate of 0.9826, whereas the EPO algorithm demonstrated significant superiority, reaching a maximum coverage rate of 0.9992, proving most effective in maximizing the monitored area. A visual comparison of Fig. 29(b) and (c) reveals that the deployment optimized by the EPO algorithm results in significantly smaller uncovered regions and a more uniform node distribution compared to the outcome of the PO algorithm.

## Conclusion

This paper addresses the critical limitations of the original parrot Optimizer (PO)—namely, insufficient initial population diversity, susceptibility to premature convergence, slow convergence speed, and the absence of an effective restart mechanism—by proposing the Enhanced parrot Optimizer (EPO), a novel metaheuristic algorithm that synergistically integrates five key strategies: chaotic initialization, a risk-aware vigilance mechanism, a nonlinear decay factor, a dual-layer behavioral judgment system, and a hybrid TDE-based restart scheme. The efficacy of EPO is rigorously evaluated on the CEC2017 benchmark suite across 30, 50, and 100 dimensions against eleven state-of-the-art metaheuristic algorithms, as well as on the real-world Wireless Sensor Network (WSN) node deployment problem. The experimental results demonstrate the following:

(1) EPO effectively overcomes fundamental deficiencies of PO, particularly its inability to adaptively balance exploration and exploitation, its tendency toward premature convergence, and its lack of a controlled transition between search phases. This is achieved through a dynamically coordinated search process that begins with extensive global exploration and progressively shifts toward refined local exploitation, thereby ensuring both solution quality and convergence reliability.

(2) EPO achieves the best mean ranking across all dimensional settings—2.31 at 30D, 2.03 at 50D, and 1.83 at 100D. Non-parametric Wilcoxon signed-rank tests at a significance level of α = 0.05 confirm its statistically significant superiority in 212 out of 261 pairwise comparisons (81.2%) at 30D, 225 (86.2%) at 50D, and 239 (91.6%) at 100D, highlighting its robustness, rapid convergence, and consistent performance across diverse problem scales.

(3) In practical applications, EPO consistently outperforms PO in WSN node deployment across varying network scales: coverage improves from 0.7765 to 0.8284 (+ 6.7%) with 20 nodes, from 0.9285 to 0.9871 (+ 6.3%) with 30 nodes, and from 0.9826 to 0.9992 (+ 1.7%) with 40 nodes. Moreover, the resulting node distributions are more uniform, with significantly fewer coverage holes, underscoring EPO’s engineering applicability.

(4) Collectively, these findings indicate that EPO not only resolves the core weaknesses of PO through intelligent integration of complementary strategies but also establishes a new state-of-the-art within parrot-inspired optimization, offering a reliable and efficient framework for both complex numerical optimization and real-world engineering challenges.

Despite its strong empirical performance, several limitations warrant acknowledgment. First, EPO exhibits moderate sensitivity to its control parameters, and the incorporation of chaotic maps introduces additional computational overhead, which may affect suitability in highly time-constrained scenarios. Second, the current experimental validation is limited to the CEC2017 benchmark suite and WSN coverage optimization; on a subset of CEC2017 functions, EPO still lags slightly behind certain top-performing competitors. Finally, its generalizability to broader problem classes—such as dynamic, constrained, or multi-objective optimization—remains unexplored. These limitations delineate the current scope of the proposed approach while simultaneously revealing promising directions for future research.

## Data Availability

All data generated or analyzed during this study are included in this manuscript.
